# Supplementary material for: The role of highly oxygenated organic molecules in the Boreal aerosol-cloud-climate system
Source: Nat Commun. 2019 Sep 25;10:4370. doi: 10.1038/s41467-019-12338-8 (PMC6761173; doi:10.1038/s41467-019-12338-8)
Supplement: Supplementary file 1 — Supplementary Information [file 41467_2019_12338_MOESM1_ESM.pdf]

Supplementary Information for: The role of highly oxygenated organic molecules in the Boreal aerosol-cloud-climate system, by Roldin et al.

Supplementary Figures

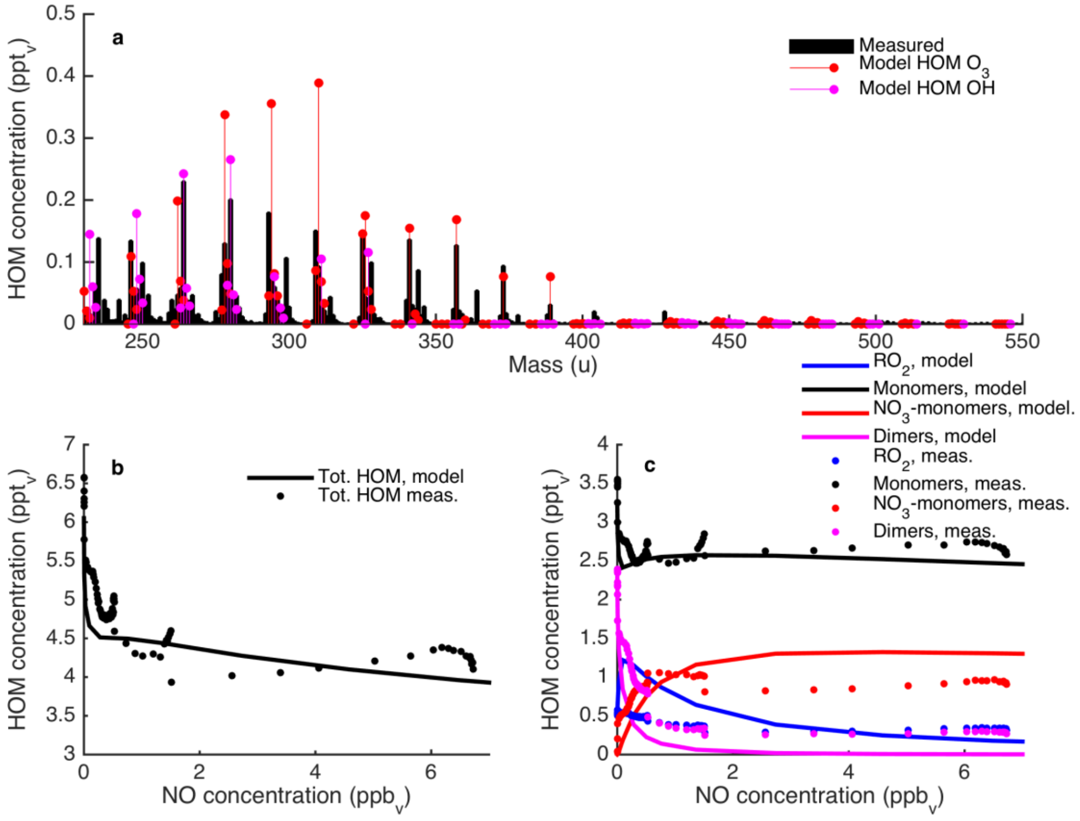

Supplementary Figure 1. Modelled and measured HOM concentrations during an *α*-pinene ozonolysis experiment in the Jülich Plant Atmosphere Chamber (JPAC) where the NO concentration was gradually increased while the *α*-pinene and O_3 concentrations were kept approximately constant at around 6 ppb$_v$ and 80 ppb$_v$ respectively. Panel **a** compares the modelled and measured HOM(g) mass spectrum at an NO concentration of ~1.5 ppb$_v$, panel **b** shows how the modelled and measured total HOM(g) concentration change when the NO concentration increases and panel **c** shows the modelled and measured concentrations of closed-shell HOM monomers without NO_3 (red), with NO_3 (blue) and dimers (purple). In **a** the modelled HOM mass spectrum is divided into one for the HOM species formed via ozonolysis of *α*-pinene and one for OH oxidation of *α*-pinene.

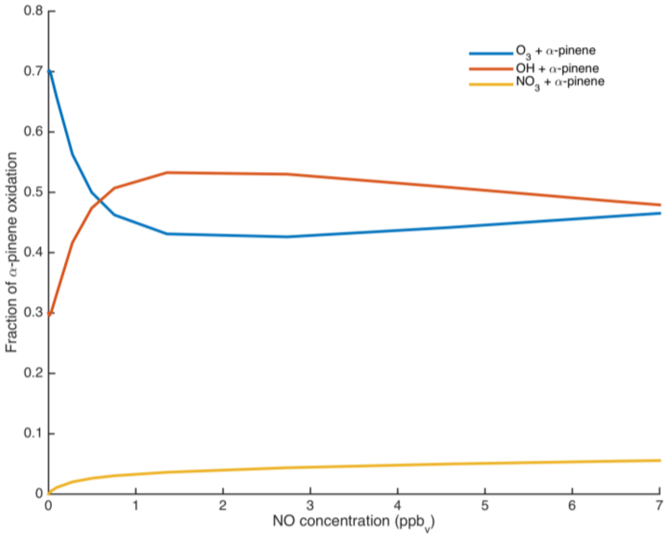

Supplementary Figure 2. Modelled fraction of *α*-pinene that are oxidized by O_3, OH and NO_3 at variable NO concentrations in JPAC.

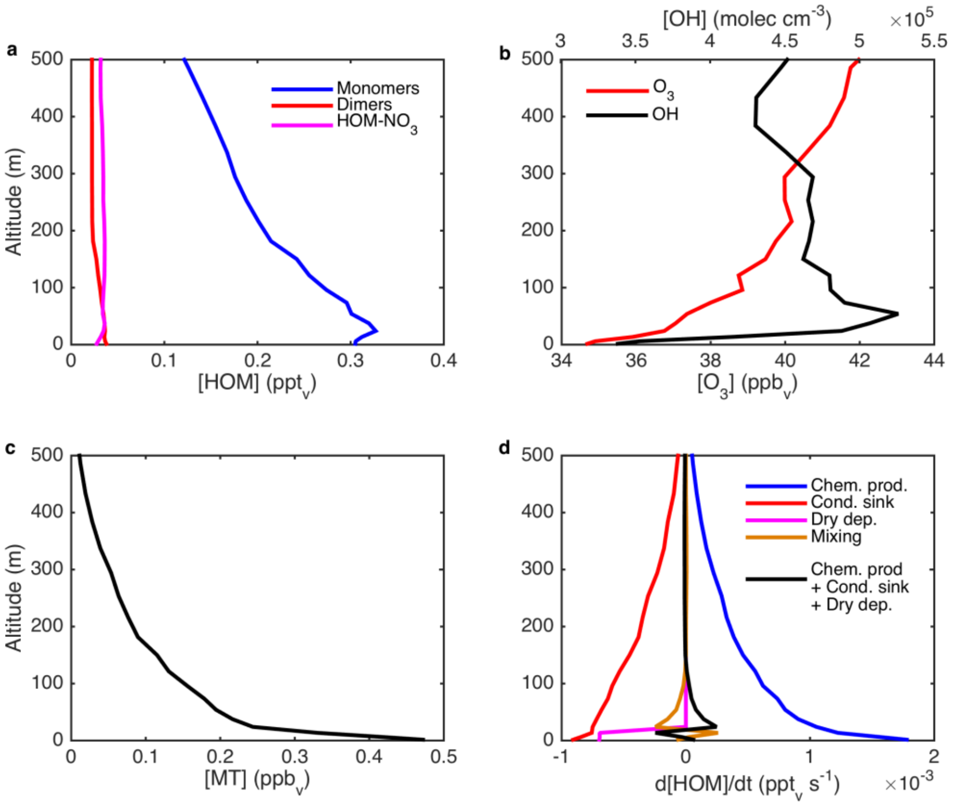

Supplementary Figure 3. Modelled median vertical concentration profiles 15th to 24th of May, 2013 of **a** HOM monomers without NO_3 groups, HOM dimers and HOM monomers with NO_3 groups, **b** O_3 and OH and **c** monoterpenes. Panel **d** shows the HOM chemical production rate, condensation sink, dry deposition loss rate, rate of change due to vertical mixing and the net rate of change due to chemical production, condensation and dry deposition. The generally higher HOM concentration at the top of the canopy than inside the canopy (**a**) causes a net downward flux (mixing) of HOM into the canopy, while above the canopy the net flux of HOM is upward (**d**).

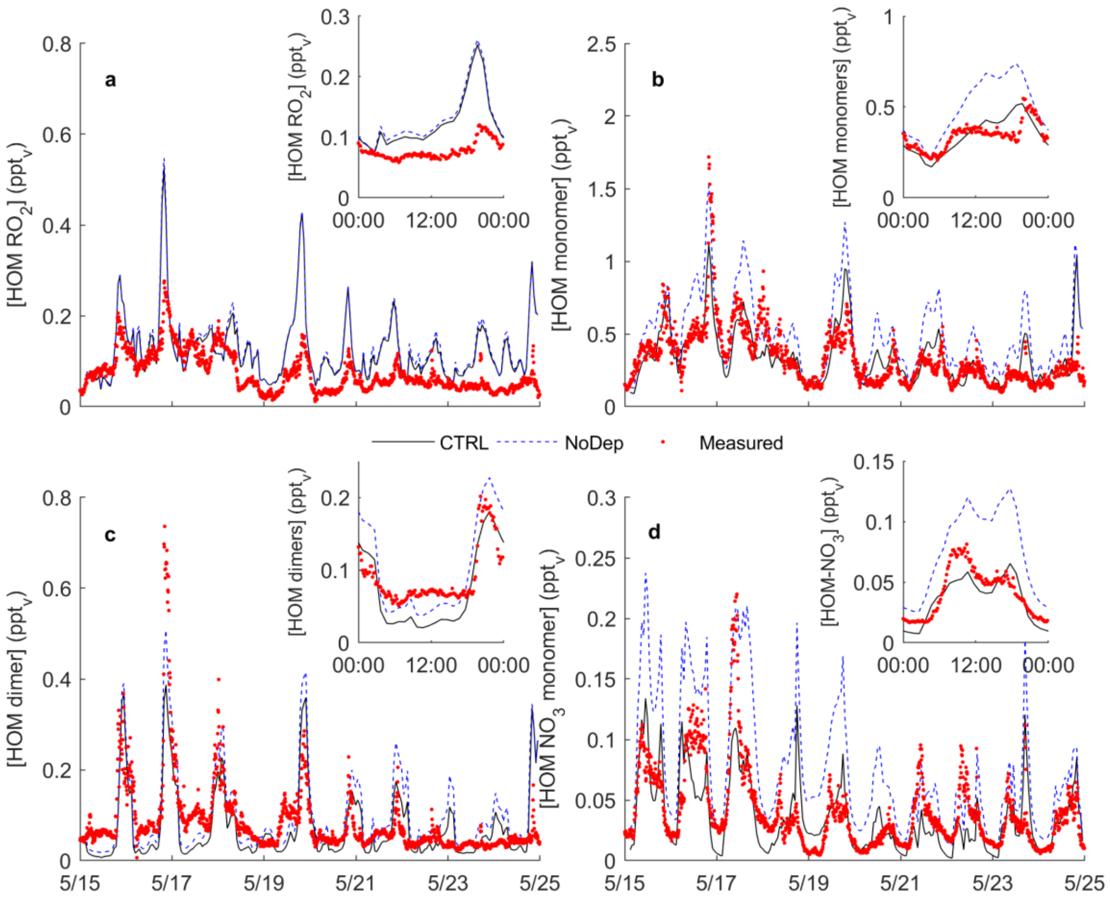

Supplementary Figure 4. Modelled and measured HOM gas-phase concentrations at the SMEAR II field station 15th to 24th of May, 2013. **a** HOM RO_2, **b** HOM(g) closed-shell monomers, **c** HOM(g) dimers, and **d** HOM organonitrate monomers (HOM-NO_3). The inset plots in each panel showing the modelled and measured mean diurnal trends of each HOM species type. The different model results are from the control run (CTRL), and a simulation without dry deposition of HOM (NoDep).

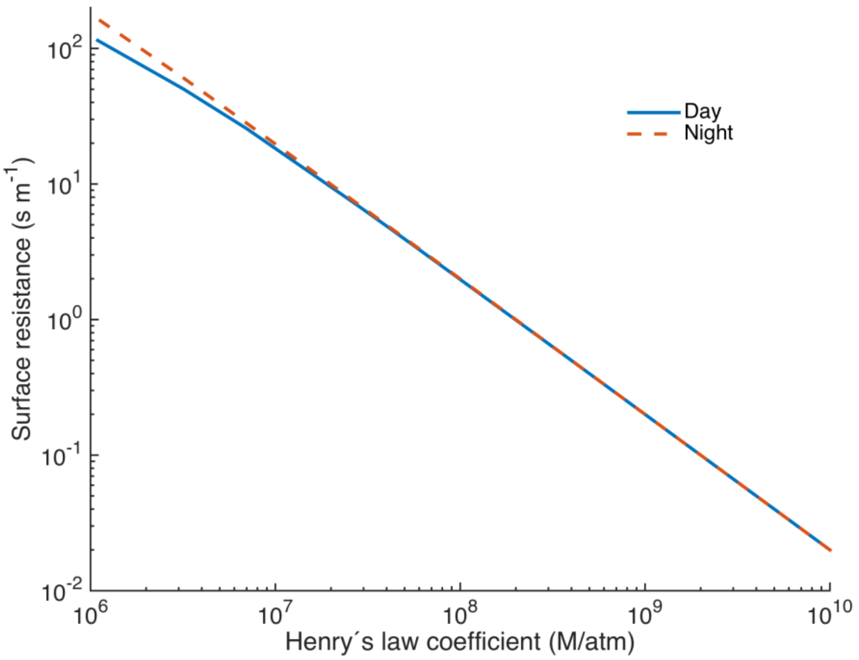

Supplementary Figure 5. Modelled daytime and nighttime surface resistances to uptake of gases as a function of their Henry's law coefficient (*H*). The *H* of HOM are typically larger than 10^8 M/atm (Supplementary Table 6) for which the surface resistance typically become less than 10 % of the combined aerodynamic and quasi-laminar resistance.

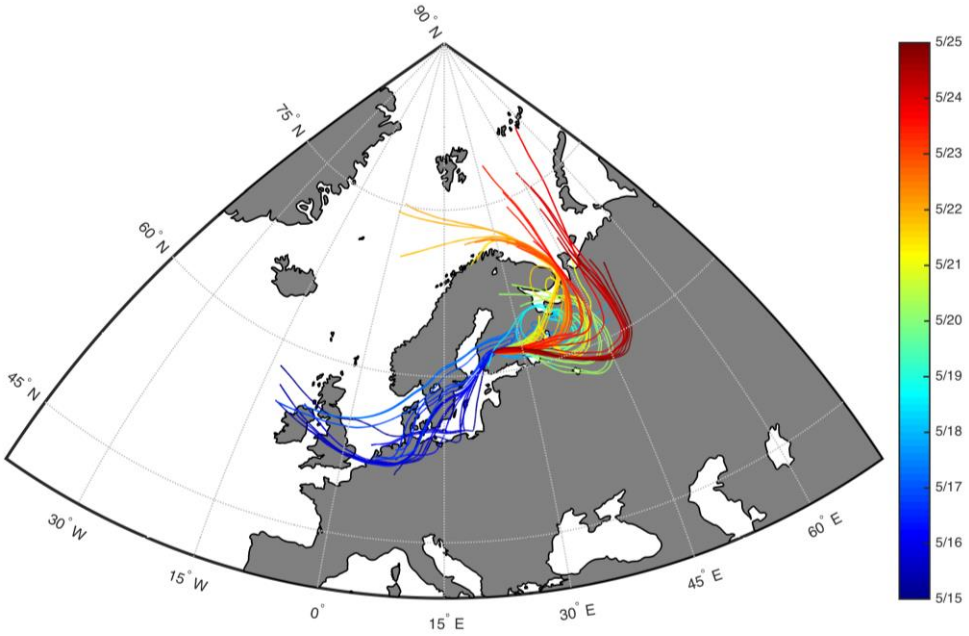

Supplementary Figure 6. 4-days HYSPLIT backward trajectories arriving at SMEAR II with 3 hours interval between 15th and 24th of May, 2013.

May).

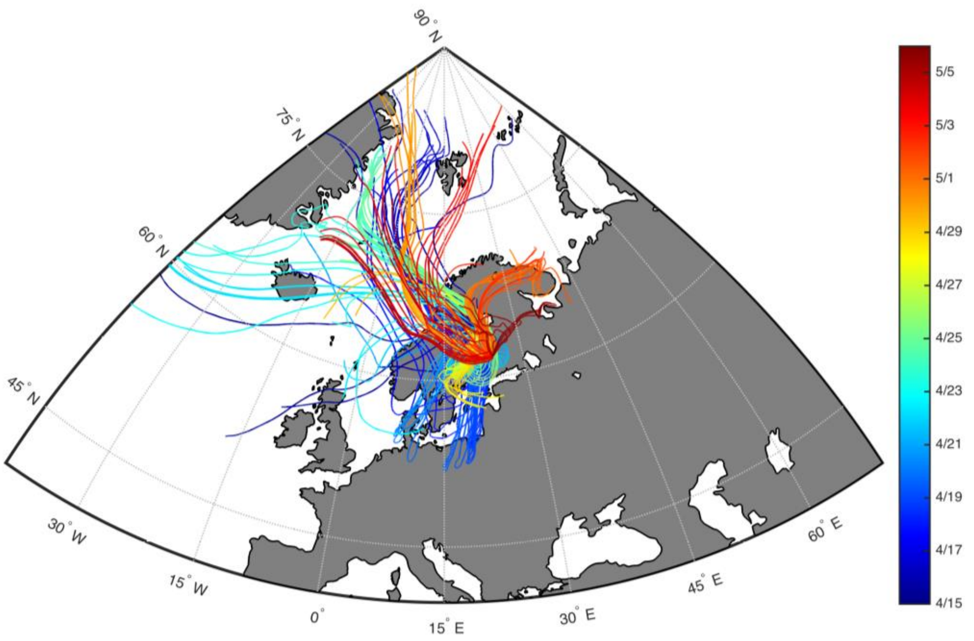

Supplementary Figure 7. 4-days HYSPLIT backward trajectories arriving at SMEAR II with 3 hours interval between 15th April and 5th of May, 2014.

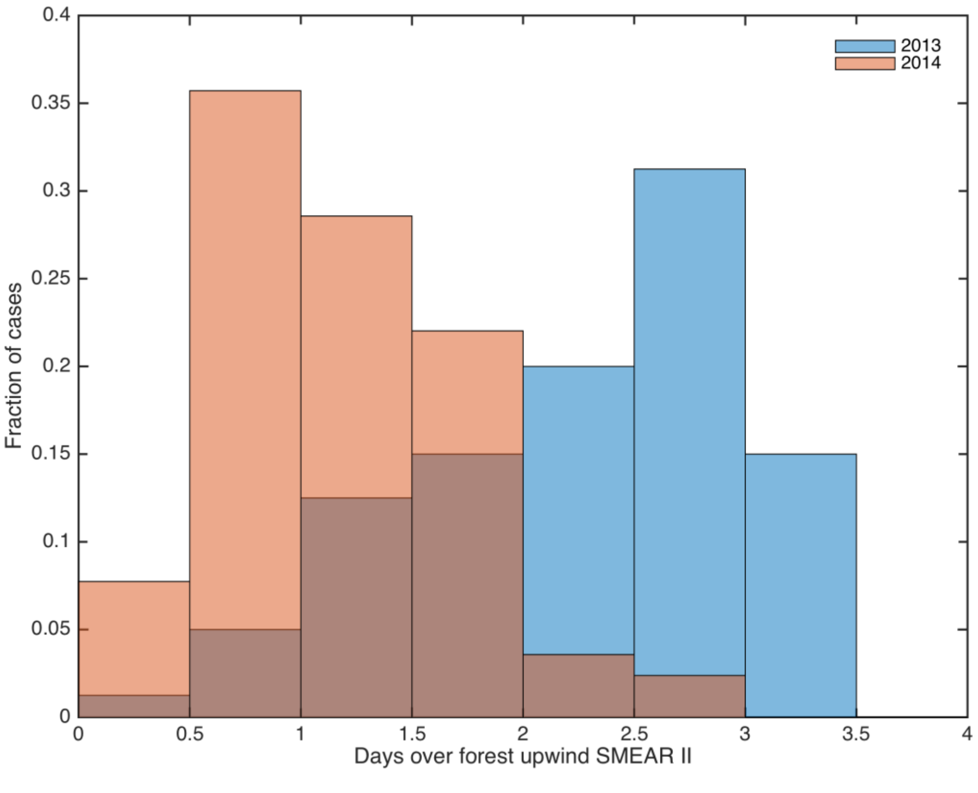

Supplementary Figure 8. Histograms with fraction of air masses that spend different amount of time over the boreal forest upwind SMEAR II during spring 2013 (15th to 24th of May) and spring 2014 (15th of April to 5th of May).

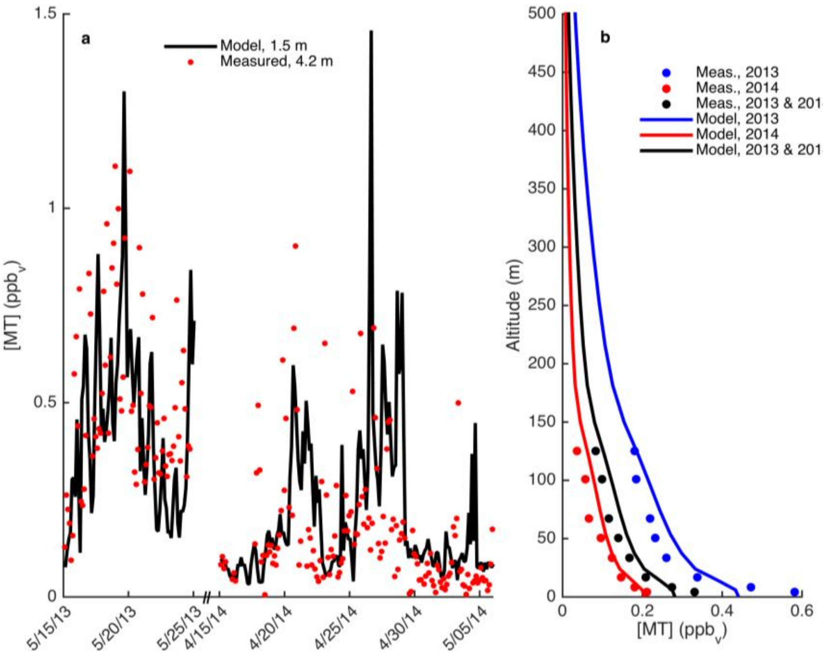

Supplementary Figure 9. Measured and modelled monoterpene concentrations ([MT]) at SMEAR II 15th to 24th of May 2013 and 15th of April to 5th of May 2014. Panel **a** shows the measured and modelled monoterpene concentrations close to the surface (Model: 1.5 m above ground, Measured: 4.2 m above ground). Panel **b** shows the measured and modelled average vertical concentration profiles for the 2013 period (15th to 24th of May), the 2014 period (15th of April to 5th of May) and the average for both periods. The monoterpene concentration was measured at 8 altitudes starting from 4.2 m above ground and extending up to 125 m. Both the model and measurements gives a factor of two higher average monoterpene concentrations during the 2013 period compared to the 2014 period.

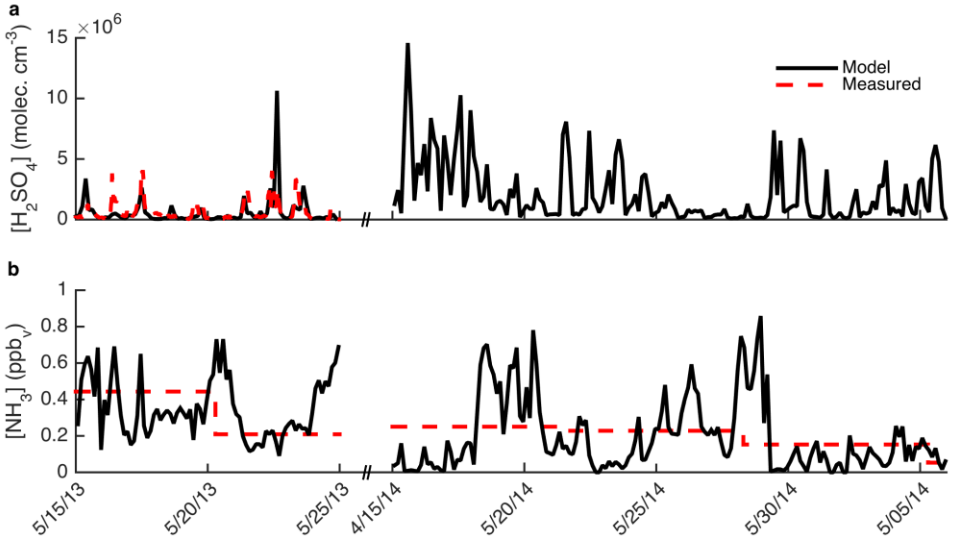

Supplementary Figure 10. Panel **a** shows the measured and modelled H_2SO_4 concentration at SMEAR II during the periods 15th of May to 24th of May 2013 and 15th of April to 5th of May 2014. The sulfuric acid concentration was measured with the CI-APi-TOF that also measured the HOM concentrations. Panel **b** shows the measured and modelled ammonia concentrations during the periods 15th of May to 24th of May 2013 and 15th of April to 5th of May 2014. The modelled ammonia results are from SMEAR II while the ammonia measurements were performed with a filter-based technique with one week resolution at the European Monitoring and Evaluation Programme (EMEP) station Ähtäri II, 85 km north from SMEAR II. The average measured and modelled H_2SO_4 concentrations during the 2013 period were 5.4×10^5 molecules cm^{-3} and 6.1×10^5 molecules cm^{-3} respectively. For the spring 2014 period the modelled average H_2SO_4 concentration was 2.1×10^6 molecules cm^{-3}. The average measured NH_3 concentrations for the 2013 and 2014 spring period were 0.35 and 0.20 ppb$_v$ respectively. The modelled NH_3 concentrations for the 2013 and 2014 spring period were 0.33 and 0.19 ppb$_v$ respectively.

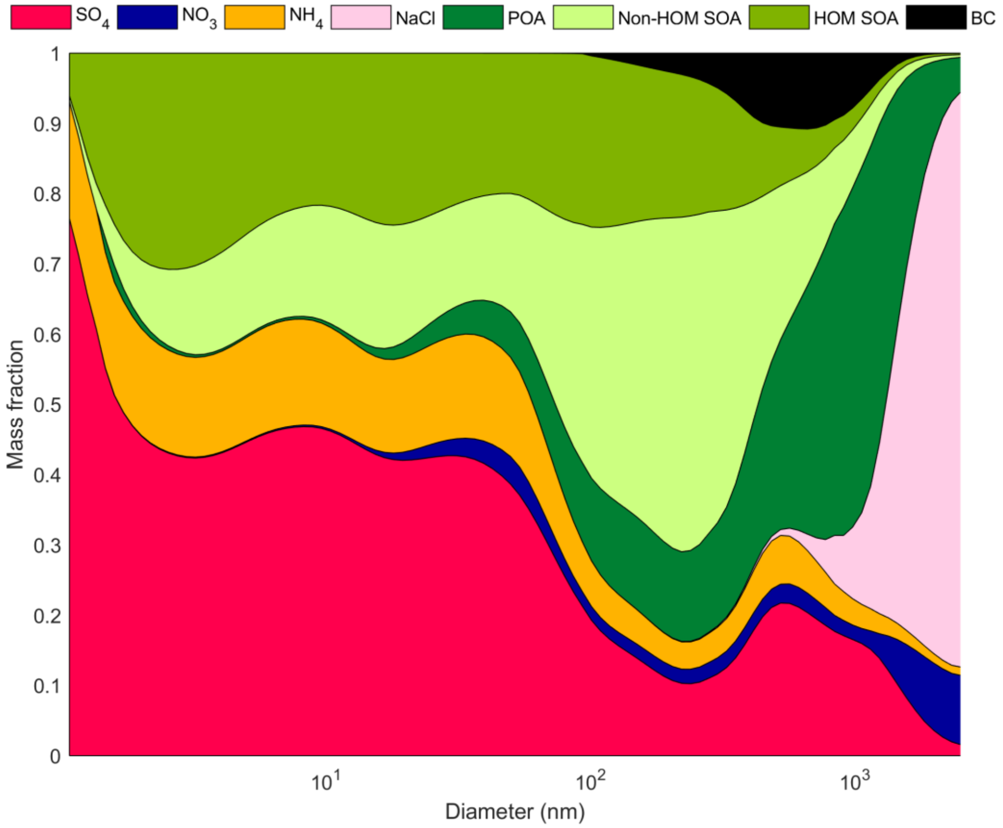

Supplementary Figure 11. Modelled size resolved chemical composition of particles in the diameter size range 1.07 nm to 2.5 µm ($PM_{2.5}$) for the periods 15th to 24th of May, 2013 and 15th of April to 5th of May, 2014.

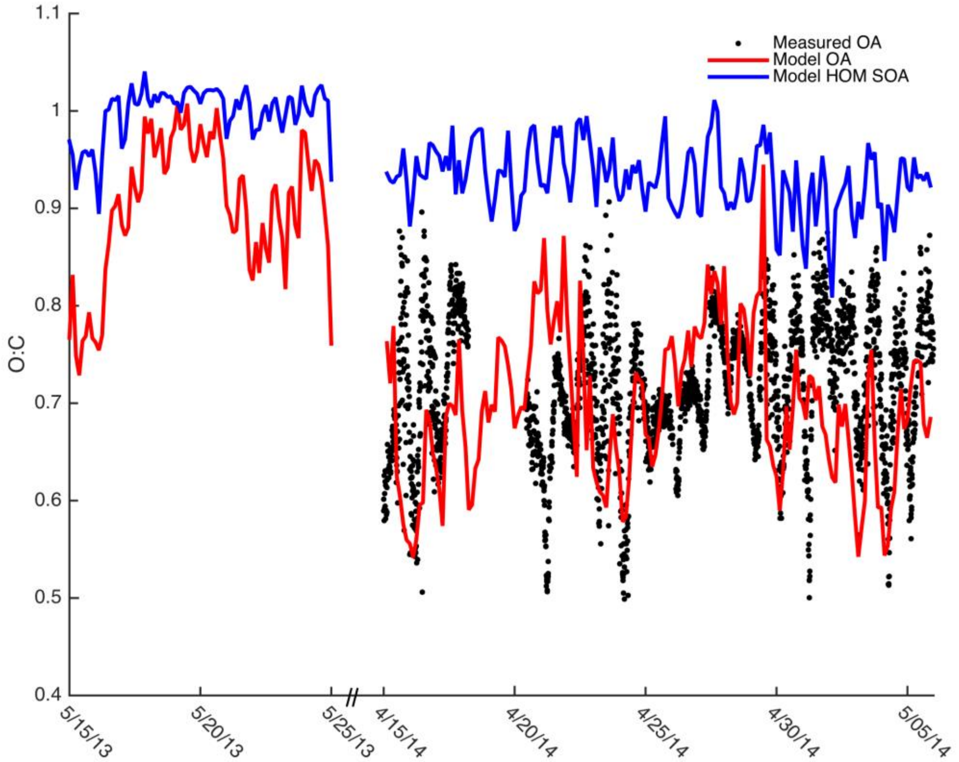

Supplementary Figure 12. Modelled and measured (from the AMS) O:C of the PM_1 OA mass at SMEAR II. The O:C for the HOM SOA is also shown. During spring 2014 the measured and modelled average O:C are 0.72 and 0.70 respectively. The O:C of the modelled HOM SOA are on average 0.93 during spring 2014 and 1.00 during spring 2013.

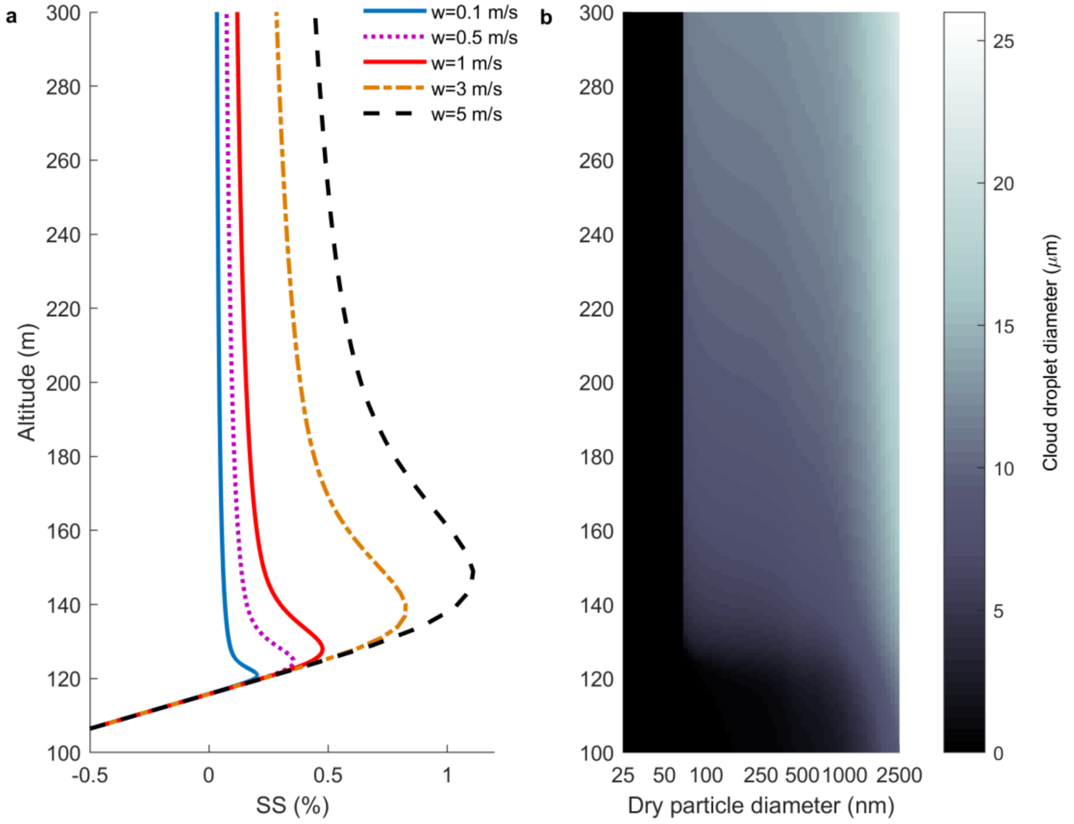

Supplementary Figure 13. Example with results from the adiabatic cloud parcel model. Panel **a** shows the modelled cloud water vapour supersaturation (SS) as a function of the altitude for the updraft velocities (*w*): 0.1, 0.5, 1, 3 and 5 m/s. The maximum SS (SS_{max}) is reached within a few to a few 10s of meters from the cloud base at an altitude of ~115 m. All cloud parcels were assumed to start at the ground (0 m) with an initial relative humidity of 95 % (SS = -5.0 %). Panel **b** illustrates how the cloud droplet diameters changes as a function of altitude for a simulation with *w*=1 m/s (red curve in panel **a**). At this updraft velocity all particles with an initial dry particle diameter larger than 68 nm are activated and grow into cloud droplets. In this example we used the modelled aerosol particle properties from the surface layer at SMEAR II, from 8 pm on the 24th of April 2014, as input to the adiabatic cloud parcel model.

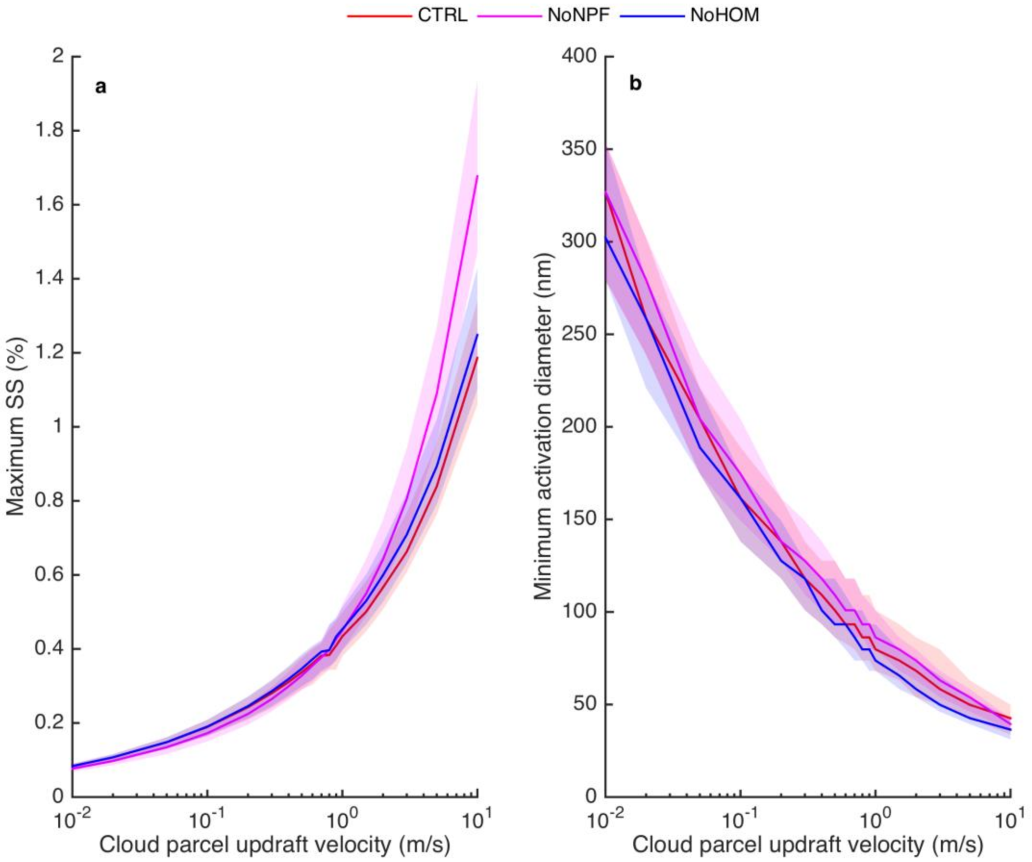

Supplementary Figure 14. Panel (**a**) shows the maximum cloud water vapour supersaturation (SS) and (**b**) minimum dry particle diameter of the particles that are activated into cloud droplets as a function of the adiabatic cloud parcel model updraft velocity. The curves illustrates the median results from the CTRL, NoNPF and NoHOM simulations that cover conditions during the periods May 15-24, 2013 and April 15 to May 5, 2014. The shaded areas shows the data span between the 25th and 75th percentiles.

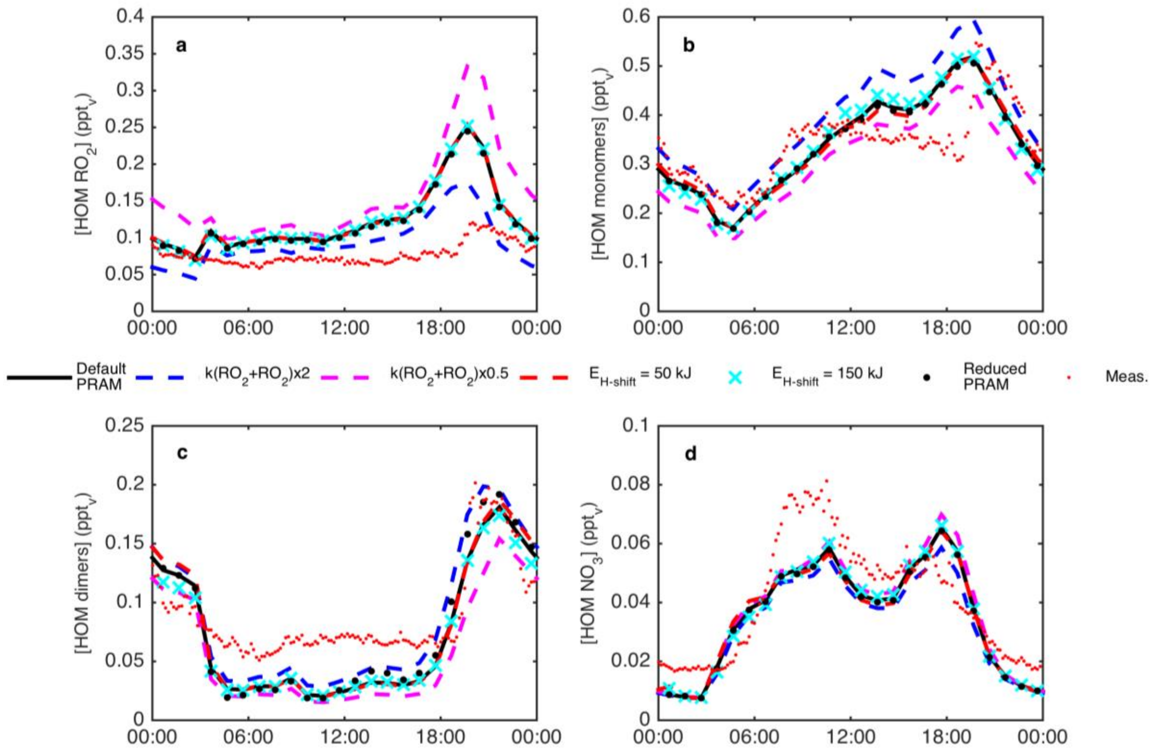

Supplementary Figure 15. Modelled and measured HOM(g) concentrations at SMEAR II between 15th and 24th of May, 2013. Panel **a** shows the concentrations of HOM peroxy radicals (RO_2), **b** HOM closed shell monomers without nitrate functional groups, **c** HOM dimers, and **d** closed shell HOM organonitrate monomers (HOM-NO_3). The inset plots in each panel shows the mean diurnal concentration trends of each HOM species type. The different model results are from the PRAM model sensitivity tests where the RO_2 + RO_2 reaction rates (k(RO_2 + RO_2)) were scaled up or down with a factor of two compared to the default values (Supplementary Table 4) or when the RO_2 H-shift activation energies ($E_{H\text{-}shift}$) were scaled up or down with 50 kJ from the default value of 100 kJ. Shown are also the results from a model simulation using the reduced PRAM version without explicit representation of individual dimer HOM species (Supplementary Table 7).

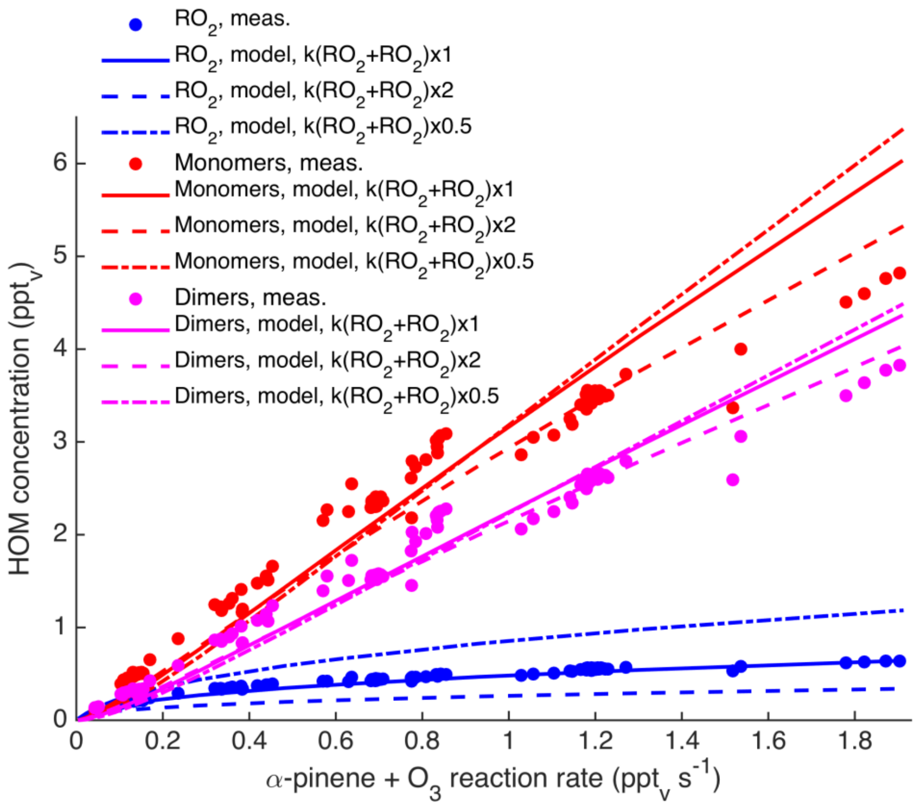

Supplementary Figure 16. Modelled and measured HOM(g) concentrations during a JPAC α-pinene ozonolysis experiment[1]. The different model results are from the PRAM model sensitivity tests where all RO_2 + RO_2 reaction rates (k(RO_2 + RO_2)) were scaled up or down with a factor of two compared to the default values (Supplementary Table 4).

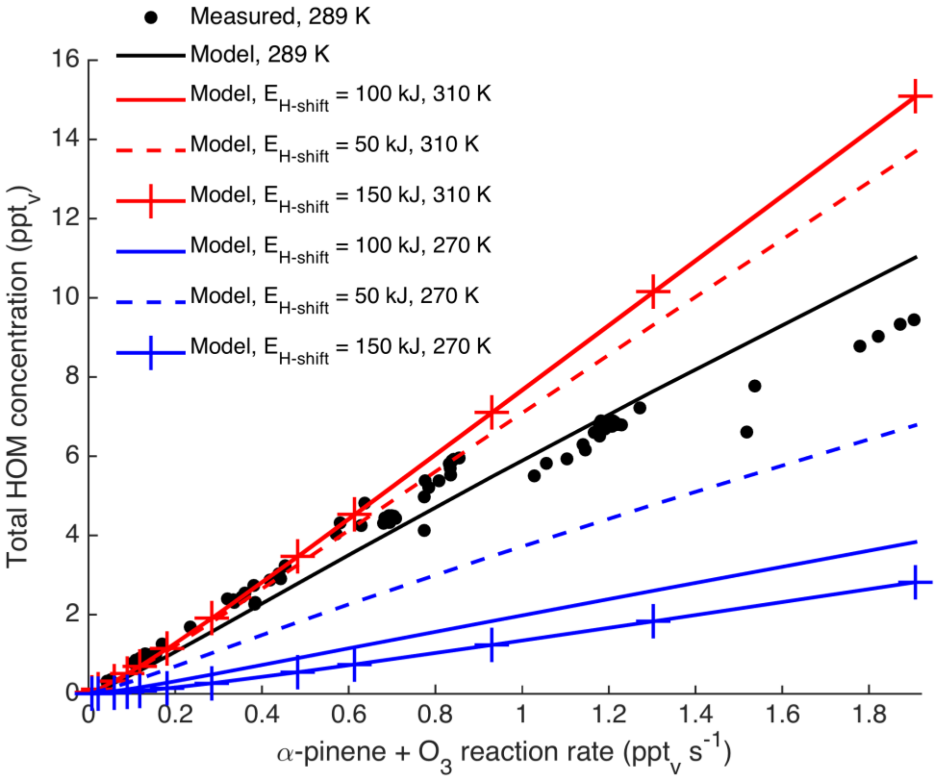

Supplementary Figure 17. Modelled and measured HOM(g) concentrations during a JPAC α-pinene ozonolysis experiment[1]. The different model results are from the PRAM model sensitivity tests with different RO_2 H-shift activation energies ($E_{H\text{-}shift}$). At the reference temperature 289 K, the RO_2 autoxidation reaction rates and the model results are identical for the different PRAM model setups.

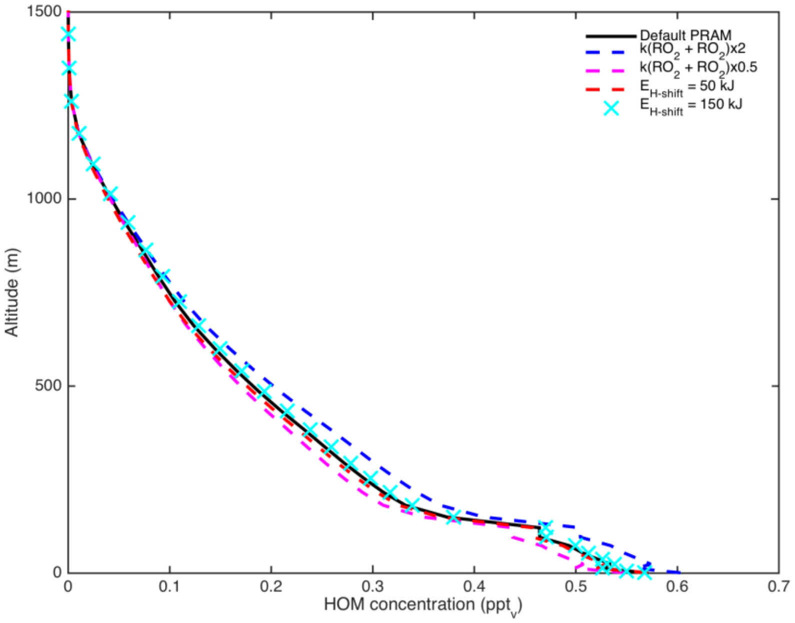

Supplementary Figure 18. Modelled average vertical HOM concentration profiles at SMEAR II between 15th and 24th of May, 2013. The results are from the different model sensitivity tests where the $RO_2 + RO_2$ reaction rates ($k(RO_2 + RO_2)$) were scaled up or down with a factor of two compared to the default values (Supplementary Table 4) or when the RO_2 H-shift activation energies ($E_{H\text{-}shift}$) were scaled up or down with 50 kJ from the default value of 100 kJ.

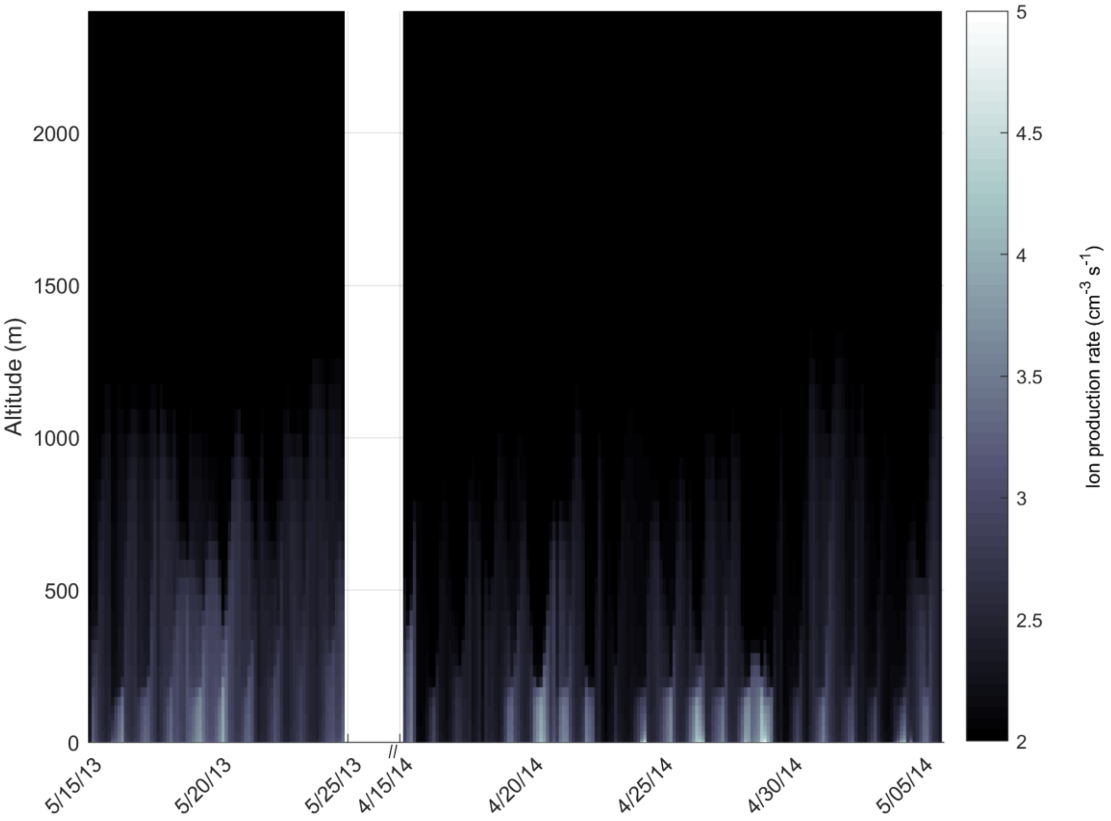

Supplementary Figure 19. Modelled ion production rate at the SMEAR II station caused by galactic cosmic rays and the decay of radon. The ion production rates were used as input to the atmospheric cluster dynamics code (ACDC).

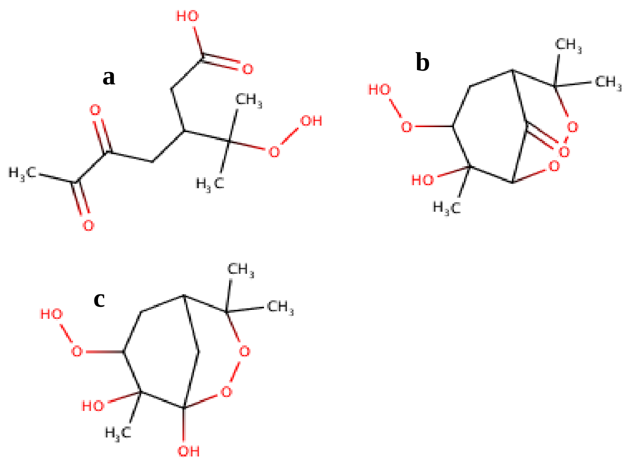

Supplementary Figure 20. Molecular structures of the PRAM HOM species **a** C10H16O6iso1, **b** C10H16O6iso2 and **c** C10H18O6.

Supplementary Tables

Supplementary Table 1. Yield of formation of the initial RO_2 species that undergo autoxidation in PRAM[1]

Monoterpene	Oxidant	Yield of RO_2 that initiates the autoxidation.
α-pinene	O_3	In total the theoretical maximum HOM yield become 9 % during no NO_x conditions.
β-pinene	O_3	Not considered in this work, ≤0.1 % according to smog chamber and flow tube experiments[2,3]
Limonene	O_3	21.9 %, Comparable with the final HOM yield of 17 % estimated by Ehn et al. [2]
Carene	O_3	Same as α-pinene
α-pinene	OH	2.5 % after the initial α-pinene + OH. Comparable with the 2.4 % HOM yield from Berndt et al. [4]
β-pinene	OH	1.0 % after the initial α-pinene + OH. Jokinen et al. estimated a HOM molar yield of 0.58 %[3]
Limonene	OH	1.0 % after the initial α-pinene + OH. Jokinen et al. estimated a HOM molar yield of 0.93 %[3]
Carene	OH	Same as α-pinene

Supplementary Table 2a. List of PRAM[1] species formed when monoterpenes are oxidized by ozone. The table includes the species name in PRAM, molecular formula, compound type, functional groups used when estimating their pure liquid saturation vapour pressure (p_0) using SIMPOL[5] and their p_0 at 298 K. The peroxy and alkoxy radical functional groups are not listed and were not considered when we calculated p_0.

PRAM name	Molecular formula	Type	Alcohol	Aldehyde	Ketone	Nitrate	Endoperoxide	Hydroperoxide	Peroxy acid	Carboxylic acid	log_{10} (p_0 (Pa)) at 298 K
C10H15O2O2	$C_{10}H_{15}O_4$	RO_2		1	1						0.35
C10H15O3O2	$C_{10}H_{15}O_5$	RO_2		1	2						0.59
C10H15O4O2	$C_{10}H_{15}O_6$	RO_2		1	1			1			-2.1
C10H15O5O2	$C_{10}H_{15}O_7$	RO_2		1	2			1			-3.0
C10H15O6O2	$C_{10}H_{15}O_8$	RO_2			1			1	1		-3.2
C10H15O7O2	$C_{10}H_{15}O_9$	RO_2			2			1	1		-4.1
C10H15O8O2	$C_{10}H_{15}O_{10}$	RO_2			1			2	1		-5.6
C10H15O9O2	$C_{10}H_{15}O_{11}$	RO_2			2			2	1		-6.6
C10H15O10O2	$C_{10}H_{15}O_{12}$	RO_2			1			3	1		-8.1
C10H15O11O2	$C_{10}H_{15}O_{13}$	RO_2			2			3	1		-9.0
C10H15O12O2	$C_{10}H_{15}O_{14}$	RO_2						3	2		-9.6
C10H15O2O	$C_{10}H_{15}O_3$	RO		1	1						0.35
C10H15O3O	$C_{10}H_{15}O_4$	RO		1	2						0.59
C10H15O4O	$C_{10}H_{15}O_5$	RO		1	1			1			-2.1
C10H15O5O	$C_{10}H_{15}O_6$	RO		1	2			1			-3.0
C10H15O6O	$C_{10}H_{15}O_7$	RO			1			1	1		-3.2
C10H15O7O	$C_{10}H_{15}O_8$	RO			2			1	1		-4.1
C10H15O8O	$C_{10}H_{15}O_9$	RO			1			2	1		-5.6
C10H15O9O	$C_{10}H_{15}O_{10}$	RO			2			2	1		-6.6
C10H15O10O	$C_{10}H_{15}O_{11}$	RO			1			3	1		-8.1
C10H15O11O	$C_{10}H_{15}O_{12}$	RO			2			3	1		-9.0
C10H15O12O	$C_{10}H_{15}O_{13}$	RO						3	2		-9.6
C10H14O3	$C_{10}H_{14}O_3$	RCHO		2	1						-0.97
C10H14O4	$C_{10}H_{14}O_4$	RCHO		1	3						-1.5
C10H14O5	$C_{10}H_{14}O_5$	RCHO		1	2			1			-3.0
C10H14O6	$C_{10}H_{14}O_6$	RCHO		1	3			1			-4.0
C10H14O7	$C_{10}H_{14}O_7$	RCHO			2			1	1		-4.1
C10H14O8	$C_{10}H_{14}O_8$	RCHO			3			1	1		-5.1
C10H14O9	$C_{10}H_{14}O_9$	RCHO			2			2	1		-6.6
C10H14O10	$C_{10}H_{14}O_{10}$	RCHO			3			2	1		-7.5
C10H14O11	$C_{10}H_{14}O_{11}$	RCHO			2			3	1		-9.0

C10H14O12	$C_{10}H_{14}O_{12}$	RCHO			3			3	1		-10.0
C10H14O13	$C_{10}H_{14}O_{13}$	RCHO			1			3	2		-10.5
C10H16O3	$C_{10}H_{16}O_3$	ROH	1	1	1						-1.8
C10H16O4iso1	$C_{10}H_{16}O_4$	RCOOH	1		1					1	-4.0
C10H16O5iso1	$C_{10}H_{16}O_5$	RCOOH			1			1		1	-4.3
C10H16O6iso1	$C_{10}H_{16}O_6$	RCOOH or ROOH			2				1	1	-5.2
C10H16O7iso1	$C_{10}H_{16}O_7$	ROOH or ROH	1		1			1	1		-5.4
C10H16O8iso1	$C_{10}H_{16}O_8$	ROOH or ROH	1		2			1	1		-6.3
C10H16O9iso1	$C_{10}H_{16}O_9$	ROOH or ROH	1		1			2	1		-7.8
C10H16O10	$C_{10}H_{16}O_{10}$	ROOH or ROH	1		2			2	1		-8.8
C10H16O11	$C_{10}H_{16}O_{11}$	ROOH or ROH	1		1			3	1		-10.3
C10H16O12	$C_{10}H_{16}O_{12}$	ROOH or ROH	1		2			3	1		-11.2
C10H16O13	$C_{10}H_{16}O_{13}$	ROOH or ROH	1					3	2		-11.8
C10H16O14	$C_{10}H_{16}O_{14}$	ROOH						4	2		-12.0
C10H15O2NO3	$C_{10}H_{15}NO_5$	R-O-NO_2		1	1	1					-1.8
C10H15O3NO3	$C_{10}H_{15}NO_6$	R-O-NO_2		1	2	1					-2.8
C10H15O4NO3	$C_{10}H_{15}NO_7$	R-O-NO_2		1	1	1			1		-4.3
C10H15O5NO3	$C_{10}H_{15}NO_8$	R-O-NO_2		1	2	1		1			-5.2
C10H15O6NO3	$C_{10}H_{15}NO_9$	R-O-NO_2			1	1		1	1		-5.4
C10H15O7NO3	$C_{10}H_{15}NO_{10}$	R-O-NO_2			2	1		1	1		-6.3
C10H15O8NO3	$C_{10}H_{15}NO_{11}$	R-O-NO_2			1	1		2	1		-7.8
C10H15O9NO3	$C_{10}H_{15}NO_{12}$	R-O-NO_2			2	1		2	1		-8.8
C10H15O10NO3	$C_{10}H_{15}NO_{13}$	R-O-NO_2			1	1		3	1		-10.3
C10H15O11NO3	$C_{10}H_{15}NO_{14}$	R-O-NO_2			2	1		3	1		-11.2
C10H15O12NO3	$C_{10}H_{15}NO_{15}$	R-O-NO_2				1		3	2		-11.8
C20H30O5	$C_{20}H_{30}O_5$	ROOR	1	1	1		1				-6.5
C20H30O6	$C_{20}H_{30}O_6$	ROOR		2	2		1				-6.6
C20H30O7	$C_{20}H_{30}O_7$	ROOR	1	1	3		1				-8.4
C20H30O8	$C_{20}H_{30}O_8$	ROOR		1	3		1	1			-8.6
C20H30O9	$C_{20}H_{30}O_9$	ROOR	1	1	2		1		1		-9.8
C20H30O10	$C_{20}H_{30}O_{10}$	ROOR		1	2		1	1	1		-10.1
C20H30O11	$C_{20}H_{30}O_{11}$	ROOR	1	1	2		1	1	1		-12.3
C20H30O12	$C_{20}H_{30}O_{12}$	ROOR		1	2		1	2	1		-12.5
C20H30O13	$C_{20}H_{30}O_{13}$	ROOR	1	1	2		1	2	1		-14.7
C20H30O14	$C_{20}H_{30}O_{14}$	ROOR		1	2		1	3	1		-15.0
C20H30O15	$C_{20}H_{30}O_{15}$	ROOR	1	1	2		1	2	2		-17.2
C20H30O16	$C_{20}H_{30}O_{16}$	ROOR		1	1		1	3	2		-16.5
C20H30O17	$C_{20}H_{30}O_{17}$	ROOR	1	1	1		1	3	2		-18.7
C20H31O4NO3	$C_{20}H_{31}NO_7$	ROOR-O-NO_2		1	1	1	1				-6.5
C20H31O5NO3	$C_{20}H_{31}NO_8$	ROOR-O-NO_2		1	2	1	1				-7.4
C20H31O6NO3	$C_{20}H_{31}NO_9$	ROOR-O-NO_2		1	1	1	1	1			-8.9
C20H31O7NO3	$C_{20}H_{31}NO_{10}$	ROOR-O-NO_2	1	1	1	1	1	1			-11.1
C20H31O8NO3	$C_{20}H_{31}NO_{11}$	ROOR-O-NO_2			1	1	1	1	1		-10.0

C20H31O9NO3	$C_{20}H_{31}NO_{12}$	ROOR-O-NO_2	1		1	1	1	1	1	-12.2
C20H31O10NO3	$C_{20}H_{31}NO_{13}$	ROOR-O-NO_2			1	1	1	2	1	-12.5
C20H31O11NO3	$C_{20}H_{31}NO_{14}$	ROOR-O-NO_2	1		1	1	1	2	1	-14.7
C20H31O12NO3	$C_{20}H_{31}NO_{15}$	ROOR-O-NO_2			1	1	1	3	1	-14.9
C20H31O13NO3	$C_{20}H_{31}NO_{16}$	ROOR-O-NO_2			1	1	1	2	2	-14.9
C20H31O14NO3	$C_{20}H_{31}NO_{17}$	ROOR-O-NO_2				1	1	3	2	-16.4
C20H31O15NO3	$C_{20}H_{31}NO_{18}$	ROOR-O-NO_2			1	1	1	3	2	-17.3
C19H28O5	$C_{19}H_{28}O_5$	ROOR	1	1	1		1			-6.1
C19H28O6	$C_{19}H_{28}O_6$	ROOR		2	2		1			-6.1
C19H28O7	$C_{19}H_{28}O_7$	ROOR	1	1	3		1			-7.9
C19H28O8	$C_{19}H_{28}O_8$	ROOR		1	3		1	1		-8.2
C19H28O9	$C_{19}H_{28}O_9$	ROOR	1	1	2		1		1	-9.4
C19H28O10	$C_{19}H_{28}O_{10}$	ROOR		1	2		1	1	1	-9.7
C19H28O11	$C_{19}H_{28}O_{11}$	ROOR	1	1	2		1	1	1	-11.9
C19H28O12	$C_{19}H_{28}O_{12}$	ROOR		1	2		1	2	1	-12.1
C19H28O13	$C_{19}H_{28}O_{13}$	ROOR	1	1	2		1	2	1	-14.3
C19H28O14	$C_{19}H_{28}O_{14}$	ROOR		1	2		1	3	1	-14.6
C19H28O15	$C_{19}H_{28}O_{15}$	ROOR	1	1	2		1	2	2	-16.7
C19H28O16	$C_{19}H_{28}O_{16}$	ROOR		1	1		1	3	2	-16.0
C19H28O17	$C_{19}H_{28}O_{17}$	ROOR	1	1	1		1	3	2	-18.2
C19H28O18	$C_{19}H_{28}O_{18}$	ROOR			1		1	3	3	-17.2
C19H29O5NO3	$C_{19}H_{29}NO_8$	ROOR-O-NO_2		1	2	1	1			-7.0
C19H29O6NO3	$C_{19}H_{29}NO_9$	ROOR-O-NO_2		1	1	1	1	1		-8.5
C19H29O7NO3	$C_{19}H_{29}NO_{10}$	ROOR-O-NO_2	1	1	1	1	1	1		-10.7
C19H29O8NO3	$C_{19}H_{29}NO_{11}$	ROOR-O-NO_2			1	1	1	1	1	-9.6
C19H29O9NO3	$C_{19}H_{29}NO_{12}$	ROOR-O-NO_2	1		1	1	1	1	1	-11.8
C19H29O10NO3	$C_{19}H_{29}NO_{13}$	ROOR-O-NO_2			1	1	1	2	1	-12.0
C19H29O11NO3	$C_{19}H_{29}NO_{14}$	ROOR-O-NO_2	1		1	1	1	2	1	-14.2
C19H29O12NO3	$C_{19}H_{29}NO_{15}$	ROOR-O-NO_2			1	1	1	3	1	-14.5
C19H29O13NO3	$C_{19}H_{29}NO_{16}$	ROOR-O-NO_2			1	1	1	2	2	-14.5
C19H29O14NO3	$C_{19}H_{29}NO_{17}$	ROOR-O-NO_2				1	1	3	2	-16.0
C19H29O15NO3	$C_{19}H_{29}NO_{18}$	ROOR-O-NO_2			1	1	1	3	2	-16.9
C18H26O4	$C_{18}H_{26}O_4$	ROOR		1	1		1			-3.4
C18H26O5	$C_{18}H_{26}O_5$	ROOR	1	1	1		1			-5.6
C18H26O6	$C_{18}H_{26}O_6$	ROOR		2	2		1			-5.7
C18H26O7	$C_{18}H_{26}O_7$	ROOR	1	1	3		1			-7.5
C18H26O8	$C_{18}H_{26}O_8$	ROOR		1	3		1	1		-7.8
C18H26O9	$C_{18}H_6O_9$	ROOR	1	1	2		1		1	-9.0
C18H26O10	$C_{18}H_{26}O_{10}$	ROOR		1	2		1	1	1	-9.2
C18H26O11	$C_{18}H_{26}O_{11}$	ROOR	1	1	2		1	1	1	-11.4
C18H26O12	$C_{18}H_{26}O_{12}$	ROOR		1	2		1	2	1	-11.7
C18H26O13	$C_{18}H_{26}O_{13}$	ROOR	1	1	2		1	2	1	-13.9

C18H26O14	$C_{18}H_{26}O_{14}$	ROOR		1	2		1	3	1	-14.1
C18H26O15	$C_{18}H_{26}O_{15}$	ROOR	1	1	2		1	2	2	-16.3
C18H26O16	$C_{18}H_{26}O_{16}$	ROOR		1	1		1	3	2	-15.6
C18H26O17	$C_{18}H_{26}O_{17}$	ROOR	1	1	1		1	3	2	-17.8
C18H26O18	$C_{18}H_{26}O_{18}$	ROOR			1		1	3	3	-16.7
C18H27O6NO3	$C_{18}H_{27}NO_9$	ROOR-O-NO_2		1	1	1	1	1		-8.1
C18H27O7NO3	$C_{18}H_{27}NO_{10}$	ROOR-O-NO_2	1	1	1	1	1	1		-10.3
C18H27O8NO3	$C_{18}H_{27}NO_{11}$	ROOR-O-NO_2			1	1	1	1	1	-9.2
C18H27O9NO3	$C_{18}H_{27}NO_{12}$	ROOR-O-NO_2	1		1	1	1	1	1	-11.4
C18H27O10NO3	$C_{18}H_{27}NO_{13}$	ROOR-O-NO_2			1	1	1	2	1	-11.6
C18H27O11NO3	$C_{18}H_{27}NO_{14}$	ROOR-O-NO_2	1		1	1	1	2	1	-13.8
C18H27O12NO3	$C_{18}H_{27}NO_{15}$	ROOR-O-NO_2			1	1	1	3	1	-14.1
C18H27O13NO3	$C_{18}H_{27}NO_{16}$	ROOR-O-NO_2			1	1	1	2	2	-14.0
C18H27O14NO3	$C_{18}H_{27}NO_{17}$	ROOR-O-NO_2				1	1	3	2	-15.6
C18H27O15NO3	$C_{18}H_{27}NO_{18}$	ROOR-O-NO_2			1	1	1	3	2	-16.5
C18H27O16NO3	$C_{18}H_{27}NO_{19}$	ROOR-O-NO_2				1	1	4	2	-18.0

Supplementary Table 2b. List of PRAM[1] species formed when monoterpenes are oxidized by OH. The table includes the species name in PRAM, molecular formula, compound type, functional groups used when estimating their pure liquid saturation vapor pressure (p_0) using SIMPOL[5] and their p_0 at 298 K. The peroxy and alkoxy radical functional groups are not listed and were not considered when we calculated p_0.

PRAM name	Molecular formula	Type	Alcohol	Ketone	Nitrate	Endoperoxide	Hydroperoxide	C=C bond	Non-aromatic ring	log_{10} (p_0 (Pa)) at 298 K
C10H17O3O2	$C_{10}H_{17}O_5$	RO_2	1				1	1	1	-2.2
C10H17O4O2	$C_{10}H_{17}O_6$	RO_2	2				1		1	-4.2
C10H17O5O2	$C_{10}H_{17}O_7$	RO_2	1			1	1		1	-2.4
C10H17O6O2	$C_{10}H_{17}O_8$	RO_2	2			1	1		1	-4.6
C10H17O7O2	$C_{10}H_{17}O_9$	RO_2	1			1	2		1	-4.9
C10H17O8O2	$C_{10}H_{17}O_{10}$	RO_2	2			1	2		1	-7.1
C10H17O3O	$C_{10}H_{17}O_4$	RO	1				1	1	1	-2.2
C10H17O4O	$C_{10}H_{17}O_5$	RO	2				1		1	-4.2
C10H17O5O	$C_{10}H_{17}O_6$	RO	1			1	1		1	-2.4
C10H17O6O	$C_{10}H_{17}O_7$	RO	2			1	1		1	-4.6
C10H17O7O	$C_{10}H_{17}O_8$	RO	1			1	2		1	-4.9
C10H16O4iso2	$C_{10}H_{16}O_4$	RCHO	1	1			1	1	1	-3.1
C10H16O5iso2	$C_{10}H_{16}O_5$	RCHO	2	1			1		1	-5.2
C10H16O6iso2	$C_{10}H_{16}O_6$	RCHO	1	1		1	1		1	-3.4
C10H16O7iso2	$C_{10}H_{16}O_7$	RCHO	2	1		1	1		1	-5.6
C10H16O8iso2	$C_{10}H_{16}O_8$	RCHO	1	1		1	2		1	-5.8
C10H16O9iso2	$C_{10}H_{16}O_9$	RCHO	2	1		1	2		1	-8.0
C10H18O4	$C_{10}H_{18}O_4$	ROOH or ROH	2				1	1	1	-4.3
C10H18O5	$C_{10}H_{18}O_5$	ROOH or ROH	3				1		1	-6.4
C10H18O6	$C_{10}H_{18}O_6$	ROOH or ROH	2			1	1		1	-4.6

C10H18O7	$C_{10}H_{18}O_7$	ROOH or ROH	3			1	1		1	-6.8
C10H18O8	$C_{10}H_{18}O_8$	ROOH or ROH	2			1	2		1	-7.1
C10H18O9	$C_{10}H_{18}O_9$	ROOH or ROH	1			1	3		1	-7.4
C10H18O10	$C_{10}H_{18}O_{10}$	ROOH or ROH	2			1	3		1	-9.6
C10H17O3NO3	$C_{10}H_{17}NO_6$	R-O-NO_2	1		1		1	1	1	-4.2
C10H17O4NO3	$C_{10}H_{17}NO_7$	R-O-NO_2	2		1		1		1	-6.4
C10H17O5NO3	$C_{10}H_{17}NO_8$	R-O-NO_2	1		1	1	1		1	-4.6
C10H17O6NO3	$C_{10}H_{17}NO_9$	R-O-NO_2	2		1	1	1		1	-6.8
C10H17O7NO3	$C_{10}H_{17}NO_{10}$	R-O-NO_2	1		1	1	2		1	-7.1
C10H17O8NO3	$C_{10}H_{17}NO_{11}$	R-O-NO_2	2		1	1	2		1	-9.2
C20H34O6	$C_{20}H_{34}O_6$	ROOR	1	1		1	1		2	-7.7
C20H34O7	$C_{20}H_{34}O_7$	ROOR	1	2		1	1		1	-8.6
C20H34O8	$C_{20}H_{34}O_8$	ROOR	1	1		2	1		1	-8.0
C20H34O9	$C_{20}H_{34}O_9$	ROOR	2	1		2	1		1	-10.2
C20H34O10	$C_{20}H_{34}O_{10}$	ROOR	1	1		2	2		1	-10.5
C20H34O11	$C_{20}H_{34}O_{11}$	ROOR	2	1		2	2		1	-12.6
C20H34O12	$C_{20}H_{34}O_{12}$	ROOR	3	1		2	2		1	-14.8
C20H34O13	$C_{20}H_{34}O_{13}$	ROOR	2	1		2	3		1	-15.1
C20H35O5NO3	$C_{20}H_{35}NO_8$	ROOR-O-NO_2		1	1	1	1		1	-7.6
C20H35O6NO3	$C_{20}H_{35}NO_9$	ROOR-O-NO_2	1	1	1	1	1		1	-9.8
C20H35O7NO3	$C_{20}H_{35}NO_{10}$	ROOR-O-NO_2	1	2	1	1	1		1	-10.7
C20H35O8NO3	$C_{20}H_{35}NO_{11}$	ROOR-O-NO_2	1	1	1	2	1		1	-10.2
C20H35O9NO3	$C_{20}H_{35}NO_{12}$	ROOR-O-NO_2	2	1	1	2	1		1	-12.4
C20H35O10NO3	$C_{20}H_{35}NO_{13}$	ROOR-O-NO_2	1	1	1	2	2		1	-12.6
C20H35O11NO3	$C_{20}H_{35}NO_{14}$	ROOR-O-NO_2	2	1	1	2	2		1	-14.8
C19H32O6	$C_{19}H_{32}O_6$	ROOR	1	1		1	1		2	-7.3
C19H32O7	$C_{19}H_{32}O_7$	ROOR	1	2		1	1		1	-8.1
C19H32O8	$C_{19}H_{32}O_8$	ROOR	1	1		2	1		1	-7.6
C19H32O9	$C_{19}H_{32}O_9$	ROOR	2	1		2	1		1	-9.8
C19H32O10	$C_{19}H_{32}O_{10}$	ROOR	1	1		2	2		1	-10.0
C19H32O11	$C_{19}H_{32}O_{11}$	ROOR	2	1		2	2		1	-12.2
C19H32O12	$C_{19}H_{32}O_{12}$	ROOR	3	1		2	2		1	-14.4
C19H32O13	$C_{19}H_{32}O_{13}$	ROOR	2	1		2	3		1	-14.7
C19H32O14	$C_{19}H_{32}O_{14}$	ROOR	3	1		2	3		1	-16.8
C19H33O6NO3	$C_{19}H_{33}NO_9$	ROOR-O-NO_2	1	1	1	1	1		1	-9.4
C19H33O7NO3	$C_{19}H_{33}NO_{10}$	ROOR-O-NO_2	1	2	1	1	1		1	-10.3
C19H33O8NO3	$C_{19}H_{33}NO_{11}$	ROOR-O-NO_2	1	1	1	2	1		1	-9.8
C19H33O9NO3	$C_{19}H_{33}NO_{12}$	ROOR-O-NO_2	2	1	1	2	1		1	-12.0
C19H33O10NO3	$C_{19}H_{33}NO_{13}$	ROOR-O-NO_2	1	1	1	2	2		1	-12.2
C19H33O11NO3	$C_{19}H_{33}NO_{14}$	ROOR-O-NO_2	2	1	1	2	2		1	-14.4
C18H30O5	$C_{18}H_{30}O_5$	ROOR	1			1	1		2	-5.9
C18H30O6	$C_{18}H_{30}O_6$	ROOR	1	1		1	1		2	-6.9

C18H30O7	$C_{18}H_{30}O_7$	ROOR	1	2		1	1		1	-7.7
C18H30O8	$C_{18}H_{30}O_8$	ROOR	1	1		2	1		1	-7.2
C18H30O9	$C_{18}H_{30}O_9$	ROOR	2	1		2	1		1	-9.3
C18H30O10	$C_{18}H_{30}O_{10}$	ROOR	1	1		2	2		1	-9.6
C18H30O11	$C_{18}H_{30}O_{11}$	ROOR	2	1		2	2		1	-11.8
C18H30O12	$C_{18}H_{30}O_{12}$	ROOR	3	1		2	2		1	-14.0
C18H30O13	$C_{18}H_{30}O_{13}$	ROOR	2	1		2	3		1	-14.2
C18H30O14	$C_{18}H_{30}O_{14}$	ROOR	3	1		2	3		1	-16.4
C18H31O7NO3	$C_{18}H_{31}NO_{10}$	ROOR-O-NO_2	1	2	1	1	1		1	-9.9
C18H31O8NO3	$C_{18}H_{31}NO_{11}$	ROOR-O-NO_2	1	1	1	2	1		1	-9.3
C18H31O9NO3	$C_{18}H_{31}NO_{12}$	ROOR-O-NO_2	2	1	1	2	1		1	-11.5
C18H31O10NO3	$C_{18}H_{31}NO_{13}$	ROOR-O-NO_2	1	1	1	2	2		1	-11.8
C18H31O11NO3	$C_{18}H_{31}NO_{14}$	ROOR-O-NO_2	2	1	1	2	2		1	-14.0
C18H31O12NO3	$C_{18}H_{31}NO_{15}$	ROOR-O-NO_2	1	1	1	2	3		1	-14.2

Supplementary Table 3. List of species from MCMv3.3.1 which has a specific role in the PRAM mechanism.

Name	Role in PRAM
NO	React with the autoxidation RO_2 species and form RO, R-O-NO_2, RCHO species or causes fragmentation of the C10 RO_2 species.
O_3	React with monoterpenes containing endocyclic double bonds and form the Criegiee intermediates (CI) APINOOA and LIMOOA that decomposes and form the RO_2 (C10H15O2O2) that initializes the autoxidation mechanism.
HO_2	React with the autoxidation RO_2 species and form ROOH.
OH	React with the monoterpenes and form RO_2 species that can undergo autoxidation. Is also formed when the CI from the ozonolysis of monoterpenes is decomposing and form RO_2 species that initialize the autoxidation chain, and upon unimolecular termination of the RO_2 in PRAM.
α-pinene	Small branching ratio of the RO_2 formed during the OH oxidation of α-pinene are assumed to undergo autoxidation.
β-pinene	Small branching ratio of the RO_2 formed during the OH oxidation of β-pinene are assumed to undergo autoxidation.
Limonene	Small branching ratio of the RO_2 formed during the OH oxidation of limonene are assumed to undergo autoxidation.
Carene	After the initial oxidation with O_3 carene is assumed to form the same CI as α-pinene and thus form HOM with identical yields and composition as α-pinene.
APINOOA	CI formed from the α-pinene + O_3 reaction. Is also assumed to be formed from the carene + O_3 reaction. This CI is assumed to decompose and form the initial RO_2 that initializes the autoxidation mechanism.
LIMOOA	CI formed from the limonene + O_3 reaction. This CI is assumed to decompose and form the initial RO_2 that initializes the autoxidation mechanism.
C107O2	Reactions with other RO_2 from the MCM chemistry. This leads to ring-opening of the formed cyclic alkoxy radicals (C10H15O2O and C107O, R1152-R1154 Supplementary Table 4) and formation of a new RO_2 in PRAM (C10H15O3O2 , R20 Supplementary Table 4).
C717O2	Assumed fragmentation product from the RO_2 + NO reactions
CH3COCH3	Assumed fragmentation product from the RO_2 + NO reactions

In addition to the species listed in Supplementary Table 3, all MCM RO_2 formed from oxidation of α-pinene, β-pinene and limonene with 10, 9 or 8 carbon atoms, in total 94 species, are involved in RO_2 + RO_2 reactions that lead to formation of closed shell dimers in PRAM (R85-R1118 and R1193-R1756)

Supplementary Table 4. List of all reactions and their reaction rates in PRAM[1]
If not others specified the unit of the reaction rates is cm^3 molecules^{-1} s^{-1}
PRAM applies the simple rate coefficients KDEC, KRO2NO and KRO2HO2 from MCMv3.3.1:

KDEC = 1E6 s^{-1}	*(Simplified rate coefficient for unimolecular reactions)*
*KRO2NO =2.7E-12*EXP(360/T) cm^3 molec^{-1} s^{-1}*	*(Used for the reactions between peroxy radicals and NO)*
*KRO2HO2 =2.91E-13*EXP(1300/T) cm^3 molec^{-1} s^{-1}*	*(Used for the reactions between peroxy radicals and HO_2)*

Reaction 1-6 and 11-1154 describes the autoxidation and formation of HOM after ozonolysis of α-pinene, limonene and carene.
Reaction 7-10 and 1155-1773 describes the autoxidation and formation of HOM after OH oxidation of α-pinene, β-pinene, limonene and carene. For the RO_2 + RO_2 reactions that lead to new closed shell HOM monomers and alkoxy radicals (R1119-R1151 and R1757-R1773 in Supplementary Table 4) we use the same simplified approach as in MCM, where each RO_2 is assumed to react with all other peroxy radicals from MCM (the so called RO_2 pool, denoted RO2*) at a single collective rate*[6].

**Reaction**	**Reaction rate**	**Comment / ref.**
APINENE + O3 → APINOOA	8.05E-16*EXP(-640/T)*0.6	MCMv3.3.1
APINENE + O3 → APINOOB	8.05E-16*EXP(-640/T)*0.4	MCMv3.3.1
LIMONENE + O3 → LIMOOA	2.80E-15*EXP(-770/T*0.730	MCMv3.3.1
LIMONENE + O3 → LIMOOB	2.80E-15*EXP(-770/T)*0.27	MCMv3.3.1
CARENE + O3 → APINOOA	3.70D-17*0.6	See motiv. above
CARENE + O3 → APINOOB	3.70D-17*0.4	See motiv. above
APINENE + OH → Products	1.2E-11*EXP(440/T)	MCMv3.3.1
BPINENE + OH → Products	2.38E-11*EXP(357/T)	MCMv3.3.1
LIMONENE + OH → Products	4.28E-11*EXP(401/T)	MCMv3.3.1
CARENE + OH → Products (same as α-pinene)	1.6E-11*EXP(500/T)	See motiv. above
**Initial Criegiee intermediate unimolecular reactions to peroxy radicals via the vinylhydroperoxide pathway[5] which continue to react according to the original MCMv3.3.1 mechanism (R1-R2, R4-R5) or initialize the peroxy radical autoxidation in PRAM (R3 and R6)**		
1. APINOOA → C107O2 + OH	KDEC*0.495	To MCM chem.
2. APINOOA → C109O2 + OH	KDEC*0.405	To MCM chem.
3. APINOOA → C10H15O2O2 + OH	KDEC*0.10	To PRAM
4. LIMOOA → LIMALAO2 + OH	KDEC*0.35	To MCM chem.
5. LIMOOA → LIMALBO2 + OH	KDEC*0.35	To MCM chem.
6. LIMOOA→ C10H15O2O2 + OH	KDEC*0.30	To PRAM
**Lumped reactions describing the initial reactions between a monoterpene (MT) and OH forming a RO_2 with 3 oxygen atoms ($C_{10}H_{17}OO_2$) that rapidly undergo a first intramolecular hydrogen shift and O_2 addition and form a new RO_2 with 5 oxygen atoms ($C_{10}H_{17}O_3O_2$).**		
**MT + OH → $C_{10}H_{17}OO_2$**		
**$C_{10}H_{17}OO_2$ + O_2 → $C_{10}H_{17}O_3O_2$ (Fast)**		
7. APINENE + OH → C10H17O3O2	0.025*1.2E-11*EXP(440/T)	To PRAM
8. BPINENE + OH → C10H17O3O2	0.01*2.38E-11*EXP(357/T)	To PRAM
9. LIMONENE + OH → C10H17O3O2	0.01*4.28E-11*EXP(401/T)	To PRAM
10. CARENE + OH → C10H17O3O2	0.025*1.6E-11*EXP(500/T)	To PRAM

Peroxy radical autoxidation and HOM formation after O_3 oxidation of monoterpenes (R11-R1154)

**Autoxidation reaction rates (H-shifts + O_2)**		
11. C10H15O2O2 → C10H15O4O2	1.2E17*EXP(-12077/T)	Unit s^{-1}
12. C10H15O3O2 → C10H15O5O2	1.2E18*EXP(-12077/T)	Unit s^{-1}
13. C10H15O4O2 → C10H15O6O2	1.2E18*EXP(-12077/T)	Unit s^{-1}
14. C10H15O5O2 → C10H15O7O2	6E17*EXP(-12077/T)	Unit s^{-1}
15. C10H15O6O2 → C10H15O8O2	2E17*EXP(-12077/T)	Unit s^{-1}
16. C10H15O7O2 → C10H15O9O2	1.2E17*EXP(-12077/T)	Unit s^{-1}
17. C10H15O8O2 → C10H15O10O2	1.2E17*EXP(-12077/T)	Unit s^{-1}
18. C10H15O9O2 → C10H15O11O2	1.2E16*EXP(-12077/T)	Unit s^{-1}
19. C10H15O10O2 → C10H15O12O2	0.0	Unit s^{-1}
**Isomerization of alkoxy radicals (RO) followed by reaction with O_2 to make new peroxy radicals:**		
**RO· → ·ROH**		
**·ROH + O_2 → RO_2·**		
**Net: RO· + O_2 → RO_2·**		
20. C10H15O2O → C10H15O3O2	KDEC	Unit s^{-1}
21. C10H15O3O → C10H15O4O2	KDEC	Unit s^{-1}
22. C10H15O4O → C10H15O5O2	KDEC	Unit s^{-1}
23. C10H15O5O → C10H15O6O2	KDEC	Unit s^{-1}
24. C10H15O6O → C10H15O7O2	KDEC	Unit s^{-1}
25. C10H15O7O → C10H15O8O2	KDEC	Unit s^{-1}
26. C10H15O8O → C10H15O9O2	KDEC	Unit s^{-1}
27. C10H15O9O → C10H15O10O2	KDEC	Unit s^{-1}
28. C10H15O10O → C10H15O11O2	KDEC	Unit s^{-1}
29. C10H15O11O → C10H15O12O2	KDEC	Unit s^{-1}
**Peroxy radicals reacting with NO and forming an alkoxy radical and NO_2 (RO_2 + NO → RO + NO_2)**		
*The rate for the reactions between autoxidation RO_2 and NO was set to the same values used in MCMv3.3.1 (*KRO2NO)		
30. C10H15O2O2 + NO → C10H15O2O + NO_2	KRO2NO*1.0	

31. C10H15O3O2 + NO → C10H15O3O + NO_2	KRO2NO*1.0
32. C10H15O4O2 + NO → C10H15O4O + NO_2	KRO2NO*1.0
33. C10H15O5O2 + NO → C10H15O5O + NO_2	KRO2NO*0.9
34. C10H15O6O2 + NO → C10H15O6O + NO_2	KRO2NO*0.85
35. C10H15O7O2 + NO → C10H15O7O + NO_2	KRO2NO*0.8
36. C10H15O8O2 + NO → C10H15O8O + NO_2	KRO2NO*0.6
37. C10H15O9O2 + NO → C10H15O9O + NO_2	KRO2NO*0.5
38. C10H15O10O2 + NO → C10H15O10O + NO_2	KRO2NO*0.3
39. C10H15O11O2 + NO → C10H15O11O + NO_2	0.0
40. C10H15O12O2 + NO → C10H15O12O + NO_2	0.0

Lumped reactions describing the fragmentation of autoxidation products when they react with NO (RO_2 + NO → RO + NO_2) followed by fast fragmentation of the formed alkoxy radicals).
The fragmentation products are assumed to be the MCM compounds C717O2 (a peroxy radical) and CH3COCH3 (acetone) irrespectively of which autoxidation peroxy radical that react with NO. This is a simplification that violates the mass balance for O atoms but not for the N and C atoms.

41. C10H15O2O2 + NO → C717O2 + CH3COCH3 + NO2	0.0
42. C10H15O3O2 + NO → C717O2 + CH3COCH3+NO2	0.0
43. C10H15O4O2 + NO → C717O2 + CH3COCH3 + NO2	0.0
44. C10H15O5O2 + NO → C717O2 + CH3COCH3+NO2	KRO2NO*0.04
45. C10H15O6O2 + NO → C717O2 + CH3COCH3 + NO2	KRO2NO*0.06
46. C10H15O7O2 + NO → C717O2 + CH3COCH3+NO2	KRO2NO*0.08
47. C10H15O8O2 + NO → C717O2 + CH3COCH3 + NO2	KRO2NO*0.16
48. C10H15O9O2 + NO → C717O2 + CH3COCH3+NO2	KRO2NO*0.20
49. C10H15O10O2 + NO → C717O2 + CH3COCH3 + NO2	KRO2NO*0.28
50. C10H15O11O2 + NO → C717O2 + CH3COCH3+NO2	KRO2NO*0.40
51. C10H15O12O2 + NO → C717O2 + CH3COCH3+NO2	KRO2NO*0.40

Peroxy radicals reacting with NO forming monomers with an additional carbonyl group:
RO_2 + NO → RO + NO_2
RO + O_2 → RCHO + HO_2 (Fast)
Net: RO_2 + NO + O_2→ RCHO + NO_2 + HO_2

52. C10H15O2O2 + NO → C10H14O3 + NO_2 + HO_2	0.0
53. C10H15O3O2 + NO → C10H14O4 + NO_2 + HO_2	0.0
54. C10H15O4O2 + NO → C10H14O5 + NO_2 + HO_2	0.0
55. C10H15O5O2 + NO → C10H14O6 + NO_2 + HO_2	KRO2NO*0.042
56. C10H15O6O2 + NO → C10H14O7 + NO_2 + HO_2	KRO2NO*0.063
57. C10H15O7O2 + NO → C10H14O8 + NO_2 + HO_2	KRO2NO*0.084
58. C10H15O8O2 + NO → C10H14O9 + NO_2 + HO_2	KRO2NO*0.168
59. C10H15O9O2 + NO → C10H14O10 + NO_2 + HO_2	KRO2NO*0.210
60. C10H15O10O2 + NO → C10H14O11 + NO_2 + HO_2	KRO2NO*0.294
61. C10H15O11O2 + NO → C10H14O12 + NO_2 + HO_2	KRO2NO*0.420
62. C10H15O12O2 + NO → C10H14O13 + NO_2 + HO_2	KRO2NO*0.420

Peroxy radicals reacting with NO forming compounds with a nitrate functional group (RO_2 + NO → RNO_3)

63. C10H15O2O2 + NO → C10H15O2NO3	0.0
64. C10H15O3O2 + NO → C10H15O3NO3	0.0
65. C10H15O4O2 + NO → C10H15O4NO3	0.0
66. C10H15O5O2 + NO → C10H15O5NO3	KRO2NO*0.018
67. C10H15O6O2 + NO → C10H15O6NO3	KRO2NO*0.027
68. C10H15O7O2 + NO → C10H15O7NO3	KRO2NO*0.036
69. C10H15O8O2 + NO → C10H15O8NO3	KRO2NO*0.072
70. C10H15O9O2 + NO → C10H15O9NO3	KRO2NO*0.090
71. C10H15O10O2 + NO → C10H15O10NO3	KRO2NO*0.126
72. C10H15O11O2 + NO → C10H15O11NO3	KRO2NO*0.180
73. C10H15O12O2 + NO → C10H15O12NO3	KRO2NO*0.180

Peroxy radicals reacting with HO_2 forming compounds with an hydrogen peroxide functional group (RO_2 + HO_2 → ROOH + O_2)

74. C10H15O2O2 + HO2 → C10H16O4iso1	KRO2HO2
75. C10H15O3O2 + HO2 → C10H16O5iso1	KRO2HO2
76. C10H15O4O2 + HO2 → C10H16O6iso1	KRO2HO2
77. C10H15O5O2 + HO2 → C10H16O7iso1	KRO2HO2
78. C10H15O6O2 + HO2 → C10H16O8iso1	KRO2HO2
79. C10H15O7O2 + HO2 → C10H16O9iso1	KRO2HO2
80. C10H15O8O2 + HO2 → C10H16O10	KRO2HO2
81. C10H15O9O2 + HO2 → C10H16O11	KRO2HO2
82. C10H15O10O2 + HO2 → C10H16O12	KRO2HO2
83. C10H15O11O2 + HO2 → C10H16O13	KRO2HO2
84. C10H15O12O2 + HO2 → C10H16O14	KRO2HO2

Peroxy radicals reacting with other peroxy radicals forming dimers (RO_2 + RO_2 → ROOR + O_2)

85. APINAO2 + C10H15O2O2 → C20H30O5	1E-13
86. APINBO2 + C10H15O2O2 → C20H30O5	1E-13
87. APINCO2 + C10H15O2O2 → C20H30O5	1E-13

88. C107O2 + C10H15O2O2 → C20H30O6	1E-13	
89. C109O2 + C10H15O2O2 → C20H30O6	1E-13	
90. C106O2 + C10H15O2O2 → C20H30O7	1E-13	
91. C920CO3 + C10H15O2O2 → C20H30O7	1E-13	
92. C108O2 + C10H15O2O2 → C20H30O7	1E-13	
93. PINALO2 + C10H15O2O2 → C20H30O6	1E-13	
94. C96CO3 + C10H15O2O2 → C20H30O6	1E-13	
95. C923CO3 + C10H15O2O2 → C20H30O6	1E-13	
96. LIMAO2 + C10H15O2O2 → C20H30O5	1E-13	
97. LIMALBO2 + C10H15O2O2 → C20H30O6	1E-13	
98. LIMCO2 + C10H15O2O2 → C20H30O5	1E-13	
99. LIMALO2 + C10H15O2O2 → C20H30O6	1E-13	
100. LIMBO2 + C10H15O2O2 → C20H30O5	1E-13	
101. LIMALAO2 + C10H15O2O2 → C20H30O6	1E-13	
102. BPINAO2 + C10H15O2O2 → C20H30O5	1E-13	
103. BPINBO2 + C10H15O2O2 → C20H30O5	1E-13	
104. BPINCO2 + C10H15O2O2 → C20H30O5	1E-13	
105. C918CO3 + C10H15O2O2 → C20H30O6	1E-13	
106. NAPINAO2 + C10H15O2O2 → C20H31O4NO3	1E-13	HOM dimers containing NO_3
107. NAPINBO2 + C10H15O2O2 → C20H31O4NO3	1E-13	HOM dimers containing NO_3
108. NC102O2 + C10H15O2O2 → C20H31O5NO3	1E-13	HOM dimers containing NO_3
109. NC101O2 + C10H15O2O2 → C20H31O5NO3	1E-13	HOM dimers containing NO_3
110. NLIMO2 + C10H15O2O2 → C20H31O4NO3	1E-13	HOM dimers containing NO_3
111. NLIMALO2 + C10H15O2O2 → C20H31O5NO3	1E-13	HOM dimers containing NO_3
112. NC91CO3 + C10H15O2O2 → C20H31O5NO3	1E-13	HOM dimers containing NO_3
113. NBPINAO2 + C10H15O2O2 → C20H31O4NO3	1E-13	HOM dimers containing NO_3
114. NBPINBO2 + C10H15O2O2 → C20H31O4NO3	1E-13	HOM dimers containing NO_3
115. C96O2 + C10H15O2O2 → C19H28O5	1E-13	
116. C89CO3 + C10H15O2O2 → C19H28O6	1E-13	
117. C920O2 + C10H15O2O2 → C19H28O6	1E-13	
118. C97O2 + C10H15O2O2 → C19H28O6	1E-13	
119. C85CO3 + C10H15O2O2 → C19H28O6	1E-13	
120. C811CO3 + C10H15O2O2 → C19H28O7	1E-13	
121. C921O2 + C10H15O2O2 → C19H28O7	1E-13	
122. C98O2 + C10H15O2O2 → C19H28O7	1E-13	
123. C922O2+ C10H15O2O2 → C19H28O8	1E-13	
124. C923O2 + C10H15O2O2 → C19H28O5	1E-13	
125. C924O2 + C10H15O2O2 → C19H28O6	1E-13	
126. C816CO3 + C10H15O2O2 → C19H28O6	1E-13	
127. NORLIMO2 + C10H15O2O2 → C19H28O7	1E-13	
128. LMKAO2 + C10H15O2O2 → C19H28O6	1E-13	
129. LMKBO2 + C10H15O2O2 → C19H28O6	1E-13	
130. C926O2 + C10H15O2O2 → C19H28O7	1E-13	
131. C817CO3 + C10H15O2O2 → C19H28O7	1E-13	
132. LMLKAO2 + C10H15O2O2 → C19H28O7	1E-13	
133. LMLKBO2 + C10H15O2O2 → C19H28O7	1E-13	
134. C823CO3 + C10H15O2O2 → C19H28O7	1E-13	
135. C925O2 + C10H15O2O2 → C19H28O8	1E-13	
136. NOPINAO2+ C10H15O2O2 → C19H28O5	1E-13	
137. NOPINBO2+ C10H15O2O2 → C19H28O5	1E-13	
138. NOPINCO2+ C10H15O2O2 → C19H28O5	1E-13	
139. NOPINDO2+ C10H15O2O2 → C19H28O5	1E-13	
140. C918O2+ C10H15O2O2 → C19H28O6	1E-13	
141. C9DCO2+ C10H15O2O2 → C19H28O6	1E-13	
142. C915O2+ C10H15O2O2 → C19H28O6	1E-13	
143. C917O2+ C10H15O2O2 → C19H28O6	1E-13	
144. C919O2+ C10H15O2O2 → C19H28O7	1E-13	
145. C914O2+ C10H15O2O2 → C19H28O7	1E-13	
146. C916O2+ C10H15O2O2 → C19H28O7	1E-13	
147. C88CO3+ C10H15O2O2 → C19H28O7	1E-13	
148. C87CO3+ C10H15O2O2 → C19H28O8	1E-13	
149. C822CO3+ C10H15O2O2 → C19H28O6	1E-13	
150. NLMKAO2+ C10H15O2O2 → C19H29O5NO3	1E-13	
151. C85O2 + C10H15O2O2 → C18H26O5	1E-13	
152. C89O2 + C10H15O2O2 → C18H26O5	1E-13	
153. C86O2 + C10H15O2O2 → C18H26O6	1E-13	
154. C811O2 + C10H15O2O2 → C18H26O6	1E-13	
155. C810O2 + C10H15O2O2 → C18H26O6	1E-13	
156. C812O2 + C10H15O2O2 → C18H26O7	1E-13	
157. C813O2 + C10H15O2O2 → C18H26O5	1E-13	
158. C729CO3 + C10H15O2O2 → C18H26O6	1E-13	
159. C816O2 + C10H15O2O2 → C18H26O5	1E-13	

160. C817O2 + C10H15O2O2 → C18H26O6	1E-13	
161. C826O2 + C10H15O2O2 → C18H26O7	1E-13	
162. C822O2 + C10H15O2O2 → C18H26O5	1E-13	
163. C818O2 + C10H15O2O2 → C18H26O7	1E-13	
164. C823O2 + C10H15O2O2 → C18H26O6	1E-13	
165. C819O2 + C10H15O2O2 → C18H26O7	1E-13	
166. C727CO3 + C10H15O2O2 → C18H26O7	1E-13	
167. C731CO3 + C10H15O2O2 → C18H26O7	1E-13	
168. C824O2 + C10H15O2O2 → C18H26O6	1E-13	
169. C820O2 + C10H15O2O2 → C18H26O8	1E-13	
170. C825O2 + C10H15O2O2 → C18H26O7	1E-13	
171. C821O2 + C10H15O2O2 → C18H26O7	1E-13	
172. C732CO3 + C10H15O2O2 → C18H26O8	1E-13	
173. C8BCO2 + C10H15O2O2 → C18H26O4	1E-13	
174. C88O2 + C10H15O2O2 → C18H26O6	1E-13	
175. C718CO3 + C10H15O2O2 → C18H26O7	1E-13	
176. C87O2 + C10H15O2O2 → C18H26O7	1E-13	
177. C721CO3 + C10H15O2O2 → C18H26O7	1E-13	
178. NC826O2 + C10H15O2O2 → C18H27O6NO3	1E-13	
179. APINAO2 + C10H15O3O2 → C20H30O6	1E-13	
180. APINBO2 + C10H15O3O2 → C20H30O6	1E-13	
181. APINCO2 + C10H15O3O2 → C20H30O6	1E-13	
182. C107O2 + C10H15O3O2 → C20H30O7	1E-13	
183. C109O2 + C10H15O3O2 → C20H30O7	1E-13	
184. C106O2 + C10H15O3O2 → C20H30O8	1E-13	
185. C920CO3 + C10H15O3O2 → C20H30O8	1E-13	
186. C108O2 + C10H15O3O2 → C20H30O8	1E-13	
187. PINALO2 + C10H15O3O2 → C20H30O7	1E-13	
188. C96CO3 + C10H15O3O2 → C20H30O7	1E-13	
189. C923CO3 + C10H15O3O2 → C20H30O7	1E-13	
190. LIMAO2 + C10H15O3O2 → C20H30O6	1E-13	
191. LIMALBO2 + C10H15O3O2 → C20H30O7	1E-13	
192. LIMCO2 + C10H15O3O2 → C20H30O6	1E-13	
193. LIMALO2 + C10H15O3O2 → C20H30O7	1E-13	
194. LIMBO2 + C10H15O3O2 → C20H30O6	1E-13	
195. LIMALAO2 + C10H15O3O2 → C20H30O7	1E-13	
196. BPINAO2 + C10H15O3O2 → C20H30O6	1E-13	
197. BPINBO2 + C10H15O3O2 → C20H30O6	1E-13	
198. BPINCO2 + C10H15O3O2 → C20H30O6	1E-13	
199. C918CO3 + C10H15O3O2 → C20H30O7	1E-13	
200. NAPINAO2 + C10H15O3O2 → C20H31O5NO3	1E-13	HOM dimers containing NO_3
201. NAPINBO2 + C10H15O3O2 → C20H31O5NO3	1E-13	HOM dimers containing NO_3
202. NC102O2 + C10H15O3O2 → C20H31O6NO3	1E-13	HOM dimers containing NO_3
203. NC101O2 + C10H15O3O2 → C20H31O6NO3	1E-13	HOM dimers containing NO_3
204. NLIMO2 + C10H15O3O2 → C20H31O5NO3	1E-13	HOM dimers containing NO_3
205. NLIMALO2 + C10H15O3O2 → C20H31O6NO3	1E-13	HOM dimers containing NO_3
206. NC91CO3 + C10H15O3O2 → C20H31O6NO3	1E-13	HOM dimers containing NO_3
207. NBPINAO2 + C10H15O3O2 → C20H31O5NO3	1E-13	HOM dimers containing NO_3
208. NBPINBO2 + C10H15O3O2 → C20H31O5NO3	1E-13	HOM dimers containing NO_3
209. C96O2 + C10H15O3O2 → C19H28O6	1E-13	
210. C89CO3 + C10H15O3O2 → C19H28O7	1E-13	
211. C920O2 + C10H15O3O2 → C19H28O7	1E-13	
212. C97O2 + C10H15O3O2 → C19H28O7	1E-13	
213. C85CO3 + C10H15O3O2 → C19H28O7	1E-13	
214. C811CO3 + C10H15O3O2 → C19H28O8	1E-13	
215. C921O2 + C10H15O3O2 → C19H28O8	1E-13	
216. C98O2 + C10H15O3O2 → C19H28O8	1E-13	
217. C922O2+ C10H15O3O2 → C19H28O9	1E-13	
218. C923O2 + C10H15O3O2 → C19H28O6	1E-13	
219. C924O2 + C10H15O3O2 → C19H28O7	1E-13	
220. C816CO3 + C10H15O3O2 → C19H28O7	1E-13	
221. NORLIMO2 + C10H15O3O2 → C19H28O8	1E-13	
222. LMKAO2 + C10H15O3O2 → C19H28O7	1E-13	
223. LMKBO2 + C10H15O3O2 → C19H28O7	1E-13	
224. C926O2 + C10H15O3O2 → C19H28O8	1E-13	
225. C817CO3 + C10H15O3O2 → C19H28O8	1E-13	
226. LMLKAO2 + C10H15O3O2 → C19H28O8	1E-13	
227. LMLKBO2 + C10H15O3O2 → C19H28O8	1E-13	
228. C823CO3 + C10H15O3O2 → C19H28O8	1E-13	
229. C925O2 + C10H15O3O2 → C19H28O9	1E-13	
230. NOPINAO2+ C10H15O3O2 → C19H28O6	1E-13	
231. NOPINBO2+ C10H15O3O2 → C19H28O6	1E-13	

232. NOPINCO2+ C10H15O3O2 → C19H28O6	1E-13	
233. NOPINDO2+ C10H15O3O2 → C19H28O6	1E-13	
234. C918O2+ C10H15O3O2 → C19H28O7	1E-13	
235. C9DCO2+ C10H15O3O2 → C19H28O7	1E-13	
236. C915O2+ C10H15O3O2 → C19H28O7	1E-13	
237. C917O2+ C10H15O3O2 → C19H28O7	1E-13	
238. C919O2+ C10H15O3O2 → C19H28O8	1E-13	
239. C914O2+ C10H15O3O2 → C19H28O8	1E-13	
240. C916O2+ C10H15O3O2 → C19H28O8	1E-13	
241. C88CO3+ C10H15O3O2 → C19H28O8	1E-13	
242. C87CO3+ C10H15O3O2 → C19H28O9	1E-13	
243. C822CO3+ C10H15O3O2 → C19H28O7	1E-13	
244. NLMKAO2+ C10H15O3O2 → C19H29O6NO3	1E-13	

245. C85O2 + C10H15O3O2 → C18H26O6	1E-13	
246. C89O2 + C10H15O3O2 → C18H26O6	1E-13	
247. C86O2 + C10H15O3O2 → C18H26O7	1E-13	
248. C811O2 + C10H15O3O2 → C18H26O7	1E-13	
249. C810O2 + C10H15O3O2 → C18H26O7	1E-13	
250. C812O2 + C10H15O3O2 → C18H26O8	1E-13	
251. C813O2 + C10H15O3O2 → C18H26O6	1E-13	
252. C729CO3 + C10H15O3O2 → C18H26O7	1E-13	
253. C816O2 + C10H15O3O2 → C18H26O6	1E-13	
254. C817O2 + C10H15O3O2 → C18H26O7	1E-13	
255. C826O2 + C10H15O3O2 → C18H26O8	1E-13	
256. C822O2 + C10H15O3O2 → C18H26O6	1E-13	
257. C818O2 + C10H15O3O2 → C18H26O8	1E-13	
258. C823O2 + C10H15O3O2 → C18H26O7	1E-13	
259. C819O2 + C10H15O3O2 → C18H26O8	1E-13	
260. C727CO3 + C10H15O3O2 → C18H26O8	1E-13	
261. C731CO3 + C10H15O3O2 → C18H26O8	1E-13	
262. C824O2 + C10H15O3O2 → C18H26O7	1E-13	
263. C820O2 + C10H15O3O2 → C18H26O9	1E-13	
264. C825O2 + C10H15O3O2 → C18H26O8	1E-13	
265. C821O2 + C10H15O3O2 → C18H26O8	1E-13	
266. C732CO3 + C10H15O3O2 → C18H26O9	1E-13	
267. C8BCO2 + C10H15O3O2 → C18H26O5	1E-13	
268. C88O2 + C10H15O3O2 → C18H26O7	1E-13	
269. C718CO3 + C10H15O3O2 → C18H26O8	1E-13	
270. C87O2 + C10H15O3O2 → C18H26O8	1E-13	
271. C721CO3 + C10H15O3O2 → C18H26O8	1E-13	
272. NC826O2 + C10H15O3O2 → C18H27O7NO3	1E-13	

273. APINAO2 + C10H15O4O2 → C20H30O7	1E-13	
274. APINBO2 + C10H15O4O2 → C20H30O7	1E-13	
275. APINCO2 + C10H15O4O2 → C20H30O7	1E-13	
276. C107O2 + C10H15O4O2 → C20H30O8	1E-13	
277. C109O2 + C10H15O4O2 → C20H30O8	1E-13	
278. C106O2 + C10H15O4O2 → C20H30O9	1E-13	
279. C920CO3 + C10H15O4O2 → C20H30O9	1E-13	
280. C108O2 + C10H15O4O2 → C20H30O9	1E-13	
281. PINALO2 + C10H15O4O2 → C20H30O8	1E-13	
282. C96CO3 + C10H15O4O2 → C20H30O8	1E-13	
283. C923CO3 + C10H15O4O2 → C20H30O8	1E-13	
284. LIMAO2 + C10H15O4O2 → C20H30O7	1E-13	
285. LIMALBO2 + C10H15O4O2 → C20H30O8	1E-13	
286. LIMCO2 + C10H15O4O2 → C20H30O7	1E-13	
287. LIMALO2 + C10H15O4O2 → C20H30O8	1E-13	
288. LIMBO2 + C10H15O4O2 → C20H30O7	1E-13	
289. LIMALAO2 + C10H15O4O2 → C20H30O8	1E-13	
290. BPINAO2 + C10H15O4O2 → C20H30O7	1E-13	
291. BPINBO2 + C10H15O4O2 → C20H30O7	1E-13	
292. BPINCO2 + C10H15O4O2 → C20H30O7	1E-13	
293. C918CO3 + C10H15O4O2 → C20H30O8	1E-13	
294. NAPINAO2 + C10H15O4O2 → C20H31O6NO3	1E-13	HOM dimers containing NO_3
295. NAPINBO2 + C10H15O4O2 → C20H31O6NO3	1E-13	HOM dimers containing NO_3
296. NC102O2 + C10H15O4O2 → C20H31O7NO3	1E-13	HOM dimers containing NO_3
297. NC101O2 + C10H15O4O2 → C20H31O7NO3	1E-13	HOM dimers containing NO_3
298. NLIMO2 + C10H15O4O2 → C20H31O6NO3	1E-13	HOM dimers containing NO_3
299. NLIMALO2 + C10H15O4O2 → C20H31O7NO3	1E-13	HOM dimers containing NO_3
300. NC91CO3 + C10H15O4O2 → C20H31O7NO3	1E-13	HOM dimers containing NO_3
301. NBPINAO2 + C10H15O4O2 → C20H31O6NO3	1E-13	HOM dimers containing NO_3
302. NBPINBO2 + C10H15O4O2 → C20H31O6NO3	1E-13	HOM dimers containing NO_3

303. C96O2 + C10H15O4O2 → C19H28O7	1E-13
304. C89CO3 + C10H15O4O2 → C19H28O8	1E-13
305. C920O2 + C10H15O4O2 → C19H28O8	1E-13
306. C97O2 + C10H15O4O2 → C19H28O8	1E-13
307. C85CO3 + C10H15O4O2 → C19H28O8	1E-13
308. C811CO3 + C10H15O4O2 → C19H28O9	1E-13
309. C921O2 + C10H15O4O2 → C19H28O9	1E-13
310. C98O2 + C10H15O4O2 → C19H28O9	1E-13
311. C922O2+ C10H15O4O2 → C19H28O10	1E-13
312. C923O2 + C10H15O4O2 → C19H28O7	1E-13
313. C924O2 + C10H15O4O2 → C19H28O8	1E-13
314. C816CO3 + C10H15O4O2 → C19H28O8	1E-13
315. NORLIMO2 + C10H15O4O2 → C19H28O9	1E-13
316. LMKAO2 + C10H15O4O2 → C19H28O8	1E-13
317. LMKBO2 + C10H15O4O2 → C19H28O8	1E-13
318. C926O2 + C10H15O4O2 → C19H28O9	1E-13
319. C817CO3 + C10H15O4O2 → C19H28O9	1E-13
320. LMLKAO2 + C10H15O4O2 → C19H28O9	1E-13
321. LMLKBO2 + C10H15O4O2 → C19H28O9	1E-13
322. C823CO3 + C10H15O4O2 → C19H28O9	1E-13
323. C925O2 + C10H15O4O2 → C19H28O10	1E-13
324. NOPINAO2+ C10H15O4O2 → C19H28O7	1E-13
325. NOPINBO2+ C10H15O4O2 → C19H28O7	1E-13
326. NOPINCO2+ C10H15O4O2 → C19H28O7	1E-13
327. NOPINDO2+ C10H15O4O2 → C19H28O7	1E-13
328. C918O2+ C10H15O4O2 → C19H28O8	1E-13
329. C9DCO2+ C10H15O4O2 → C19H28O8	1E-13
330. C915O2+ C10H15O4O2 → C19H28O8	1E-13
331. C917O2+ C10H15O4O2 → C19H28O8	1E-13
332. C919O2+ C10H15O4O2 → C19H28O9	1E-13
333. C914O2+ C10H15O4O2 → C19H28O9	1E-13
334. C916O2+ C10H15O4O2 → C19H28O9	1E-13
335. C88CO3+ C10H15O4O2 → C19H28O9	1E-13
336. C87CO3+ C10H15O4O2 → C19H28O10	1E-13
337. C822CO3+ C10H15O4O2 → C19H28O8	1E-13
338. NLMKAO2+ C10H15O4O2 → C19H29O7NO3	1E-13

339. C85O2 + C10H15O4O2 → C18H26O7	1E-13
340. C89O2 + C10H15O4O2 → C18H26O7	1E-13
341. C86O2 + C10H15O4O2 → C18H26O8	1E-13
342. C811O2 + C10H15O4O2 → C18H26O8	1E-13
343. C810O2 + C10H15O4O2 → C18H26O8	1E-13
344. C812O2 + C10H15O4O2 → C18H26O9	1E-13
345. C813O2 + C10H15O4O2 → C18H26O7	1E-13
346. C729CO3 + C10H15O4O2 → C18H26O8	1E-13
347. C816O2 + C10H15O4O2 → C18H26O7	1E-13
348. C817O2 + C10H15O4O2 → C18H26O8	1E-13
349. C826O2 + C10H15O4O2 → C18H26O9	1E-13
350. C822O2 + C10H15O4O2 → C18H26O7	1E-13
351. C818O2 + C10H15O4O2 → C18H26O9	1E-13
352. C823O2 + C10H15O4O2 → C18H26O8	1E-13
353. C819O2 + C10H15O4O2 → C18H26O9	1E-13
354. C727CO3 + C10H15O4O2 → C18H26O9	1E-13
355. C731CO3 + C10H15O4O2 → C18H26O9	1E-13
356. C824O2 + C10H15O4O2 → C18H26O8	1E-13
357. C820O2 + C10H15O4O2 → C18H26O10	1E-13
358. C825O2 + C10H15O4O2 → C18H26O9	1E-13
359. C821O2 + C10H15O4O2 → C18H26O9	1E-13
360. C732CO3 + C10H15O4O2 → C18H26O10	1E-13
361. C8BCO2 + C10H15O4O2 → C18H26O6	1E-13
362. C88O2 + C10H15O4O2 → C18H26O8	1E-13
363. C718CO3 + C10H15O4O2 → C18H26O9	1E-13
364. C87O2 + C10H15O4O2 → C18H26O9	1E-13
365. C721CO3 + C10H15O4O2 → C18H26O9	1E-13
366. NC826O2 + C10H15O4O2 → C18H27O8NO3	1E-13

367. APINAO2 + C10H15O5O2 → C20H30O8	5E-13
368. APINBO2 + C10H15O5O2 → C20H30O8	5E-13
369. APINCO2 + C10H15O5O2 → C20H30O8	5E-13
370. C107O2 + C10H15O5O2 → C20H30O9	5E-13
371. C109O2 + C10H15O5O2 → C20H30O9	5E-13
372. C106O2 + C10H15O5O2 → C20H30O10	5E-13
373. C920CO3 + C10H15O5O2 → C20H30O10	5E-13
374. C108O2 + C10H15O5O2 → C20H30O10	5E-13

375. PINALO2 + C10H15O5O2 → C20H30O9	5E-13	
376. C96CO3 + C10H15O5O2 → C20H30O9	5E-13	
377. C923CO3 + C10H15O5O2 → C20H30O9	5E-13	
378. LIMAO2 + C10H15O5O2 → C20H30O8	5E-13	
379. LIMALBO2 + C10H15O5O2 → C20H30O9	5E-13	
380. LIMCO2 + C10H15O5O2 → C20H30O8	5E-13	
381. LIMALO2 + C10H15O5O2 → C20H30O9	5E-13	
382. LIMBO2 + C10H15O5O2 → C20H30O8	5E-13	
383. LIMALAO2 + C10H15O5O2 → C20H30O9	5E-13	
384. BPINAO2 + C10H15O5O2 → C20H30O8	5E-13	
385. BPINBO2 + C10H15O5O2 → C20H30O8	5E-13	
386. BPINCO2 + C10H15O5O2 → C20H30O8	5E-13	
387. C918CO3 + C10H15O5O2 → C20H30O9	5E-13	
388. NAPINAO2 + C10H15O5O2 → C20H31O7NO3	5E-13	HOM dimers containing NO_3
389. NAPINBO2 + C10H15O5O2 → C20H31O7NO3	5E-13	HOM dimers containing NO_3
390. NC102O2 + C10H15O5O2 → C20H31O8NO3	5E-13	HOM dimers containing NO_3
391. NC101O2 + C10H15O5O2 → C20H31O8NO3	5E-13	HOM dimers containing NO_3
392. NLIMO2 + C10H15O5O2 → C20H31O7NO3	5E-13	HOM dimers containing NO_3
393. NLIMALO2 + C10H15O5O2 → C20H31O8NO3	5E-13	HOM dimers containing NO_3
394. NC91CO3 + C10H15O5O2 → C20H31O8NO3	5E-13	HOM dimers containing NO_3
395. NBPINAO2 + C10H15O5O2 → C20H31O7NO3	5E-13	HOM dimers containing NO_3
396. NBPINBO2 + C10H15O5O2 → C20H31O7NO3	5E-13	HOM dimers containing NO_3
397. C96O2 + C10H15O5O2 → C19H28O8	5E-13	
398. C89CO3 + C10H15O5O2 → C19H28O9	5E-13	
399. C920O2 + C10H15O5O2 → C19H28O9	5E-13	
400. C97O2 + C10H15O5O2 → C19H28O9	5E-13	
401. C85CO3 + C10H15O5O2 → C19H28O9	5E-13	
402. C811CO3 + C10H15O5O2 → C19H28O10	5E-13	
403. C921O2 + C10H15O5O2 → C19H28O10	5E-13	
404. C98O2 + C10H15O5O2 → C19H28O10	5E-13	
405. C922O2+ C10H15O5O2 → C19H28O11	5E-13	
406. C923O2 + C10H15O5O2 → C19H28O8	5E-13	
407. C924O2 + C10H15O5O2 → C19H28O9	5E-13	
408. C816CO3 + C10H15O5O2 → C19H28O9	5E-13	
409. NORLIMO2 + C10H15O5O2 → C19H28O10	5E-13	
410. LMKAO2 + C10H15O5O2 → C19H28O9	5E-13	
411. LMKBO2 + C10H15O5O2 → C19H28O9	5E-13	
412. C926O2 + C10H15O5O2 → C19H28O10	5E-13	
413. C817CO3 + C10H15O5O2 → C19H28O10	5E-13	
414. LMLKAO2 + C10H15O5O2 → C19H28O10	5E-13	
415. LMLKBO2 + C10H15O5O2 → C19H28O10	5E-13	
416. C823CO3 + C10H15O5O2 → C19H28O10	5E-13	
417. C925O2 + C10H15O5O2 → C19H28O11	5E-13	
418. NOPINAO2+ C10H15O5O2 → C19H28O8	5E-13	
419. NOPINBO2+ C10H15O5O2 → C19H28O8	5E-13	
420. NOPINCO2+ C10H15O5O2 → C19H28O8	5E-13	
421. NOPINDO2+ C10H15O5O2 → C19H28O8	5E-13	
422. C918O2+ C10H15O5O2 → C19H28O9	5E-13	
423. C9DCO2+ C10H15O5O2 → C19H28O9	5E-13	
424. C915O2+ C10H15O5O2 → C19H28O9	5E-13	
425. C917O2+ C10H15O5O2 → C19H28O9	5E-13	
426. C919O2+ C10H15O5O2 → C19H28O10	5E-13	
427. C914O2+ C10H15O5O2 → C19H28O10	5E-13	
428. C916O2+ C10H15O5O2 → C19H28O10	5E-13	
429. C88CO3+ C10H15O5O2 → C19H28O10	5E-13	
430. C87CO3+ C10H15O5O2 → C19H28O11	5E-13	
431. C822CO3+ C10H15O5O2 → C19H28O9	5E-13	
432. NLMKAO2+ C10H15O5O2 → C19H29O8NO3	5E-13	
433. C85O2 + C10H15O5O2 → C18H26O8	5E-13	
434. C89O2 + C10H15O5O2 → C18H26O8	5E-13	
435. C86O2 + C10H15O5O2 → C18H26O9	5E-13	
436. C811O2 + C10H15O5O2 → C18H26O9	5E-13	
437. C810O2 + C10H15O5O2 → C18H26O9	5E-13	
438. C812O2 + C10H15O5O2 → C18H26O10	5E-13	
439. C813O2 + C10H15O5O2 → C18H26O8	5E-13	
440. C729CO3 + C10H15O5O2 → C18H26O9	5E-13	
441. C816O2 + C10H15O5O2 → C18H26O8	5E-13	
442. C817O2 + C10H15O5O2 → C18H26O9	5E-13	
443. C826O2 + C10H15O5O2 → C18H26O10	5E-13	
444. C822O2 + C10H15O5O2 → C18H26O8	5E-13	
445. C818O2 + C10H15O5O2 → C18H26O10	5E-13	
446. C823O2 + C10H15O5O2 → C18H26O9	5E-13	

447. C819O2 + C10H15O5O2 → C18H26O10	5E-13	
448. C727CO3 + C10H15O5O2 → C18H26O10	5E-13	
449. C731CO3 + C10H15O5O2 → C18H26O10	5E-13	
450. C824O2 + C10H15O5O2 → C18H26O9	5E-13	
451. C820O2 + C10H15O5O2 → C18H26O11	5E-13	
452. C825O2 + C10H15O5O2 → C18H26O10	5E-13	
453. C821O2 + C10H15O5O2 → C18H26O10	5E-13	
454. C732CO3 + C10H15O5O2 → C18H26O11	5E-13	
455. C8BCO2 + C10H15O5O2 → C18H26O7	5E-13	
456. C88O2 + C10H15O5O2 → C18H26O9	5E-13	
457. C718CO3 + C10H15O5O2 → C18H26O10	5E-13	
458. C87O2 + C10H15O5O2 → C18H26O10	5E-13	
459. C721CO3 + C10H15O5O2 → C18H26O10	5E-13	
460. NC826O2 + C10H15O5O2 → C18H27O9NO3	5E-13	

461. APINAO2 + C10H15O6O2 → C20H30O9	2E-12	
462. APINBO2 + C10H15O6O2 → C20H30O9	2E-12	
463. APINCO2 + C10H15O6O2 → C20H30O9	2E-12	
464. C107O2 + C10H15O6O2 → C20H30O10	2E-12	
465. C109O2 + C10H15O6O2 → C20H30O10	2E-12	
466. C106O2 + C10H15O6O2 → C20H30O11	2E-12	
467. C920CO3 + C10H15O6O2 → C20H30O11	2E-12	
468. C108O2 + C10H15O6O2 → C20H30O11	2E-12	
469. PINALO2 + C10H15O6O2 → C20H30O10	2E-12	
470. C96CO3 + C10H15O6O2 → C20H30O10	2E-12	
471. C923CO3 + C10H15O6O2 → C20H30O10	2E-12	
472. LIMAO2 + C10H15O6O2 → C20H30O9	2E-12	
473. LIMALBO2 + C10H15O6O2 → C20H30O10	2E-12	
474. LIMCO2 + C10H15O6O2 → C20H30O9	2E-12	
475. LIMALO2 + C10H15O6O2 → C20H30O10	2E-12	
476. LIMBO2 + C10H15O6O2 → C20H30O9	2E-12	
477. LIMALAO2 + C10H15O6O2 → C20H30O10	2E-12	
478. BPINAO2 + C10H15O6O2 → C20H30O9	2E-12	
479. BPINBO2 + C10H15O6O2 → C20H30O9	2E-12	
480. BPINCO2 + C10H15O6O2 → C20H30O9	2E-12	
481. C918CO3 + C10H15O6O2 → C20H30O10	2E-12	
482. NAPINAO2 + C10H15O6O2 → C20H31O8NO3	2E-12	HOM dimers containing NO_3
483. NAPINBO2 + C10H15O6O2 → C20H31O8NO3	2E-12	HOM dimers containing NO_3
484. NC102O2 + C10H15O6O2 → C20H31O9NO3	2E-12	HOM dimers containing NO_3
485. NC101O2 + C10H15O6O2 → C20H31O9NO3	2E-12	HOM dimers containing NO_3
486. NLIMO2 + C10H15O6O2 → C20H31O8NO3	2E-12	HOM dimers containing NO_3
487. NLIMALO2 + C10H15O6O2 → C20H31O9NO3	2E-12	HOM dimers containing NO_3
488. NC91CO3 + C10H15O6O2 → C20H31O9NO3	2E-12	HOM dimers containing NO_3
489. NBPINAO2 + C10H15O6O2 → C20H31O8NO3	2E-12	HOM dimers containing NO_3
490. NBPINBO2 + C10H15O6O2 → C20H31O8NO3	2E-12	HOM dimers containing NO_3

491. C96O2 + C10H15O6O2 → C19H28O9	2E-12	
492. C89CO3 + C10H15O6O2 → C19H28O10	2E-12	
493. C920O2 + C10H15O6O2 → C19H28O10	2E-12	
494. C97O2 + C10H15O6O2 → C19H28O10	2E-12	
495. C85CO3 + C10H15O6O2 → C19H28O10	2E-12	
496. C811CO3 + C10H15O6O2 → C19H28O11	2E-12	
497. C921O2 + C10H15O6O2 → C19H28O11	2E-12	
498. C98O2 + C10H15O6O2 → C19H28O11	2E-12	
499. C922O2+ C10H15O6O2 → C19H28O12	2E-12	
500. C923O2 + C10H15O6O2 → C19H28O9	2E-12	
501. C924O2 + C10H15O6O2 → C19H28O10	2E-12	
502. C816CO3 + C10H15O6O2 → C19H28O10	2E-12	
503. NORLIMO2 + C10H15O6O2 → C19H28O11	2E-12	
504. LMKAO2 + C10H15O6O2 → C19H28O10	2E-12	
505. LMKBO2 + C10H15O6O2 → C19H28O10	2E-12	
506. C926O2 + C10H15O6O2 → C19H28O11	2E-12	
507. C817CO3 + C10H15O6O2 → C19H28O11	2E-12	
508. LMLKAO2 + C10H15O6O2 → C19H28O11	2E-12	
509. LMLKBO2 + C10H15O6O2 → C19H28O11	2E-12	
510. C823CO3 + C10H15O6O2 → C19H28O11	2E-12	
511. C925O2 + C10H15O6O2 → C19H28O12	2E-12	
512. NOPINAO2+ C10H15O6O2 → C19H28O9	2E-12	
513. NOPINBO2+ C10H15O6O2 → C19H28O9	2E-12	
514. NOPINCO2+ C10H15O6O2 → C19H28O9	2E-12	
515. NOPINDO2+ C10H15O6O2 → C19H28O9	2E-12	
516. C918O2+ C10H15O6O2 → C19H28O10	2E-12	
517. C9DCO2+ C10H15O6O2 → C19H28O10	2E-12	
518. C915O2+ C10H15O6O2 → C19H28O10	2E-12	

519. C917O2+ C10H15O6O2 → C19H28O10 2E-12
520. C919O2+ C10H15O6O2 → C19H28O11 2E-12
521. C914O2+ C10H15O6O2 → C19H28O11 2E-12
522. C916O2+ C10H15O6O2 → C19H28O11 2E-12
523. C88CO3+ C10H15O6O2 → C19H28O11 2E-12
524. C87CO3+ C10H15O6O2 → C19H28O12 2E-12
525. C822CO3+ C10H15O6O2 → C19H28O10 2E-12
526. NLMKAO2+ C10H15O6O2 → C19H29O9NO3 2E-12

527. C85O2 + C10H15O6O2 → C18H26O9 2E-12
528. C89O2 + C10H15O6O2 → C18H26O9 2E-12
529. C86O2 + C10H15O6O2 → C18H26O10 2E-12
530. C811O2 + C10H15O6O2 → C18H26O10 2E-12
531. C810O2 + C10H15O6O2 → C18H26O10 2E-12
532. C812O2 + C10H15O6O2 → C18H26O11 2E-12
533. C813O2 + C10H15O6O2 → C18H26O9 2E-12
534. C729CO3 + C10H15O6O2 → C18H26O10 2E-12
535. C816O2 + C10H15O6O2 → C18H26O9 2E-12
536. C817O2 + C10H15O6O2 → C18H26O10 2E-12
537. C826O2 + C10H15O6O2 → C18H26O11 2E-12
538. C822O2 + C10H15O6O2 → C18H26O9 2E-12
539. C818O2 + C10H15O6O2 → C18H26O11 2E-12
540. C823O2 + C10H15O6O2 → C18H26O10 2E-12
541. C819O2 + C10H15O6O2 → C18H26O11 2E-12
542. C727CO3 + C10H15O6O2 → C18H26O11 2E-12
543. C731CO3 + C10H15O6O2 → C18H26O11 2E-12
544. C824O2 + C10H15O6O2 → C18H26O10 2E-12
545. C820O2 + C10H15O6O2 → C18H26O12 2E-12
546. C825O2 + C10H15O6O2 → C18H26O11 2E-12
547. C821O2 + C10H15O6O2 → C18H26O11 2E-12
548. C732CO3 + C10H15O6O2 → C18H26O12 2E-12
549. C8BCO2 + C10H15O6O2 → C18H26O8 2E-12
550. C88O2 + C10H15O6O2 → C18H26O10 2E-12
551. C718CO3 + C10H15O6O2 → C18H26O11 2E-12
552. C87O2 + C10H15O6O2 → C18H26O11 2E-12
553. C721CO3 + C10H15O6O2 → C18H26O11 2E-12
554. NC826O2 + C10H15O6O2 → C18H27O10NO3 2E-12

555. APINAO2 + C10H15O7O2 → C20H30O10 3E-12
556. APINBO2 + C10H15O7O2 → C20H30O10 3E-12
557. APINCO2 + C10H15O7O2 → C20H30O10 3E-12
558. C107O2 + C10H15O7O2 → C20H30O11 3E-12
559. C109O2 + C10H15O7O2 → C20H30O11 3E-12
560. C106O2 + C10H15O7O2 → C20H30O12 3E-12
561. C920CO3 + C10H15O7O2 → C20H30O12 3E-12
562. C108O2 + C10H15O7O2 → C20H30O12 3E-12
563. PINALO2 + C10H15O7O2 → C20H30O11 3E-12
564. C96CO3 + C10H15O7O2 → C20H30O11 3E-12
565. C923CO3 + C10H15O7O2 → C20H30O11 3E-12
566. LIMAO2 + C10H15O7O2 → C20H30O10 3E-12
567. LIMALBO2 + C10H15O7O2 → C20H30O11 3E-12
568. LIMCO2 + C10H15O7O2 → C20H30O10 3E-12
569. LIMALO2 + C10H15O7O2 → C20H30O11 3E-12
570. LIMBO2 + C10H15O7O2 → C20H30O10 3E-12
571. LIMALAO2 + C10H15O7O2 → C20H30O11 3E-12
572. BPINAO2 + C10H15O7O2 → C20H30O10 3E-12
573. BPINBO2 + C10H15O7O2 → C20H30O10 3E-12
574. BPINCO2 + C10H15O7O2 → C20H30O10 3E-12
575. C918CO3 + C10H15O7O2 → C20H30O11 3E-12
576. NAPINAO2 + C10H15O7O2 → C20H31O9NO3 3E-12 HOM dimers containing NO_3
577. NAPINBO2 + C10H15O7O2 → C20H31O9NO3 3E-12 HOM dimers containing NO_3
578. NC102O2 + C10H15O7O2 → C20H31O10NO3 3E-12 HOM dimers containing NO_3
579. NC101O2 + C10H15O7O2 → C20H31O10NO3 3E-12 HOM dimers containing NO_3
580. NLIMO2 + C10H15O7O2 → C20H31O9NO3 3E-12 HOM dimers containing NO_3
581. NLIMALO2 + C10H15O7O2 → C20H31O10NO3 3E-12 HOM dimers containing NO_3
582. NC91CO3 + C10H15O7O2 → C20H31O10NO3 3E-12 HOM dimers containing NO_3
583. NBPINAO2 + C10H15O7O2 → C20H31O9NO3 3E-12 HOM dimers containing NO_3
584. NBPINBO2 + C10H15O7O2 → C20H31O9NO3 3E-12 HOM dimers containing NO_3

585. C96O2 + C10H15O7O2 → C19H28O10 3E-12
586. C89CO3 + C10H15O7O2 → C19H28O11 3E-12
587. C920O2 + C10H15O7O2 → C19H28O11 3E-12
588. C97O2 + C10H15O7O2 → C19H28O11 3E-12
589. C85CO3 + C10H15O7O2 → C19H28O11 3E-12

590. C811CO3 + C10H15O7O2 → C19H28O12	3E-12
591. C921O2 + C10H15O7O2 → C19H28O12	3E-12
592. C98O2 + C10H15O7O2 → C19H28O12	3E-12
593. C922O2+ C10H15O7O2 → C19H28O13	3E-12
594. C923O2 + C10H15O7O2 → C19H28O10	3E-12
595. C924O2 + C10H15O7O2 → C19H28O11	3E-12
596. C816CO3 + C10H15O7O2 → C19H28O11	3E-12
597. NORLIMO2 + C10H15O7O2 → C19H28O12	3E-12
598. LMKAO2 + C10H15O7O2 → C19H28O11	3E-12
599. LMKBO2 + C10H15O7O2 → C19H28O11	3E-12
600. C926O2 + C10H15O7O2 → C19H28O12	3E-12
601. C817CO3 + C10H15O7O2 → C19H28O12	3E-12
602. LMLKAO2 + C10H15O7O2 → C19H28O12	3E-12
603. LMLKBO2 + C10H15O7O2 → C19H28O12	3E-12
604. C823CO3 + C10H15O7O2 → C19H28O12	3E-12
605. C925O2 + C10H15O7O2 → C19H28O13	3E-12
606. NOPINAO2+ C10H15O7O2 → C19H28O10	3E-12
607. NOPINBO2+ C10H15O7O2 → C19H28O10	3E-12
608. NOPINCO2+ C10H15O7O2 → C19H28O10	3E-12
609. NOPINDO2+ C10H15O7O2 → C19H28O10	3E-12
610. C918O2+ C10H15O7O2 → C19H28O11	3E-12
611. C9DCO2+ C10H15O7O2 → C19H28O11	3E-12
612. C915O2+ C10H15O7O2 → C19H28O11	3E-12
613. C917O2+ C10H15O7O2 → C19H28O11	3E-12
614. C919O2+ C10H15O7O2 → C19H28O12	3E-12
615. C914O2+ C10H15O7O2 → C19H28O12	3E-12
616. C916O2+ C10H15O7O2 → C19H28O12	3E-12
617. C88CO3+ C10H15O7O2 → C19H28O12	3E-12
618. C87CO3+ C10H15O7O2 → C19H28O13	3E-12
619. C822CO3+ C10H15O7O2 → C19H28O11	3E-12
620. NLMKAO2+ C10H15O7O2 → C19H29O10NO3	3E-12
621. C85O2 + C10H15O7O2 → C18H26O10	3E-12
622. C89O2 + C10H15O7O2 → C18H26O10	3E-12
623. C86O2 + C10H15O7O2 → C18H26O11	3E-12
624. C811O2 + C10H15O7O2 → C18H26O11	3E-12
625. C810O2 + C10H15O7O2 → C18H26O11	3E-12
626. C812O2 + C10H15O7O2 → C18H26O12	3E-12
627. C813O2 + C10H15O7O2 → C18H26O10	3E-12
628. C729CO3 + C10H15O7O2 → C18H26O11	3E-12
629. C816O2 + C10H15O7O2 → C18H26O10	3E-12
630. C817O2 + C10H15O7O2 → C18H26O11	3E-12
631. C826O2 + C10H15O7O2 → C18H26O12	3E-12
632. C822O2 + C10H15O7O2 → C18H26O10	3E-12
633. C818O2 + C10H15O7O2 → C18H26O12	3E-12
634. C823O2 + C10H15O7O2 → C18H26O11	3E-12
635. C819O2 + C10H15O7O2 → C18H26O12	3E-12
636. C727CO3 + C10H15O7O2 → C18H26O12	3E-12
637. C731CO3 + C10H15O7O2 → C18H26O12	3E-12
638. C824O2 + C10H15O7O2 → C18H26O11	3E-12
639. C820O2 + C10H15O7O2 → C18H26O13	3E-12
640. C825O2 + C10H15O7O2 → C18H26O12	3E-12
641. C821O2 + C10H15O7O2 → C18H26O12	3E-12
642. C732CO3 + C10H15O7O2 → C18H26O13	3E-12
643. C8BCO2 + C10H15O7O2 → C18H26O9	3E-12
644. C88O2 + C10H15O7O2 → C18H26O11	3E-12
645. C718CO3 + C10H15O7O2 → C18H26O12	3E-12
646. C87O2 + C10H15O7O2 → C18H26O12	3E-12
647. C721CO3 + C10H15O7O2 → C18H26O12	3E-12
648. NC826O2 + C10H15O7O2 → C18H27O11NO3	3E-12
649. APINAO2 + C10H15O8O2 → C20H30O11	3E-12
650. APINBO2 + C10H15O8O2 → C20H30O11	3E-12
651. APINCO2 + C10H15O8O2 → C20H30O11	3E-12
652. C107O2 + C10H15O8O2 → C20H30O12	3E-12
653. C109O2 + C10H15O8O2 → C20H30O12	3E-12
654. C106O2 + C10H15O8O2 → C20H30O13	3E-12
655. C920CO3 + C10H15O8O2 → C20H30O13	3E-12
656. C108O2 + C10H15O8O2 → C20H30O13	3E-12
657. PINALO2 + C10H15O8O2 → C20H30O12	3E-12
658. C96CO3 + C10H15O8O2 → C20H30O12	3E-12
659. C923CO3 + C10H15O8O2 → C20H30O12	3E-12
660. LIMAO2 + C10H15O8O2 → C20H30O11	3E-12
661. LIMALBO2 + C10H15O8O2 → C20H30O12	3E-12

662. LIMCO2 + C10H15O8O2 → C20H30O11	3E-12	
663. LIMALO2 + C10H15O8O2 → C20H30O12	3E-12	
664. LIMBO2 + C10H15O8O2 → C20H30O11	3E-12	
665. LIMALAO2 + C10H15O8O2 → C20H30O12	3E-12	
666. BPINAO2 + C10H15O8O2 → C20H30O11	3E-12	
667. BPINBO2 + C10H15O8O2 → C20H30O11	3E-12	
668. BPINCO2 + C10H15O8O2 → C20H30O11	3E-12	
669. C918CO3 + C10H15O8O2 → C20H30O12	3E-12	
670. NAPINAO2 + C10H15O8O2 → C20H31O10NO3	3E-12	HOM dimers containing NO_3
671. NAPINBO2 + C10H15O8O2 → C20H31O10NO3	3E-12	HOM dimers containing NO_3
672. NC102O2 + C10H15O8O2 → C20H31O11NO3	3E-12	HOM dimers containing NO_3
673. NC101O2 + C10H15O8O2 → C20H31O11NO3	3E-12	HOM dimers containing NO_3
674. NLIMO2 + C10H15O8O2 → C20H31O10NO3	3E-12	HOM dimers containing NO_3
675. NLIMALO2 + C10H15O8O2 → C20H31O11NO3	3E-12	HOM dimers containing NO_3
676. NC91CO3 + C10H15O8O2 → C20H31O11NO3	3E-12	HOM dimers containing NO_3
677. NBPINAO2 + C10H15O8O2 → C20H31O10NO3	3E-12	HOM dimers containing NO_3
678. NBPINBO2 + C10H15O8O2 → C20H31O10NO3	3E-12	HOM dimers containing NO_3
679. C96O2 + C10H15O8O2 → C19H28O11	3E-12	
680. C89CO3 + C10H15O8O2 → C19H28O12	3E-12	
681. C920O2 + C10H15O8O2 → C19H28O12	3E-12	
682. C97O2 + C10H15O8O2 → C19H28O12	3E-12	
683. C85CO3 + C10H15O8O2 → C19H28O12	3E-12	
684. C811CO3 + C10H15O8O2 → C19H28O13	3E-12	
685. C921O2 + C10H15O8O2 → C19H28O13	3E-12	
686. C98O2 + C10H15O8O2 → C19H28O13	3E-12	
687. C922O2+ C10H15O8O2 → C19H28O14	3E-12	
688. C923O2 + C10H15O8O2 → C19H28O11	3E-12	
689. C924O2 + C10H15O8O2 → C19H28O12	3E-12	
690. C816CO3 + C10H15O8O2 → C19H28O12	3E-12	
691. NORLIMO2 + C10H15O8O2 → C19H28O13	3E-12	
692. LMKAO2 + C10H15O8O2 → C19H28O12	3E-12	
693. LMKBO2 + C10H15O8O2 → C19H28O12	3E-12	
694. C926O2 + C10H15O8O2 → C19H28O13	3E-12	
695. C817CO3 + C10H15O8O2 → C19H28O13	3E-12	
696. LMLKAO2 + C10H15O8O2 → C19H28O13	3E-12	
697. LMLKBO2 + C10H15O8O2 → C19H28O13	3E-12	
698. C823CO3 + C10H15O8O2 → C19H28O13	3E-12	
699. C925O2 + C10H15O8O2 → C19H28O14	3E-12	
700. NOPINAO2+ C10H15O8O2 → C19H28O11	3E-12	
701. NOPINBO2+ C10H15O8O2 → C19H28O11	3E-12	
702. NOPINCO2+ C10H15O8O2 → C19H28O11	3E-12	
703. NOPINDO2+ C10H15O8O2 → C19H28O11	3E-12	
704. C918O2+ C10H15O8O2 → C19H28O12	3E-12	
705. C9DCO2+ C10H15O8O2 → C19H28O12	3E-12	
706. C915O2+ C10H15O8O2 → C19H28O12	3E-12	
707. C917O2+ C10H15O8O2 → C19H28O12	3E-12	
708. C919O2+ C10H15O8O2 → C19H28O13	3E-12	
709. C914O2+ C10H15O8O2 → C19H28O13	3E-12	
710. C916O2+ C10H15O8O2 → C19H28O13	3E-12	
711. C88CO3+ C10H15O8O2 → C19H28O13	3E-12	
712. C87CO3+ C10H15O8O2 → C19H28O14	3E-12	
713. C822CO3+ C10H15O8O2 → C19H28O12	3E-12	
714. NLMKAO2+ C10H15O8O2 → C19H29O11NO3	3E-12	
715. C85O2 + C10H15O8O2 → C18H26O11	3E-12	
716. C89O2 + C10H15O8O2 → C18H26O11	3E-12	
717. C86O2 + C10H15O8O2 → C18H26O12	3E-12	
718. C811O2 + C10H15O8O2 → C18H26O12	3E-12	
719. C810O2 + C10H15O8O2 → C18H26O12	3E-12	
720. C812O2 + C10H15O8O2 → C18H26O13	3E-12	
721. C813O2 + C10H15O8O2 → C18H26O11	3E-12	
722. C729CO3 + C10H15O8O2 → C18H26O12	3E-12	
723. C816O2 + C10H15O8O2 → C18H26O11	3E-12	
724. C817O2 + C10H15O8O2 → C18H26O12	3E-12	
725. C826O2 + C10H15O8O2 → C18H26O13	3E-12	
726. C822O2 + C10H15O8O2 → C18H26O11	3E-12	
727. C818O2 + C10H15O8O2 → C18H26O13	3E-12	
728. C823O2 + C10H15O8O2 → C18H26O12	3E-12	
729. C819O2 + C10H15O8O2 → C18H26O13	3E-12	
730. C727CO3 + C10H15O8O2 → C18H26O13	3E-12	
731. C731CO3 + C10H15O8O2 → C18H26O13	3E-12	
732. C824O2 + C10H15O8O2 → C18H26O12	3E-12	
733. C820O2 + C10H15O8O2 → C18H26O14	3E-12	

734. C825O2 + C10H15O8O2 → C18H26O13	3E-12	
735. C821O2 + C10H15O8O2 → C18H26O13	3E-12	
736. C732CO3 + C10H15O8O2 → C18H26O14	3E-12	
737. C8BCO2 + C10H15O8O2 → C18H26O10	3E-12	
738. C88O2 + C10H15O8O2 → C18H26O12	3E-12	
739. C718CO3 + C10H15O8O2 → C18H26O13	3E-12	
740. C87O2 + C10H15O8O2 → C18H26O13	3E-12	
741. C721CO3 + C10H15O8O2 → C18H26O13	3E-12	
742. NC826O2 + C10H15O8O2 → C18H27O12NO3	3E-12	
743. APINAO2 + C10H15O9O2 → C20H30O12	5E-12	
744. APINBO2 + C10H15O9O2 → C20H30O12	5E-12	
745. APINCO2 + C10H15O9O2 → C20H30O12	5E-12	
746. C107O2 + C10H15O9O2 → C20H30O13	5E-12	
747. C109O2 + C10H15O9O2 → C20H30O13	5E-12	
748. C106O2 + C10H15O9O2 → C20H30O14	5E-12	
749. C920CO3 + C10H15O9O2 → C20H30O14	5E-12	
750. C108O2 + C10H15O9O2 → C20H30O14	5E-12	
751. PINALO2 + C10H15O9O2 → C20H30O13	5E-12	
752. C96CO3 + C10H15O9O2 → C20H30O13	5E-12	
753. C923CO3 + C10H15O9O2 → C20H30O13	5E-12	
754. LIMAO2 + C10H15O9O2 → C20H30O12	5E-12	
755. LIMALBO2 + C10H15O9O2 → C20H30O13	5E-12	
756. LIMCO2 + C10H15O9O2 → C20H30O12	5E-12	
757. LIMALO2 + C10H15O9O2 → C20H30O13	5E-12	
758. LIMBO2 + C10H15O9O2 → C20H30O12	5E-12	
759. LIMALAO2 + C10H15O9O2 → C20H30O13	5E-12	
760. BPINAO2 + C10H15O9O2 → C20H30O12	5E-12	
761. BPINBO2 + C10H15O9O2 → C20H30O12	5E-12	
762. BPINCO2 + C10H15O9O2 → C20H30O12	5E-12	
763. C918CO3 + C10H15O9O2 → C20H30O13	5E-12	
764. NAPINAO2 + C10H15O9O2 → C20H31O11NO3	5E-12	HOM dimers containing NO_3
765. NAPINBO2 + C10H15O9O2 → C20H31O11NO3	5E-12	HOM dimers containing NO_3
766. NC102O2 + C10H15O9O2 → C20H31O12NO3	5E-12	HOM dimers containing NO_3
767. NC101O2 + C10H15O9O2 → C20H31O12NO3	5E-12	HOM dimers containing NO_3
768. NLIMO2 + C10H15O9O2 → C20H31O11NO3	5E-12	HOM dimers containing NO_3
769. NLIMALO2 + C10H15O9O2 → C20H31O12NO3	5E-12	HOM dimers containing NO_3
770. NC91CO3 + C10H15O9O2 → C20H31O12NO3	5E-12	HOM dimers containing NO_3
771. NBPINAO2 + C10H15O9O2 → C20H31O11NO3	5E-12	HOM dimers containing NO_3
772. NBPINBO2 + C10H15O9O2 → C20H31O11NO3	5E-12	HOM dimers containing NO_3
773. C96O2 + C10H15O9O2 → C19H28O12	5E-12	
774. C89CO3 + C10H15O9O2 → C19H28O13	5E-12	
775. C920O2 + C10H15O9O2 → C19H28O13	5E-12	
776. C97O2 + C10H15O9O2 → C19H28O13	5E-12	
777. C85CO3 + C10H15O9O2 → C19H28O13	5E-12	
778. C811CO3 + C10H15O9O2 → C19H28O14	5E-12	
779. C921O2 + C10H15O9O2 → C19H28O14	5E-12	
780. C98O2 + C10H15O9O2 → C19H28O14	5E-12	
781. C922O2+ C10H15O9O2 → C19H28O15	5E-12	
782. C923O2 + C10H15O9O2 → C19H28O12	5E-12	
783. C924O2 + C10H15O9O2 → C19H28O13	5E-12	
784. C816CO3 + C10H15O9O2 → C19H28O13	5E-12	
785. NORLIMO2 + C10H15O9O2 → C19H28O14	5E-12	
786. LMKAO2 + C10H15O9O2 → C19H28O13	5E-12	
787. LMKBO2 + C10H15O9O2 → C19H28O13	5E-12	
788. C926O2 + C10H15O9O2 → C19H28O14	5E-12	
789. C817CO3 + C10H15O9O2 → C19H28O14	5E-12	
790. LMLKAO2 + C10H15O9O2 → C19H28O14	5E-12	
791. LMLKBO2 + C10H15O9O2 → C19H28O14	5E-12	
792. C823CO3 + C10H15O9O2 → C19H28O14	5E-12	
793. C925O2 + C10H15O9O2 → C19H28O15	5E-12	
794. NOPINAO2+ C10H15O9O2 → C19H28O12	5E-12	
795. NOPINBO2+ C10H15O9O2 → C19H28O12	5E-12	
796. NOPINCO2+ C10H15O9O2 → C19H28O12	5E-12	
797. NOPINDO2+ C10H15O9O2 → C19H28O12	5E-12	
798. C918O2+ C10H15O9O2 → C19H28O13	5E-12	
799. C9DCO2+ C10H15O9O2 → C19H28O13	5E-12	
800. C915O2+ C10H15O9O2 → C19H28O13	5E-12	
801. C917O2+ C10H15O9O2 → C19H28O13	5E-12	
802. C919O2+ C10H15O9O2 → C19H28O14	5E-12	
803. C914O2+ C10H15O9O2 → C19H28O14	5E-12	
804. C916O2+ C10H15O9O2 → C19H28O14	5E-12	
805. C88CO3+ C10H15O9O2 → C19H28O14	5E-12	

806. C87CO3+ C10H15O9O2 → C19H28O15	5E-12	
807. C822CO3+ C10H15O9O2 → C19H28O13	5E-12	
808. NLMKAO2+ C10H15O9O2 → C19H29O12NO3	5E-12	
809. C85O2 + C10H15O9O2 → C18H26O12	5E-12	
810. C89O2 + C10H15O9O2 → C18H26O12	5E-12	
811. C86O2 + C10H15O9O2 → C18H26O13	5E-12	
812. C811O2 + C10H15O9O2 → C18H26O13	5E-12	
813. C810O2 + C10H15O9O2 → C18H26O13	5E-12	
814. C812O2 + C10H15O9O2 → C18H26O14	5E-12	
815. C813O2 + C10H15O9O2 → C18H26O12	5E-12	
816. C729CO3 + C10H15O9O2 → C18H26O13	5E-12	
817. C816O2 + C10H15O9O2 → C18H26O12	5E-12	
818. C817O2 + C10H15O9O2 → C18H26O13	5E-12	
819. C826O2 + C10H15O9O2 → C18H26O14	5E-12	
820. C822O2 + C10H15O9O2 → C18H26O12	5E-12	
821. C818O2 + C10H15O9O2 → C18H26O14	5E-12	
822. C823O2 + C10H15O9O2 → C18H26O13	5E-12	
823. C819O2 + C10H15O9O2 → C18H26O14	5E-12	
824. C727CO3 + C10H15O9O2 → C18H26O14	5E-12	
825. C731CO3 + C10H15O9O2 → C18H26O14	5E-12	
826. C824O2 + C10H15O9O2 → C18H26O13	5E-12	
827. C820O2 + C10H15O9O2 → C18H26O15	5E-12	
828. C825O2 + C10H15O9O2 → C18H26O14	5E-12	
829. C821O2 + C10H15O9O2 → C18H26O14	5E-12	
830. C732CO3 + C10H15O9O2 → C18H26O15	5E-12	
831. C8BCO2 + C10H15O9O2 → C18H26O11	5E-12	
832. C88O2 + C10H15O9O2 → C18H26O13	5E-12	
833. C718CO3 + C10H15O9O2 → C18H26O14	5E-12	
834. C87O2 + C10H15O9O2 → C18H26O14	5E-12	
835. C721CO3 + C10H15O9O2 → C18H26O14	5E-12	
836. NC826O2 + C10H15O9O2 → C18H27O13NO3	5E-12	
837. APINAO2 + C10H15O10O2 → C20H30O13	5E-12	
838. APINBO2 + C10H15O10O2 → C20H30O13	5E-12	
839. APINCO2 + C10H15O10O2 → C20H30O13	5E-12	
840. C107O2 + C10H15O10O2 → C20H30O14	5E-12	
841. C109O2 + C10H15O10O2 → C20H30O14	5E-12	
842. C106O2 + C10H15O10O2 → C20H30O15	5E-12	
843. C920CO3 + C10H15O10O2 → C20H30O15	5E-12	
844. C108O2 + C10H15O10O2 → C20H30O15	5E-12	
845. PINALO2 + C10H15O10O2 → C20H30O14	5E-12	
846. C96CO3 + C10H15O10O2 → C20H30O14	5E-12	
847. C923CO3 + C10H15O10O2 → C20H30O14	5E-12	
848. LIMAO2 + C10H15O10O2 → C20H30O13	5E-12	
849. LIMALBO2 + C10H15O10O2 → C20H30O14	5E-12	
850. LIMCO2 + C10H15O10O2 → C20H30O13	5E-12	
851. LIMALO2 + C10H15O10O2 → C20H30O14	5E-12	
852. LIMBO2 + C10H15O10O2 → C20H30O13	5E-12	
853. LIMALAO2 + C10H15O10O2 → C20H30O14	5E-12	
854. BPINAO2 + C10H15O10O2 → C20H30O13	5E-12	
855. BPINBO2 + C10H15O10O2 → C20H30O13	5E-12	
856. BPINCO2 + C10H15O10O2 → C20H30O13	5E-12	
857. C918CO3 + C10H15O10O2 → C20H30O14	5E-12	
858. NAPINAO2 + C10H15O10O2 → C20H31O12NO3	5E-12	HOM dimers containing NO_3
859. NAPINBO2 + C10H15O10O2 → C20H31O12NO3	5E-12	HOM dimers containing NO_3
860. NC102O2 + C10H15O10O2 → C20H31O13NO3	5E-12	HOM dimers containing NO_3
861. NC101O2 + C10H15O10O2 → C20H31O13NO3	5E-12	HOM dimers containing NO_3
862. NLIMO2 + C10H15O10O2 → C20H31O12NO3	5E-12	HOM dimers containing NO_3
863. NLIMALO2 + C10H15O10O2 → C20H31O13NO3	5E-12	HOM dimers containing NO_3
864. NC91CO3 + C10H15O10O2 → C20H31O13NO3	5E-12	HOM dimers containing NO_3
865. NBPINAO2 + C10H15O10O2 → C20H31O12NO3	5E-12	HOM dimers containing NO_3
866. NBPINBO2 + C10H15O10O2 → C20H31O12NO3	5E-12	HOM dimers containing NO_3
867. C96O2 + C10H15O10O2 → C19H28O13	5E-12	
868. C89CO3 + C10H15O10O2 → C19H28O14	5E-12	
869. C920O2 + C10H15O10O2 → C19H28O14	5E-12	
870. C97O2 + C10H15O10O2 → C19H28O14	5E-12	
871. C85CO3 + C10H15O10O2 → C19H28O14	5E-12	
872. C811CO3 + C10H15O10O2 → C19H28O15	5E-12	
873. C921O2 + C10H15O10O2 → C19H28O15	5E-12	
874. C98O2 + C10H15O10O2 → C19H28O15	5E-12	
875. C922O2+ C10H15O10O2 → C19H28O16	5E-12	
876. C923O2 + C10H15O10O2 → C19H28O13	5E-12	

877. C924O2 + C10H15O10O2 → C19H28O14	5E-12
878. C816CO3 + C10H15O10O2 → C19H28O14	5E-12
879. NORLIMO2 + C10H15O10O2 → C19H28O15	5E-12
880. LMKAO2 + C10H15O10O2 → C19H28O14	5E-12
881. LMKBO2 + C10H15O10O2 → C19H28O14	5E-12
882. C926O2 + C10H15O10O2 → C19H28O15	5E-12
883. C817CO3 + C10H15O10O2 → C19H28O15	5E-12
884. LMLKAO2 + C10H15O10O2 → C19H28O15	5E-12
885. LMLKBO2 + C10H15O10O2 → C19H28O15	5E-12
886. C823CO3 + C10H15O10O2 → C19H28O15	5E-12
887. C925O2 + C10H15O10O2 → C19H28O16	5E-12
888. NOPINAO2+ C10H15O10O2 → C19H28O13	5E-12
889. NOPINBO2+ C10H15O10O2 → C19H28O13	5E-12
890. NOPINCO2+ C10H15O10O2 → C19H28O13	5E-12
891. NOPINDO2+ C10H15O10O2 → C19H28O13	5E-12
892. C918O2+ C10H15O10O2 → C19H28O14	5E-12
893. C9DCO2+ C10H15O10O2 → C19H28O14	5E-12
894. C915O2+ C10H15O10O2 → C19H28O14	5E-12
895. C917O2+ C10H15O10O2 → C19H28O14	5E-12
896. C919O2+ C10H15O10O2 → C19H28O15	5E-12
897. C914O2+ C10H15O10O2 → C19H28O15	5E-12
898. C916O2+ C10H15O10O2 → C19H28O15	5E-12
899. C88CO3+ C10H15O10O2 → C19H28O15	5E-12
900. C87CO3+ C10H15O10O2 → C19H28O16	5E-12
901. C822CO3+ C10H15O10O2 → C19H28O14	5E-12
902. NLMKAO2+ C10H15O10O2 → C19H29O13NO3	5E-12
903. C85O2 + C10H15O10O2 → C18H26O13	5E-12
904. C89O2 + C10H15O10O2 → C18H26O13	5E-12
905. C86O2 + C10H15O10O2 → C18H26O14	5E-12
906. C811O2 + C10H15O10O2 → C18H26O14	5E-12
907. C810O2 + C10H15O10O2 → C18H26O14	5E-12
908. C812O2 + C10H15O10O2 → C18H26O15	5E-12
909. C813O2 + C10H15O10O2 → C18H26O13	5E-12
910. C729CO3 + C10H15O10O2 → C18H26O14	5E-12
911. C816O2 + C10H15O10O2 → C18H26O13	5E-12
912. C817O2 + C10H15O10O2 → C18H26O14	5E-12
913. C826O2 + C10H15O10O2 → C18H26O15	5E-12
914. C822O2 + C10H15O10O2 → C18H26O13	5E-12
915. C818O2 + C10H15O10O2 → C18H26O15	5E-12
916. C823O2 + C10H15O10O2 → C18H26O14	5E-12
917. C819O2 + C10H15O10O2 → C18H26O15	5E-12
918. C727CO3 + C10H15O10O2 → C18H26O15	5E-12
919. C731CO3 + C10H15O10O2 → C18H26O15	5E-12
920. C824O2 + C10H15O10O2 → C18H26O14	5E-12
921. C820O2 + C10H15O10O2 → C18H26O16	5E-12
922. C825O2 + C10H15O10O2 → C18H26O15	5E-12
923. C821O2 + C10H15O10O2 → C18H26O15	5E-12
924. C732CO3 + C10H15O10O2 → C18H26O16	5E-12
925. C8BCO2 + C10H15O10O2 → C18H26O12	5E-12
926. C88O2 + C10H15O10O2 → C18H26O14	5E-12
927. C718CO3 + C10H15O10O2 → C18H26O15	5E-12
928. C87O2 + C10H15O10O2 → C18H26O15	5E-12
929. C721CO3 + C10H15O10O2 → C18H26O15	5E-12
930. NC826O2 + C10H15O10O2 → C18H27O14NO3	5E-12
931. APINAO2 + C10H15O11O2 → C20H30O14	5E-12
932. APINBO2 + C10H15O11O2 → C20H30O14	5E-12
933. APINCO2 + C10H15O11O2 → C20H30O14	5E-12
934. C107O2 + C10H15O11O2 → C20H30O15	5E-12
935. C109O2 + C10H15O11O2 → C20H30O15	5E-12
936. C106O2 + C10H15O11O2 → C20H30O16	5E-12
937. C920CO3 + C10H15O11O2 → C20H30O16	5E-12
938. C108O2 + C10H15O11O2 → C20H30O16	5E-12
939. PINALO2 + C10H15O11O2 → C20H30O15	5E-12
940. C96CO3 + C10H15O11O2 → C20H30O15	5E-12
941. C923CO3 + C10H15O11O2 → C20H30O15	5E-12
942. LIMAO2 + C10H15O11O2 → C20H30O14	5E-12
943. LIMALBO2 + C10H15O11O2 → C20H30O15	5E-12
944. LIMCO2 + C10H15O11O2 → C20H30O14	5E-12
945. LIMALO2 + C10H15O11O2 → C20H30O15	5E-12
946. LIMBO2 + C10H15O11O2 → C20H30O14	5E-12
947. LIMALAO2 + C10H15O11O2 → C20H30O15	5E-12
948. BPINAO2 + C10H15O11O2 → C20H30O14	5E-12

949. BPINBO2 + C10H15O11O2 → C20H30O14	5E-12	
950. BPINCO2 + C10H15O11O2 → C20H30O14	5E-12	
951. C918CO3 + C10H15O11O2 → C20H30O15	5E-12	
952. NAPINAO2 + C10H15O11O2 → C20H31O13NO3	5E-12	HOM dimers containing NO_3
953. NAPINBO2 + C10H15O11O2 → C20H31O13NO3	5E-12	HOM dimers containing NO_3
954. NC102O2 + C10H15O11O2 → C20H31O14NO3	5E-12	HOM dimers containing NO_3
955. NC101O2 + C10H15O11O2 → C20H31O14NO3	5E-12	HOM dimers containing NO_3
956. NLIMO2 + C10H15O11O2 → C20H31O13NO3	5E-12	HOM dimers containing NO_3
957. NLIMALO2 + C10H15O11O2 → C20H31O14NO3	5E-12	HOM dimers containing NO_3
958. NC91CO3 + C10H15O11O2 → C20H31O14NO3	5E-12	HOM dimers containing NO_3
959. NBPINAO2 + C10H15O11O2 → C20H31O13NO3	5E-12	HOM dimers containing NO_3
960. NBPINBO2 + C10H15O11O2 → C20H31O13NO3	5E-12	HOM dimers containing NO_3

961. C96O2 + C10H15O11O2 → C19H28O14	5E-12
962. C89CO3 + C10H15O11O2 → C19H28O15	5E-12
963. C920O2 + C10H15O11O2 → C19H28O15	5E-12
964. C97O2 + C10H15O11O2 → C19H28O15	5E-12
965. C85CO3 + C10H15O11O2 → C19H28O15	5E-12
966. C811CO3 + C10H15O11O2 → C19H28O16	5E-12
967. C921O2 + C10H15O11O2 → C19H28O16	5E-12
968. C98O2 + C10H15O11O2 → C19H28O16	5E-12
969. C922O2+ C10H15O11O2 → C19H28O17	5E-12
970. C923O2 + C10H15O11O2 → C19H28O14	5E-12
971. C924O2 + C10H15O11O2 → C19H28O15	5E-12
972. C816CO3 + C10H15O11O2 → C19H28O15	5E-12
973. NORLIMO2 + C10H15O11O2 → C19H28O16	5E-12
974. LMKAO2 + C10H15O11O2 → C19H28O15	5E-12
975. LMKBO2 + C10H15O11O2 → C19H28O15	5E-12
976. C926O2 + C10H15O11O2 → C19H28O16	5E-12
977. C817CO3 + C10H15O11O2 → C19H28O16	5E-12
978. LMLKAO2 + C10H15O11O2 → C19H28O16	5E-12
979. LMLKBO2 + C10H15O11O2 → C19H28O16	5E-12
980. C823CO3 + C10H15O11O2 → C19H28O16	5E-12
981. C925O2 + C10H15O11O2 → C19H28O17	5E-12
982. NOPINAO2+ C10H15O11O2 → C19H28O14	5E-12
983. NOPINBO2+ C10H15O11O2 → C19H28O14	5E-12
984. NOPINCO2+ C10H15O11O2 → C19H28O14	5E-12
985. NOPINDO2+ C10H15O11O2 → C19H28O14	5E-12
986. C918O2+ C10H15O11O2 → C19H28O15	5E-12
987. C9DCO2+ C10H15O11O2 → C19H28O15	5E-12
988. C915O2+ C10H15O11O2 → C19H28O15	5E-12
989. C917O2+ C10H15O11O2 → C19H28O15	5E-12
990. C919O2+ C10H15O11O2 → C19H28O16	5E-12
991. C914O2+ C10H15O11O2 → C19H28O16	5E-12
992. C916O2+ C10H15O11O2 → C19H28O16	5E-12
993. C88CO3+ C10H15O11O2 → C19H28O16	5E-12
994. C87CO3+ C10H15O11O2 → C19H28O17	5E-12
995. C822CO3+ C10H15O11O2 → C19H28O15	5E-12
996. NLMKAO2+ C10H15O11O2 → C19H29O14NO3	5E-12

997. C85O2 + C10H15O11O2 → C18H26O14	5E-12
998. C89O2 + C10H15O11O2 → C18H26O14	5E-12
999. C86O2 + C10H15O11O2 → C18H26O15	5E-12
1000. C811O2 + C10H15O11O2 → C18H26O15	5E-12
1001. C810O2 + C10H15O11O2 → C18H26O15	5E-12
1002. C812O2 + C10H15O11O2 → C18H26O16	5E-12
1003. C813O2 + C10H15O11O2 → C18H26O14	5E-12
1004. C729CO3 + C10H15O11O2 → C18H26O15	5E-12
1005. C816O2 + C10H15O11O2 → C18H26O14	5E-12
1006. C817O2 + C10H15O11O2 → C18H26O15	5E-12
1007. C826O2 + C10H15O11O2 → C18H26O16	5E-12
1008. C822O2 + C10H15O11O2 → C18H26O14	5E-12
1009. C818O2 + C10H15O11O2 → C18H26O16	5E-12
1010. C823O2 + C10H15O11O2 → C18H26O15	5E-12
1011. C819O2 + C10H15O11O2 → C18H26O16	5E-12
1012. C727CO3 + C10H15O11O2 → C18H26O16	5E-12
1013. C731CO3 + C10H15O11O2 → C18H26O16	5E-12
1014. C824O2 + C10H15O11O2 → C18H26O15	5E-12
1015. C820O2 + C10H15O11O2 → C18H26O17	5E-12
1016. C825O2 + C10H15O11O2 → C18H26O16	5E-12
1017. C821O2 + C10H15O11O2 → C18H26O16	5E-12
1018. C732CO3 + C10H15O11O2 → C18H26O17	5E-12
1019. C8BCO2 + C10H15O11O2 → C18H26O13	5E-12
1020. C88O2 + C10H15O11O2 → C18H26O15	5E-12

1021. C718CO3 + C10H15O11O2 → C18H26O16	5E-12	
1022. C87O2 + C10H15O11O2 → C18H26O16	5E-12	
1023. C721CO3 + C10H15O11O2 → C18H26O16	5E-12	
1024. NC826O2 + C10H15O11O2 → C18H27O15NO3	5E-12	

1025. APINAO2 + C10H15O12O2 → C20H30O15	5E-12	
1026. APINBO2 + C10H15O12O2 → C20H30O15	5E-12	
1027. APINCO2 + C10H15O12O2 → C20H30O15	5E-12	
1028. C107O2 + C10H15O12O2 → C20H30O16	5E-12	
1029. C109O2 + C10H15O12O2 → C20H30O16	5E-12	
1030. C106O2 + C10H15O12O2 → C20H30O17	5E-12	
1031. C920CO3 + C10H15O12O2 → C20H30O17	5E-12	
1032. C108O2 + C10H15O12O2 → C20H30O17	5E-12	
1033. PINALO2 + C10H15O12O2 → C20H30O16	5E-12	
1034. C96CO3 + C10H15O12O2 → C20H30O16	5E-12	
1035. C923CO3 + C10H15O12O2 → C20H30O16	5E-12	
1036. LIMAO2 + C10H15O12O2 → C20H30O15	5E-12	
1037. LIMALBO2 + C10H15O12O2 → C20H30O16	5E-12	
1038. LIMCO2 + C10H15O21O2 → C20H30O15	5E-12	
1039. LIMALO2 + C10H15O12O2 → C20H30O16	5E-12	
1040. LIMBO2 + C10H15O12O2 → C20H30O15	5E-12	
1041. LIMALAO2 + C10H15O12O2 → C20H30O16	5E-12	
1042. BPINAO2 + C10H15O12O2 → C20H30O15	5E-12	
1043. BPINBO2 + C10H15O12O2 → C20H30O15	5E-12	
1044. BPINCO2 + C10H15O12O2 → C20H30O15	5E-12	
1045. C918CO3 + C10H15O12O2 → C20H30O16	5E-12	
1046. NAPINAO2 + C10H15O12O2 → C20H31O14NO3	5E-12	HOM dimers containing NO_3
1047. NAPINBO2 + C10H15O12O2 → C20H31O14NO3	5E-12	HOM dimers containing NO_3
1048. NC102O2 + C10H15O12O2 → C20H31O15NO3	5E-12	HOM dimers containing NO_3
1049. NC101O2 + C10H15O12O2 → C20H31O15NO3	5E-12	HOM dimers containing NO_3
1050. NLIMO2 + C10H15O12O2 → C20H31O14NO3	5E-12	HOM dimers containing NO_3
1051. NLIMALO2 + C10H15O12O2 → C20H31O15NO3	5E-12	HOM dimers containing NO_3
1052. NC91CO3 + C10H15O12O2 → C20H31O15NO3	5E-12	HOM dimers containing NO_3
1053. NBPINAO2 + C10H15O12O2 → C20H31O14NO3	5E-12	HOM dimers containing NO_3
1054. NBPINBO2 + C10H15O12O2 → C20H31O14NO3	5E-12	HOM dimers containing NO_3

1055. C96O2 + C10H15O12O2 → C19H28O15	5E-12	
1056. C89CO3 + C10H15O12O2 → C19H28O16	5E-12	
1057. C920O2 + C10H15O12O2 → C19H28O16	5E-12	
1058. C97O2 + C10H15O12O2 → C19H28O16	5E-12	
1059. C85CO3 + C10H15O12O2 → C19H28O16	5E-12	
1060. C811CO3 + C10H15O12O2 → C19H28O17	5E-12	
1061. C921O2 + C10H15O12O2 → C19H28O17	5E-12	
1062. C98O2 + C10H15O12O2 → C19H28O17	5E-12	
1063. C922O2+ C10H15O12O2 → C19H28O18	5E-12	
1064. C923O2 + C10H15O12O2 → C19H28O15	5E-12	
1065. C924O2 + C10H15O12O2 → C19H28O16	5E-12	
1066. C816CO3 + C10H15O12O2 → C19H28O16	5E-12	
1067. NORLIMO2 + C10H15O12O2 → C19H28O17	5E-12	
1068. LMKAO2 + C10H15O12O2 → C19H28O16	5E-12	
1069. LMKBO2 + C10H15O12O2 → C19H28O16	5E-12	
1070. C926O2 + C10H15O12O2 → C19H28O17	5E-12	
1071. C817CO3 + C10H15O12O2 → C19H28O17	5E-12	
1072. LMLKAO2 + C10H15O12O2 → C19H28O17	5E-12	
1073. LMLKBO2 + C10H15O12O2 → C19H28O17	5E-12	
1074. C823CO3 + C10H15O12O2 → C19H28O17	5E-12	
1075. C925O2 + C10H15O12O2 → C19H28O18	5E-12	
1076. NOPINAO2+ C10H15O12O2 → C19H28O15	5E-12	
1077. NOPINBO2+ C10H15O12O2 → C19H28O15	5E-12	
1078. NOPINCO2+ C10H15O12O2 → C19H28O15	5E-12	
1079. NOPINDO2+ C10H15O12O2 → C19H28O15	5E-12	
1080. C918O2+ C10H15O12O2 → C19H28O16	5E-12	
1081. C9DCO2+ C10H15O12O2 → C19H28O16	5E-12	
1082. C915O2+ C10H15O12O2 → C19H28O16	5E-12	
1083. C917O2+ C10H15O12O2 → C19H28O16	5E-12	
1084. C919O2+ C10H15O12O2 → C19H28O17	5E-12	
1085. C914O2+ C10H15O12O2 → C19H28O17	5E-12	
1086. C916O2+ C10H15O12O2 → C19H28O17	5E-12	
1087. C88CO3+ C10H15O12O2 → C19H28O17	5E-12	
1088. C87CO3+ C10H15O12O2 → C19H28O18	5E-12	
1089. C822CO3+ C10H15O12O2 → C19H28O16	5E-12	
1090. NLMKAO2+ C10H15O12O2 → C19H29O15NO3	5E-12	

1091. C85O2 + C10H15O12O2 → C18H26O15	5E-12	

1092. C89O2 + C10H15O12O2 → C18H26O15 5E-12
1093. C86O2 + C10H15O12O2 → C18H26O16 5E-12
1094. C811O2 + C10H15O12O2 → C18H26O16 5E-12
1095. C810O2 + C10H15O12O2 → C18H26O16 5E-12
1096. C812O2 + C10H15O12O2 → C18H26O17 5E-12
1097. C813O2 + C10H15O12O2 → C18H26O15 5E-12
1098. C729CO3 + C10H15O12O2 → C18H26O16 5E-12
1099. C816O2 + C10H15O12O2 → C18H26O15 5E-12
1100. C817O2 + C10H15O12O2 → C18H26O16 5E-12
1101. C826O2 + C10H15O12O2 → C18H26O17 5E-12
1102. C822O2 + C10H15O12O2 → C18H26O15 5E-12
1103. C818O2 + C10H15O12O2 → C18H26O17 5E-12
1104. C823O2 + C10H15O12O2 → C18H26O16 5E-12
1105. C819O2 + C10H15O12O2 → C18H26O17 5E-12
1106. C727CO3 + C10H15O12O2 → C18H26O17 5E-12
1107. C731CO3 + C10H15O12O2 → C18H26O17 5E-12
1108. C824O2 + C10H15O12O2 → C18H26O16 5E-12
1109. C820O2 + C10H15O12O2 → C18H26O18 5E-12
1110. C825O2 + C10H15O12O2 → C18H26O17 5E-12
1111. C821O2 + C10H15O12O2 → C18H26O17 5E-12
1112. C732CO3 + C10H15O12O2 → C18H26O18 5E-12
1113. C8BCO2 + C10H15O12O2 → C18H26O14 5E-12
1114. C88O2 + C10H15O12O2 → C18H26O16 5E-12
1115. C718CO3 + C10H15O12O2 → C18H26O17 5E-12
1116. C87O2 + C10H15O12O2 → C18H26O17 5E-12
1117. C721CO3 + C10H15O12O2 → C18H26O17 5E-12
1118. NC826O2 + C10H15O12O2 → C18H27O16NO3 5E-12

Autoxidation peroxy radicals reacting with other peroxy radicals with different branching ratios for a, b and c:

$$R_1O_2 + R_2O_2 \rightarrow R_1C{=}O + R_2OH \quad \textbf{(a)}$$
$$\rightarrow R_1OH + R_2C{=}O \quad \textbf{(b)}$$
$$\rightarrow R_1O + R_2O + O_2 \quad \textbf{(c)}$$

1119. C10H15O2O2 → C10H14O3 1E-12*0.2*RO2 RO2 = ∑ RO_2 in MCM
1120. C10H15O2O2 → C10H15O2O 1E-12*0.6*RO2 RO2 = ∑ RO_2 in MCM
1121. C10H15O2O2 → C10H16O3 1E-12*0.2*RO2 RO2 = ∑ RO_2 in MCM

1122. C10H15O3O2 → C10H14O4 5E-12*0.2*RO2 RO2 = ∑ RO_2 in MCM
1123. C10H15O3O2 → C10H15O3O 5E-12*0.6*RO2 RO2 = ∑ RO_2 in MCM
1124. C10H15O3O2 → C10H16O4iso1 5E-12*0.2*RO2 RO2 = ∑ RO_2 in MCM

1125. C10H15O4O2 → C10H14O5 5E-12*0.2*RO2 RO2 = ∑ RO_2 in MCM
1126. C10H15O4O2 → C10H15O4O 5E-12*0.6*RO2 RO2 = ∑ RO_2 in MCM
1127. C10H15O4O2 → C10H16O5iso1 5E-12*0.2*RO2 RO2 = ∑ RO_2 in MCM

1128. C10H15O5O2 → C10H14O6 5E-12*0.2*RO2 RO2 = ∑ RO_2 in MCM
1129. C10H15O5O2 → C10H15O5O 5E-12*0.6*RO2 RO2 = ∑ RO_2 in MCM
1130. C10H15O5O2 → C10H16O6iso1 5E-12*0.2*RO2 RO2 = ∑ RO_2 in MCM

1131. C10H15O6O2 → C10H14O7 7E-12*0.4*RO2 RO2 = ∑ RO_2 in MCM
1132. C10H15O6O2 → C10H15O6O 7E-12*0.4*RO2 RO2 = ∑ RO_2 in MCM
1133. C10H15O6O2 → C10H16O7iso1 7E-12*0.2*RO2 RO2 = ∑ RO_2 in MCM

1134. C10H15O7O2 → C10H14O8 8E-12*0.2*RO2 RO2 = ∑ RO_2 in MCM
1135. C10H15O7O2 → C10H15O7O 8E-12*0.4*RO2 RO2 = ∑ RO_2 in MCM
1136. C10H15O7O2 → C10H16O8iso1 8E-12*0.4*RO2 RO2 = ∑ RO_2 in MCM

1137. C10H15O8O2 → C10H14O9 9E-12*0.4*RO2 RO2 = ∑ RO_2 in MCM
1138. C10H15O8O2 → C10H15O8O 9E-12*0.2*RO2 RO2 = ∑ RO_2 in MCM
1139. C10H15O8O2 → C10H16O9iso1 9E-12*0.4*RO2 RO2 = ∑ RO_2 in MCM

1140. C10H15O9O2 → C10H14O10 1E-11*0.3*RO2 RO2 = ∑ RO_2 in MCM
1141. C10H15O9O2 → C10H15O9O 1E-11*0.2*RO2 RO2 = ∑ RO_2 in MCM
1142. C10H15O9O2 → C10H16O10 1E-11*0.5*RO2 RO2 = ∑ RO_2 in MCM

1143. C10H15O10O2 → C10H14O11 1E-11*0.5*RO2 RO2 = ∑ RO_2 in MCM
1144. C10H15O10O2 → C10H15O10O 1E-11*0.2*RO2 RO2 = ∑ RO_2 in MCM
1145. C10H15O10O2 → C10H16O11 1E-11*0.3*RO2 RO2 = ∑ RO_2 in MCM

1146. C10H15O11O2 → C10H14O12 1E-11*0.4*RO2 RO2 = ∑ RO_2 in MCM
1147. C10H15O11O2 → C10H15O11O 1E-11*0.2*RO2 RO2 = ∑ RO_2 in MCM
1148. C10H15O11O2 → C10H16O12 1E-11*0.4*RO2 RO2 = ∑ RO_2 in MCM

1149. C10H15O12O2 → C10H14O13 1E-11*0.5*RO2 RO2 = ∑ RO_2 in MCM
1150. C10H15O12O2 → C10H15O12O 1E-11*0.0*RO2 RO2 = ∑ RO_2 in MCM

1151. C10H15O12O2 → C10H16O13	1E-11*0.5*RO2	RO2 = ∑ RO_2 in MCM

1152. C107O2 → C10H15O2O	9.4E-14*RO2*0.1	To PRAM, RO2 = ∑ RO_2 in MCM
1153. C107O2 → C107O	9.4E-14*RO2*0.63	To MCM, RO2 = ∑ RO_2 in MCM
1154. C107O2 → C107OH	9.4E-14*RO2*0.27	To MCM, RO2 = ∑ RO_2 in MCM

Peroxy radical autoxidation and HOM formation after OH oxidation of monoterpenes (R1155-R1773)

Autoxidation reaction rates (H-shifts + O_2)

1155. C10H17O3O2 → C10H17O5O2	2E17*EXP(-12077/T)	Unit s^{-1}
1156. C10H17O4O2 → C10H17O6O2	6E16*EXP(-12077/T)	Unit s^{-1}
1157. C10H17O5O2 → C10H17O7O2	6E16*EXP(-12077/T)	Unit s^{-1}
1158. C10H17O6O2 → C10H17O8O2	3E16*EXP(-12077/T)	Unit s^{-1}

Isomerization of alkoxy radicals (RO) followed by reaction with O_2 to make new peroxy radicals (RO· →· ROH, · ROH + O_2 → RO_2)

1159. C10H17O3O → C10H17O4O2	KDEC	Unit s^{-1}
1160. C10H17O4O → C10H17O5O2	KDEC	Unit s^{-1}
1161. C10H17O5O → C10H17O6O2	KDEC	Unit s^{-1}
1162. C10H17O6O → C10H17O7O2	KDEC	Unit s^{-1}
1163. C10H17O7O → C10H17O8O2	KDEC	Unit s^{-1}

Peroxy radicals reacting with NO and forming an alkoxy radical and NO_2 (RO_2 + NO → RO + NO_2)

*The rate for the reactions between autoxidation RO_2 and NO was set to the same values used in MCMv3.3.1 (*KRO2NO)

1164. C10H17O3O2 + NO → C10H17O3O + NO2	KRO2NO*1.0
1165. C10H17O4O2 + NO → C10H17O4O + NO2	KRO2NO*0.9
1166. C10H17O5O2 + NO → C10H17O5O + NO2	KRO2NO*0.8
1167. C10H17O6O2 + NO → C10H17O6O + NO2	KRO2NO*0.7
1168. C10H17O7O2 + NO → C10H17O7O + NO2	KRO2NO*0.5

Lumped reactions describing the fragmentation of autoxidation products when they react with NO (RO_2 + NO → RO + NO_2) followed by fast fragmentation of the formed alkoxy radicals).

The fragmentation products are assumed to be the MCM compounds C717O2 (a peroxy radical) and CH3COCH3 (acetone) irrespectively of which autoxidation peroxy radical that react with NO. This is a simplification that violates the mass balance for O atoms but not for the N and C atoms.

1169. C10H17O3O2 + NO → C717O2 + CH3COCH3 + NO2	0.0
1170. C10H17O4O2 + NO → C717O2 + CH3COCH3 + NO2	KRO2NO*0.04
1171. C10H17O5O2 + NO → C717O2 + CH3COCH3 + NO2	KRO2NO*0.08
1172. C10H17O6O2 + NO → C717O2 + CH3COCH3 + NO2	KRO2NO*0.12
1173. C10H17O7O2 + NO → C717O2 + CH3COCH3 + NO2	KRO2NO*0.20
1174. C10H17O8O2 + NO → C717O2 + CH3COCH3 + NO2	KRO2NO*0.40

Peroxy radicals reacting with NO forming monomers with an additional carbonyl group:
RO_2 + NO → RO + NO_2
RO + O_2 → RCHO + HO_2 (Fast)
Net: RO_2 + NO + O_2→ RCHO + NO_2 + HO_2

1175. C10H17O3O2 + NO → C10H16O4iso2 + NO2 + HO2	0.0
1176. C10H17O4O2 + NO → C10H16O5iso2 + NO2 + HO2	KRO2NO*0.042
1177. C10H17O5O2 + NO → C10H16O6iso2 + NO2 + HO2	KRO2NO*0.084
1178. C10H17O6O2 + NO → C10H16O7iso2 + NO2 + HO2	KRO2NO*0.126
1179. C10H17O7O2 + NO → C10H16O8iso2 + NO2 + HO2	KRO2NO*0.21
1180. C10H17O8O2 + NO → C10H16O9iso2 + NO2 + HO2	KRO2NO*0.42

Peroxy radicals reacting with NO forming compounds with a nitrate functional group (RO_2 + NO → RNO_3)

1181. C10H17O3O2 + NO → C10H17O3NO3	0.0
1182. C10H17O4O2 + NO → C10H17O4NO3	KRO2NO*0.018
1183. C10H17O5O2 + NO → C10H17O5NO3	KRO2NO*0.036
1184. C10H17O6O2 + NO → C10H17O6NO3	KRO2NO*0.054
1185. C10H17O7O2 + NO → C10H17O7NO3	KRO2NO*0.090
1186. C10H17O8O2 + NO → C10H17O8NO3	KRO2NO*0.180

Peroxy radicals reacting with HO_2 forming compounds with a hydrogen peroxide functional group (RO_2 + HO_2 → ROOH + O_2)

1187. C10H17O3O2 + HO2 → C10H18O5	KRO2HO2
1188. C10H17O4O2 + HO2 → C10H18O6	KRO2HO2
1189. C10H17O5O2 + HO2 → C10H18O7	KRO2HO2
1190. C10H17O6O2 + HO2 → C10H18O8	KRO2HO2
1191. C10H17O7O2 + HO2 → C10H18O9	KRO2HO2
1192. C10H17O8O2 + HO2 → C10H18O10	KRO2HO2

Peroxy radicals reacting with other peroxy radicals forming dimers (RO_2 + RO_2 → ROOR + O_2)

1193. APINAO2 + C10H17O3O2 → C20H34O6	1E-13
1194. APINBO2 + C10H17O3O2 → C20H34O6	1E-13
1195. APINCO2 + C10H17O3O2 → C20H34O6	1E-13
1196. C107O2 + C10H17O3O2 → C20H34O7	1E-13
1197. C109O2 + C10H17O3O2 → C20H34O7	1E-13
1198. C106O2 + C10H17O3O2 → C20H34O8	1E-13

1199. C920CO3 + C10H17O3O2 → C20H34O8	1E-13	
1200. C108O2 + C10H17O3O2 → C20H34O8	1E-13	
1201. PINALO2 + C10H17O3O2 → C20H34O7	1E-13	
1202. C96CO3 + C10H17O3O2 → C20H34O7	1E-13	
1203. C923CO3 + C10H17O3O2 → C20H34O7	1E-13	
1204. LIMAO2 + C10H17O3O2 → C20H34O6	1E-13	
1205. LIMALBO2 + C10H17O3O2 → C20H34O7	1E-13	
1206. LIMCO2 + C10H17O3O2 → C20H34O6	1E-13	
1207. LIMALO2 + C10H17O3O2 → C20H34O7	1E-13	
1208. LIMBO2 + C10H17O3O2 → C20H34O6	1E-13	
1209. LIMALAO2 + C10H17O3O2 → C20H34O7	1E-13	
1210. BPINAO2 + C10H17O3O2 → C20H34O6	1E-13	
1211. BPINBO2 + C10H17O3O2 → C20H34O6	1E-13	
1212. BPINCO2 + C10H17O3O2 → C20H34O6	1E-13	
1213. C918CO3 + C10H17O3O2 → C20H34O7	1E-13	
1214. NAPINAO2 + C10H17O3O2 → C20H35O5NO3	1E-13	HOM dimers containing NO_3
1215. NAPINBO2 + C10H17O3O2 → C20H35O5NO3	1E-13	HOM dimers containing NO_3
1216. NC102O2 + C10H17O3O2 → C20H35O6NO3	1E-13	HOM dimers containing NO_3
1217. NC101O2 + C10H17O3O2 → C20H35O6NO3	1E-13	HOM dimers containing NO_3
1218. NLIMO2 + C10H17O3O2 → C20H35O5NO3	1E-13	HOM dimers containing NO_3
1219. NLIMALO2 + C10H17O3O2 → C20H35O6NO3	1E-13	HOM dimers containing NO_3
1220. NC91CO3 + C10H17O3O2 → C20H35O6NO3	1E-13	HOM dimers containing NO_3
1221. NBPINAO2 + C10H17O3O2 → C20H35O5NO3	1E-13	HOM dimers containing NO_3
1222. NBPINBO2 + C10H17O3O2 → C20H35O5NO3	1E-13	HOM dimers containing NO_3
1223. C96O2 + C10H17O3O2 → C19H32O6	1E-13	
1224. C89CO3 + C10H17O3O2 → C19H32O7	1E-13	
1225. C920O2 + C10H17O3O2 → C19H32O7	1E-13	
1226. C97O2 + C10H17O3O2 → C19H32O7	1E-13	
1227. C85CO3 + C10H17O3O2 → C19H32O7	1E-13	
1228. C811CO3 + C10H17O3O2 → C19H32O8	1E-13	
1229. C921O2 + C10H17O3O2 → C19H32O8	1E-13	
1230. C98O2 + C10H17O3O2 → C19H32O8	1E-13	
1231. C922O2+ C10H17O3O2 → C19H32O9	1E-13	
1232. C923O2 + C10H17O3O2 → C19H32O6	1E-13	
1233. C924O2 + C10H17O3O2 → C19H32O7	1E-13	
1234. C816CO3 + C10H17O3O2 → C19H32O7	1E-13	
1235. NORLIMO2 + C10H17O3O2 → C19H32O8	1E-13	
1236. LMKAO2 + C10H17O3O2 → C19H32O7	1E-13	
1237. LMKBO2 + C10H17O3O2 → C19H32O7	1E-13	
1238. C926O2 + C10H17O3O2 → C19H32O8	1E-13	
1239. C817CO3 + C10H17O3O2 → C19H32O8	1E-13	
1240. LMLKAO2 + C10H17O3O2 → C19H32O8	1E-13	
1241. LMLKBO2 + C10H17O3O2 → C19H32O8	1E-13	
1242. C823CO3 + C10H17O3O2 → C19H32O8	1E-13	
1243. C925O2 + C10H17O3O2 → C19H32O9	1E-13	
1244. NOPINAO2+ C10H17O3O2 → C19H32O6	1E-13	
1245. NOPINBO2+ C10H17O3O2 → C19H32O6	1E-13	
1246. NOPINCO2+ C10H17O3O2 → C19H32O6	1E-13	
1247. NOPINDO2+ C10H17O3O2 → C19H32O6	1E-13	
1248. C918O2+ C10H17O3O2 → C19H32O7	1E-13	
1249. C9DCO2+ C10H17O3O2 → C19H32O7	1E-13	
1250. C915O2+ C10H17O3O2 → C19H32O7	1E-13	
1251. C917O2+ C10H17O3O2 → C19H32O7	1E-13	
1252. C919O2+ C10H17O3O2 → C19H32O8	1E-13	
1253. C914O2+ C10H17O3O2 → C19H32O8	1E-13	
1254. C916O2+ C10H17O3O2 → C19H32O8	1E-13	
1255. C88CO3+ C10H17O3O2 → C19H32O8	1E-13	
1256. C87CO3+ C10H17O3O2 → C19H32O9	1E-13	
1257. C822CO3+ C10H17O3O2 → C19H32O7	1E-13	
1258. NLMKAO2+ C10H17O3O2 → C19H33O6NO3	1E-13	
1259. C85O2 + C10H17O3O2 → C18H30O6	1E-13	
1260. C89O2 + C10H17O3O2 → C18H30O6	1E-13	
1261. C86O2 + C10H17O3O2 → C18H30O7	1E-13	
1262. C811O2 + C10H17O3O2 → C18H30O7	1E-13	
1263. C810O2 + C10H17O3O2 → C18H30O7	1E-13	
1264. C812O2 + C10H17O3O2 → C18H30O8	1E-13	
1265. C813O2 + C10H17O3O2 → C18H30O6	1E-13	
1266. C729CO3 + C10H17O3O2 → C18H30O7	1E-13	
1267. C816O2 + C10H17O3O2 → C18H30O6	1E-13	
1268. C817O2 + C10H17O3O2 → C18H30O7	1E-13	
1269. C826O2 + C10H17O3O2 → C18H30O8	1E-13	
1270. C822O2 + C10H17O3O2 → C18H30O6	1E-13	

1271. C818O2 + C10H17O3O2 → C18H30O8	1E-13	
1272. C823O2 + C10H17O3O2 → C18H30O7	1E-13	
1273. C819O2 + C10H17O3O2 → C18H30O8	1E-13	
1274. C727CO3 + C10H17O3O2 → C18H30O8	1E-13	
1275. C731CO3 + C10H17O3O2 → C18H30O8	1E-13	
1276. C824O2 + C10H17O3O2 → C18H30O7	1E-13	
1277. C820O2 + C10H17O3O2 → C18H30O9	1E-13	
1278. C825O2 + C10H17O3O2 → C18H30O8	1E-13	
1279. C821O2 + C10H17O3O2 → C18H30O8	1E-13	
1280. C732CO3 + C10H17O3O2 → C18H30O9	1E-13	
1281. C8BCO2 + C10H17O3O2 → C18H30O5	1E-13	
1282. C88O2 + C10H17O3O2 → C18H30O7	1E-13	
1283. C718CO3 + C10H17O3O2 → C18H30O8	1E-13	
1284. C87O2 + C10H17O3O2 → C18H30O8	1E-13	
1285. C721CO3 + C10H17O3O2 → C18H30O8	1E-13	
1286. NC826O2 + C10H17O3O2 → C18H31O7NO3	1E-13	
1287. APINAO2 + C10H17O4O2 → C20H34O7	1E-13	
1288. APINBO2 + C10H17O4O2 → C20H34O7	1E-13	
1289. APINCO2 + C10H17O4O2 → C20H34O7	1E-13	
1290. C107O2 + C10H17O4O2 → C20H34O8	1E-13	
1291. C109O2 + C10H17O4O2 → C20H34O8	1E-13	
1292. C106O2 + C10H17O4O2 → C20H34O9	1E-13	
1293. C920CO3 + C10H17O4O2 → C20H34O9	1E-13	
1294. C108O2 + C10H17O4O2 → C20H34O9	1E-13	
1295. PINALO2 + C10H17O4O2 → C20H34O8	1E-13	
1296. C96CO3 + C10H17O4O2 → C20H34O8	1E-13	
1297. C923CO3 + C10H17O4O2 → C20H34O8	1E-13	
1298. LIMAO2 + C10H17O4O2 → C20H34O7	1E-13	
1299. LIMALBO2 + C10H17O4O2 → C20H34O8	1E-13	
1300. LIMCO2 + C10H17O4O2 → C20H34O7	1E-13	
1301. LIMALO2 + C10H17O4O2 → C20H34O8	1E-13	
1302. LIMBO2 + C10H17O4O2 → C20H34O7	1E-13	
1303. LIMALAO2 + C10H17O4O2 → C20H34O8	1E-13	
1304. BPINAO2 + C10H17O4O2 → C20H34O7	1E-13	
1305. BPINBO2 + C10H17O4O2 → C20H34O7	1E-13	
1306. BPINCO2 + C10H17O4O2 → C20H34O7	1E-13	
1307. C918CO3 + C10H17O4O2 → C20H34O8	1E-13	
1308. NAPINAO2 + C10H17O4O2 → C20H35O6NO3	1E-13	HOM dimers containing NO_3
1309. NAPINBO2 + C10H17O4O2 → C20H35O6NO3	1E-13	HOM dimers containing NO_3
1310. NC102O2 + C10H17O4O2 → C20H35O7NO3	1E-13	HOM dimers containing NO_3
1311. NC101O2 + C10H17O4O2 → C20H35O7NO3	1E-13	HOM dimers containing NO_3
1312. NLIMO2 + C10H17O4O2 → C20H35O6NO3	1E-13	HOM dimers containing NO_3
1313. NLIMALO2 + C10H17O4O2 → C20H35O7NO3	1E-13	HOM dimers containing NO_3
1314. NC91CO3 + C10H17O4O2 → C20H35O7NO3	1E-13	HOM dimers containing NO_3
1315. NBPINAO2 + C10H17O4O2 → C20H35O6NO3	1E-13	HOM dimers containing NO_3
1316. NBPINBO2 + C10H17O4O2 → C20H35O6NO3	1E-13	HOM dimers containing NO_3
1317. C96O2 + C10H17O4O2 → C19H32O7	1E-13	
1318. C89CO3 + C10H17O4O2 → C19H32O8	1E-13	
1319. C920O2 + C10H17O4O2 → C19H32O8	1E-13	
1320. C97O2 + C10H17O4O2 → C19H32O8	1E-13	
1321. C85CO3 + C10H17O4O2 → C19H32O8	1E-13	
1322. C811CO3 + C10H17O4O2 → C19H32O9	1E-13	
1323. C921O2 + C10H17O4O2 → C19H32O9	1E-13	
1324. C98O2 + C10H17O4O2 → C19H32O9	1E-13	
1325. C922O2+ C10H17O4O2 → C19H32O10	1E-13	
1326. C923O2 + C10H17O4O2 → C19H32O7	1E-13	
1327. C924O2 + C10H17O4O2 → C19H32O8	1E-13	
1328. C816CO3 + C10H17O4O2 → C19H32O8	1E-13	
1329. NORLIMO2 + C10H17O4O2 → C19H32O9	1E-13	
1330. LMKAO2 + C10H17O4O2 → C19H32O8	1E-13	
1331. LMKBO2 + C10H17O4O2 → C19H32O8	1E-13	
1332. C926O2 + C10H17O4O2 → C19H32O9	1E-13	
1333. C817CO3 + C10H17O4O2 → C19H32O9	1E-13	
1334. LMLKAO2 + C10H17O4O2 → C19H32O9	1E-13	
1335. LMLKBO2 + C10H17O4O2 → C19H32O9	1E-13	
1336. C823CO3 + C10H17O4O2 → C19H32O9	1E-13	
1337. C925O2 + C10H17O4O2 → C19H32O10	1E-13	
1338. NOPINAO2+ C10H17O4O2 → C19H32O7	1E-13	
1339. NOPINBO2+ C10H17O4O2 → C19H32O7	1E-13	
1340. NOPINCO2+ C10H17O4O2 → C19H32O7	1E-13	
1341. NOPINDO2+ C10H17O4O2 → C19H32O7	1E-13	
1342. C918O2+ C10H17O4O2 → C19H32O8	1E-13	

1343. C9DCO2+ C10H17O4O2 → C19H32O8	1E-13	
1344. C915O2+ C10H17O4O2 → C19H32O8	1E-13	
1345. C917O2+ C10H17O4O2 → C19H32O8	1E-13	
1346. C919O2+ C10H17O4O2 → C19H32O9	1E-13	
1347. C914O2+ C10H17O4O2 → C19H32O9	1E-13	
1348. C916O2+ C10H17O4O2 → C19H32O9	1E-13	
1349. C88CO3+ C10H17O4O2 → C19H32O9	1E-13	
1350. C87CO3+ C10H17O4O2 → C19H32O10	1E-13	
1351. C822CO3+ C10H17O4O2 → C19H32O8	1E-13	
1352. NLMKAO2+ C10H17O4O2 → C19H33O7NO3	1E-13	
1353. C85O2 + C10H17O4O2 → C18H30O7	1E-13	
1354. C89O2 + C10H17O4O2 → C18H30O7	1E-13	
1355. C86O2 + C10H17O4O2 → C18H30O8	1E-13	
1356. C811O2 + C10H17O4O2 → C18H30O8	1E-13	
1357. C810O2 + C10H17O4O2 → C18H30O8	1E-13	
1358. C812O2 + C10H17O4O2 → C18H30O9	1E-13	
1359. C813O2 + C10H17O4O2 → C18H30O7	1E-13	
1360. C729CO3 + C10H17O4O2 → C18H30O8	1E-13	
1361. C816O2 + C10H17O4O2 → C18H30O7	1E-13	
1362. C817O2 + C10H17O4O2 → C18H30O8	1E-13	
1363. C826O2 + C10H17O4O2 → C18H30O9	1E-13	
1364. C822O2 + C10H17O4O2 → C18H30O7	1E-13	
1365. C818O2 + C10H17O4O2 → C18H30O9	1E-13	
1366. C823O2 + C10H17O4O2 → C18H30O8	1E-13	
1367. C819O2 + C10H17O4O2 → C18H30O9	1E-13	
1368. C727CO3 + C10H17O4O2 → C18H30O9	1E-13	
1369. C731CO3 + C10H17O4O2 → C18H30O9	1E-13	
1370. C824O2 + C10H17O4O2 → C18H30O8	1E-13	
1371. C820O2 + C10H17O4O2 → C18H30O10	1E-13	
1372. C825O2 + C10H17O4O2 → C18H30O9	1E-13	
1373. C821O2 + C10H17O4O2 → C18H30O9	1E-13	
1374. C732CO3 + C10H17O4O2 → C18H30O10	1E-13	
1375. C8BCO2 + C10H17O4O2 → C18H30O6	1E-13	
1376. C88O2 + C10H17O4O2 → C18H30O8	1E-13	
1377. C718CO3 + C10H17O4O2 → C18H30O9	1E-13	
1378. C87O2 + C10H17O4O2 → C18H30O9	1E-13	
1379. C721CO3 + C10H17O4O2 → C18H30O9	1E-13	
1380. NC826O2 + C10H17O4O2 → C18H31O8NO3	1E-13	
1381. APINAO2 + C10H17O5O2 → C20H34O8	5E-13	
1382. APINBO2 + C10H17O5O2 → C20H34O8	5E-13	
1383. APINCO2 + C10H17O5O2 → C20H34O8	5E-13	
1384. C107O2 + C10H17O5O2 → C20H34O9	5E-13	
1385. C109O2 + C10H17O5O2 → C20H34O9	5E-13	
1386. C106O2 + C10H17O5O2 → C20H34O10	5E-13	
1387. C920CO3 + C10H17O5O2 → C20H34O10	5E-13	
1388. C108O2 + C10H17O5O2 → C20H34O10	5E-13	
1389. PINALO2 + C10H17O5O2 → C20H34O9	5E-13	
1390. C96CO3 + C10H17O5O2 → C20H34O9	5E-13	
1391. C923CO3 + C10H17O5O2 → C20H34O9	5E-13	
1392. LIMAO2 + C10H17O5O2 → C20H34O8	5E-13	
1393. LIMALBO2 + C10H17O5O2 → C20H34O9	5E-13	
1394. LIMCO2 + C10H17O5O2 → C20H34O8	5E-13	
1395. LIMALO2 + C10H17O5O2 → C20H34O9	5E-13	
1396. LIMBO2 + C10H17O5O2 → C20H34O8	5E-13	
1397. LIMALAO2 + C10H17O5O2 → C20H34O9	5E-13	
1398. BPINAO2 + C10H17O5O2 → C20H34O8	5E-13	
1399. BPINBO2 + C10H17O5O2 → C20H34O8	5E-13	
1400. BPINCO2 + C10H17O5O2 → C20H34O8	5E-13	
1401. C918CO3 + C10H17O5O2 → C20H34O9	5E-13	
1402. NAPINAO2 + C10H17O5O2 → C20H35O7NO3	5E-13	HOM dimers containing NO_3
1403. NAPINBO2 + C10H17O5O2 → C20H35O7NO3	5E-13	HOM dimers containing NO_3
1404. NC102O2 + C10H17O5O2 → C20H35O8NO3	5E-13	HOM dimers containing NO_3
1405. NC101O2 + C10H17O5O2 → C20H35O8NO3	5E-13	HOM dimers containing NO_3
1406. NLIMO2 + C10H17O5O2 → C20H35O7NO3	5E-13	HOM dimers containing NO_3
1407. NLIMALO2 + C10H17O5O2 → C20H35O8NO3	5E-13	HOM dimers containing NO_3
1408. NC91CO3 + C10H17O5O2 → C20H35O8NO3	5E-13	HOM dimers containing NO_3
1409. NBPINAO2 + C10H17O5O2 → C20H35O7NO3	5E-13	HOM dimers containing NO_3
1410. NBPINBO2 + C10H17O5O2 → C20H35O7NO3	5E-13	HOM dimers containing NO_3
1411. C96O2 + C10H17O5O2 → C19H32O8	5E-13	
1412. C89CO3 + C10H17O5O2 → C19H32O9	5E-13	
1413. C920O2 + C10H17O5O2 → C19H32O9	5E-13	

1414. C97O2 + C10H17O5O2 → C19H32O9	5E-13
1415. C85CO3 + C10H17O5O2 → C19H32O9	5E-13
1416. C811CO3 + C10H17O5O2 → C19H32O10	5E-13
1417. C921O2 + C10H17O5O2 → C19H32O10	5E-13
1418. C98O2 + C10H17O5O2 → C19H32O10	5E-13
1419. C922O2+ C10H17O5O2 → C19H32O11	5E-13
1420. C923O2 + C10H17O5O2 → C19H32O8	5E-13
1421. C924O2 + C10H17O5O2 → C19H32O9	5E-13
1422. C816CO3 + C10H17O5O2 → C19H32O9	5E-13
1423. NORLIMO2 + C10H17O5O2 → C19H32O10	5E-13
1424. LMKAO2 + C10H17O5O2 → C19H32O9	5E-13
1425. LMKBO2 + C10H17O5O2 → C19H32O9	5E-13
1426. C926O2 + C10H17O5O2 → C19H32O10	5E-13
1427. C817CO3 + C10H17O5O2 → C19H32O10	5E-13
1428. LMLKAO2 + C10H17O5O2 → C19H32O10	5E-13
1429. LMLKBO2 + C10H17O5O2 → C19H32O10	5E-13
1430. C823CO3 + C10H17O5O2 → C19H32O10	5E-13
1431. C925O2 + C10H17O5O2 → C19H32O11	5E-13
1432. NOPINAO2+ C10H17O5O2 → C19H32O8	5E-13
1433. NOPINBO2+ C10H17O5O2 → C19H32O8	5E-13
1434. NOPINCO2+ C10H17O5O2 → C19H32O8	5E-13
1435. NOPINDO2+ C10H17O5O2 → C19H32O8	5E-13
1436. C918O2+ C10H17O5O2 → C19H32O9	5E-13
1437. C9DCO2+ C10H17O5O2 → C19H32O9	5E-13
1438. C915O2+ C10H17O5O2 → C19H32O9	5E-13
1439. C917O2+ C10H17O5O2 → C19H32O9	5E-13
1440. C919O2+ C10H17O5O2 → C19H32O10	5E-13
1441. C914O2+ C10H17O5O2 → C19H32O10	5E-13
1442. C916O2+ C10H17O5O2 → C19H32O10	5E-13
1443. C88CO3+ C10H17O5O2 → C19H32O10	5E-13
1444. C87CO3+ C10H17O5O2 → C19H32O11	5E-13
1445. C822CO3+ C10H17O5O2 → C19H32O9	5E-13
1446. NLMKAO2+ C10H17O5O2 → C19H33O8NO3	5E-13

1447. C85O2 + C10H17O5O2 → C18H30O8	5E-13
1448. C89O2 + C10H17O5O2 → C18H30O8	5E-13
1449. C86O2 + C10H17O5O2 → C18H30O9	5E-13
1450. C811O2 + C10H17O5O2 → C18H30O9	5E-13
1451. C810O2 + C10H17O5O2 → C18H30O9	5E-13
1452. C812O2 + C10H17O5O2 → C18H30O10	5E-13
1453. C813O2 + C10H17O5O2 → C18H30O8	5E-13
1454. C729CO3 + C10H17O5O2 → C18H30O9	5E-13
1455. C816O2 + C10H17O5O2 → C18H30O8	5E-13
1456. C817O2 + C10H17O5O2 → C18H30O9	5E-13
1457. C826O2 + C10H17O5O2 → C18H30O10	5E-13
1458. C822O2 + C10H17O5O2 → C18H30O8	5E-13
1459. C818O2 + C10H17O5O2 → C18H30O10	5E-13
1460. C823O2 + C10H17O5O2 → C18H30O9	5E-13
1461. C819O2 + C10H17O5O2 → C18H30O10	5E-13
1462. C727CO3 + C10H17O5O2 → C18H30O10	5E-13
1463. C731CO3 + C10H17O5O2 → C18H30O10	5E-13
1464. C824O2 + C10H17O5O2 → C18H30O9	5E-13
1465. C820O2 + C10H17O5O2 → C18H30O11	5E-13
1466. C825O2 + C10H17O5O2 → C18H30O10	5E-13
1467. C821O2 + C10H17O5O2 → C18H30O10	5E-13
1468. C732CO3 + C10H17O5O2 → C18H30O11	5E-13
1469. C8BCO2 + C10H17O5O2 → C18H30O7	5E-13
1470. C88O2 + C10H17O5O2 → C18H30O9	5E-13
1471. C718CO3 + C10H17O5O2 → C18H30O10	5E-13
1472. C87O2 + C10H17O5O2 → C18H30O10	5E-13
1473. C721CO3 + C10H17O5O2 → C18H30O10	5E-13
1474. NC826O2 + C10H17O5O2 → C18H31O9NO3	5E-13

1475. APINAO2 + C10H17O6O2 → C20H34O9	2E-12
1476. APINBO2 + C10H17O6O2 → C20H34O9	2E-12
1477. APINCO2 + C10H17O6O2 → C20H34O9	2E-12
1478. C107O2 + C10H17O6O2 → C20H34O10	2E-12
1479. C109O2 + C10H17O6O2 → C20H34O10	2E-12
1480. C106O2 + C10H17O6O2 → C20H34O11	2E-12
1481. C920CO3 + C10H17O6O2 → C20H34O11	2E-12
1482. C108O2 + C10H17O6O2 → C20H34O11	2E-12
1483. PINALO2 + C10H17O6O2 → C20H34O10	2E-12
1484. C96CO3 + C10H17O6O2 → C20H34O10	2E-12
1485. C923CO3 + C10H17O6O2 → C20H34O10	2E-12

1486. LIMAO2 + C10H17O6O2 → C20H34O9	2E-12	
1487. LIMALBO2 + C10H17O6O2 → C20H34O10	2E-12	
1488. LIMCO2 + C10H17O6O2 → C20H34O9	2E-12	
1489. LIMALO2 + C10H17O6O2 → C20H34O10	2E-12	
1490. LIMBO2 + C10H17O6O2 → C20H34O9	2E-12	
1491. LIMALAO2 + C10H17O6O2 → C20H34O10	2E-12	
1492. BPINAO2 + C10H17O6O2 → C20H34O9	2E-12	
1493. BPINBO2 + C10H17O6O2 → C20H34O9	2E-12	
1494. BPINCO2 + C10H17O6O2 → C20H34O9	2E-12	
1495. C918CO3 + C10H17O6O2 → C20H34O10	2E-12	
1496. NAPINAO2 + C10H17O6O2 → C20H35O8NO3	2E-12	HOM dimers containing NO_3
1497. NAPINBO2 + C10H17O6O2 → C20H35O8NO3	2E-12	HOM dimers containing NO_3
1498. NC102O2 + C10H17O6O2 → C20H35O9NO3	2E-12	HOM dimers containing NO_3
1499. NC101O2 + C10H17O6O2 → C20H35O9NO3	2E-12	HOM dimers containing NO_3
1500. NLIMO2 + C10H17O6O2 → C20H35O8NO3	2E-12	HOM dimers containing NO_3
1501. NLIMALO2 + C10H17O6O2 → C20H35O9NO3	2E-12	HOM dimers containing NO_3
1502. NC91CO3 + C10H17O6O2 → C20H35O9NO3	2E-12	HOM dimers containing NO_3
1503. NBPINAO2 + C10H17O6O2 → C20H35O8NO3	2E-12	HOM dimers containing NO_3
1504. NBPINBO2 + C10H17O6O2 → C20H35O8NO3	2E-12	HOM dimers containing NO_3

1505. C96O2 + C10H17O6O2 → C19H32O9	2E-12
1506. C89CO3 + C10H17O6O2 → C19H32O10	2E-12
1507. C920O2 + C10H17O6O2 → C19H32O10	2E-12
1508. C97O2 + C10H17O6O2 → C19H32O10	2E-12
1509. C85CO3 + C10H17O6O2 → C19H32O10	2E-12
1510. C811CO3 + C10H17O6O2 → C19H32O11	2E-12
1511. C921O2 + C10H17O6O2 → C19H32O11	2E-12
1512. C98O2 + C10H17O6O2 → C19H32O11	2E-12
1513. C922O2+ C10H17O6O2 → C19H32O12	2E-12
1514. C923O2 + C10H17O6O2 → C19H32O9	2E-12
1515. C924O2 + C10H17O6O2 → C19H32O10	2E-12
1516. C816CO3 + C10H17O6O2 → C19H32O10	2E-12
1517. NORLIMO2 + C10H17O6O2 → C19H32O11	2E-12
1518. LMKAO2 + C10H17O6O2 → C19H32O10	2E-12
1519. LMKBO2 + C10H17O6O2 → C19H32O10	2E-12
1520. C926O2 + C10H17O6O2 → C19H32O11	2E-12
1521. C817CO3 + C10H17O6O2 → C19H32O11	2E-12
1522. LMLKAO2 + C10H17O6O2 → C19H32O11	2E-12
1523. LMLKBO2 + C10H17O6O2 → C19H32O11	2E-12
1524. C823CO3 + C10H17O6O2 → C19H32O11	2E-12
1525. C925O2 + C10H17O6O2 → C19H32O12	2E-12
1526. NOPINAO2+ C10H17O6O2 → C19H32O9	2E-12
1527. NOPINBO2+ C10H17O6O2 → C19H32O9	2E-12
1528. NOPINCO2+ C10H17O6O2 → C19H32O9	2E-12
1529. NOPINDO2+ C10H17O6O2 → C19H32O9	2E-12
1530. C918O2+ C10H17O6O2 → C19H32O10	2E-12
1531. C9DCO2+ C10H17O6O2 → C19H32O10	2E-12
1532. C915O2+ C10H17O6O2 → C19H32O10	2E-12
1533. C917O2+ C10H17O6O2 → C19H32O10	2E-12
1534. C919O2+ C10H17O6O2 → C19H32O11	2E-12
1535. C914O2+ C10H17O6O2 → C19H32O11	2E-12
1536. C916O2+ C10H17O6O2 → C19H32O11	2E-12
1537. C88CO3+ C10H17O6O2 → C19H32O11	2E-12
1538. C87CO3+ C10H17O6O2 → C19H32O12	2E-12
1539. C822CO3+ C10H17O6O2 → C19H32O10	2E-12
1540. NLMKAO2+ C10H17O6O2 → C19H33O9NO3	2E-12

1541. C85O2 + C10H17O6O2 → C18H30O9	2E-12
1542. C89O2 + C10H17O6O2 → C18H30O9	2E-12
1543. C86O2 + C10H17O6O2 → C18H30O10	2E-12
1544. C811O2 + C10H17O6O2 → C18H30O10	2E-12
1545. C810O2 + C10H17O6O2 → C18H30O10	2E-12
1546. C812O2 + C10H17O6O2 → C18H30O11	2E-12
1547. C813O2 + C10H17O6O2 → C18H30O9	2E-12
1548. C729CO3 + C10H17O6O2 → C18H30O10	2E-12
1549. C816O2 + C10H17O6O2 → C18H30O9	2E-12
1550. C817O2 + C10H17O6O2 → C18H30O10	2E-12
1551. C826O2 + C10H17O6O2 → C18H30O11	2E-12
1552. C822O2 + C10H17O6O2 → C18H30O9	2E-12
1553. C818O2 + C10H17O6O2 → C18H30O11	2E-12
1554. C823O2 + C10H17O6O2 → C18H30O10	2E-12
1555. C819O2 + C10H17O6O2 → C18H30O11	2E-12
1556. C727CO3 + C10H17O6O2 → C18H30O11	2E-12
1557. C731CO3 + C10H17O6O2 → C18H30O11	2E-12

1558. C824O2 + C10H17O6O2 → C18H30O10	2E-12	
1559. C820O2 + C10H17O6O2 → C18H30O12	2E-12	
1560. C825O2 + C10H17O6O2 → C18H30O11	2E-12	
1561. C821O2 + C10H17O6O2 → C18H30O11	2E-12	
1562. C732CO3 + C10H17O6O2 → C18H30O12	2E-12	
1563. C8BCO2 + C10H17O6O2 → C18H30O8	2E-12	
1564. C88O2 + C10H17O6O2 → C18H30O10	2E-12	
1565. C718CO3 + C10H17O6O2 → C18H30O11	2E-12	
1566. C87O2 + C10H17O6O2 → C18H30O11	2E-12	
1567. C721CO3 + C10H17O6O2 → C18H30O11	2E-12	
1568. NC826O2 + C10H17O6O2 → C18H31O10NO3	2E-12	

1569. APINAO2 + C10H17O7O2 → C20H34O10	3E-12	
1570. APINBO2 + C10H17O7O2 → C20H34O10	3E-12	
1571. APINCO2 + C10H17O7O2 → C20H34O10	3E-12	
1572. C107O2 + C10H17O7O2 → C20H34O11	3E-12	
1573. C109O2 + C10H17O7O2 → C20H34O11	3E-12	
1574. C106O2 + C10H17O7O2 → C20H34O12	3E-12	
1575. C920CO3 + C10H17O7O2 → C20H34O12	3E-12	
1576. C108O2 + C10H17O7O2 → C20H34O12	3E-12	
1577. PINALO2 + C10H17O7O2 → C20H34O11	3E-12	
1578. C96CO3 + C10H17O7O2 → C20H34O11	3E-12	
1579. C923CO3 + C10H17O7O2 → C20H34O11	3E-12	
1580. LIMAO2 + C10H17O7O2 → C20H34O10	3E-12	
1581. LIMALBO2 + C10H17O7O2 → C20H34O11	3E-12	
1582. LIMCO2 + C10H17O7O2 → C20H34O10	3E-12	
1583. LIMALO2 + C10H17O7O2 → C20H34O11	3E-12	
1584. LIMBO2 + C10H17O7O2 → C20H34O10	3E-12	
1585. LIMALAO2 + C10H17O7O2 → C20H34O11	3E-12	
1586. BPINAO2 + C10H17O7O2 → C20H34O10	3E-12	
1587. BPINBO2 + C10H17O7O2 → C20H34O10	3E-12	
1588. BPINCO2 + C10H17O7O2 → C20H34O10	3E-12	
1589. C918CO3 + C10H17O7O2 → C20H34O11	3E-12	
1590. NAPINAO2 + C10H17O7O2 → C20H35O9NO3	3E-12	HOM dimers containing NO_3
1591. NAPINBO2 + C10H17O7O2 → C20H35O9NO3	3E-12	HOM dimers containing NO_3
1592. NC102O2 + C10H17O7O2 → C20H35O10NO3	3E-12	HOM dimers containing NO_3
1593. NC101O2 + C10H17O7O2 → C20H35O10NO3	3E-12	HOM dimers containing NO_3
1594. NLIMO2 + C10H17O7O2 → C20H35O9NO3	3E-12	HOM dimers containing NO_3
1595. NLIMALO2 + C10H17O7O2 → C20H35O10NO3	3E-12	HOM dimers containing NO_3
1596. NC91CO3 + C10H17O7O2 → C20H35O10NO3	3E-12	HOM dimers containing NO_3
1597. NBPINAO2 + C10H17O7O2 → C20H35O9NO3	3E-12	HOM dimers containing NO_3
1598. NBPINBO2 + C10H17O7O2 → C20H35O9NO3	3E-12	HOM dimers containing NO_3

1599. C96O2 + C10H17O7O2 → C19H32O10	3E-12	
1600. C89CO3 + C10H17O7O2 → C19H32O11	3E-12	
1601. C920O2 + C10H17O7O2 → C19H32O11	3E-12	
1602. C97O2 + C10H17O7O2 → C19H32O11	3E-12	
1603. C85CO3 + C10H17O7O2 → C19H32O11	3E-12	
1604. C811CO3 + C10H17O7O2 → C19H32O12	3E-12	
1605. C921O2 + C10H17O7O2 → C19H32O12	3E-12	
1606. C98O2 + C10H17O7O2 → C19H32O12	3E-12	
1607. C922O2+ C10H17O7O2 → C19H32O13	3E-12	
1608. C923O2 + C10H17O7O2 → C19H32O10	3E-12	
1609. C924O2 + C10H17O7O2 → C19H32O11	3E-12	
1610. C816CO3 + C10H17O7O2 → C19H32O11	3E-12	
1611. NORLIMO2 + C10H17O7O2 → C19H32O12	3E-12	
1612. LMKAO2 + C10H17O7O2 → C19H32O11	3E-12	
1613. LMKBO2 + C10H17O7O2 → C19H32O11	3E-12	
1614. C926O2 + C10H17O7O2 → C19H32O12	3E-12	
1615. C817CO3 + C10H17O7O2 → C19H32O12	3E-12	
1616. LMLKAO2 + C10H17O7O2 → C19H32O12	3E-12	
1617. LMLKBO2 + C10H17O7O2 → C19H32O12	3E-12	
1618. C823CO3 + C10H17O7O2 → C19H32O12	3E-12	
1619. C925O2 + C10H17O7O2 → C19H32O13	3E-12	
1620. NOPINAO2+ C10H17O7O2 → C19H32O10	3E-12	
1621. NOPINBO2+ C10H17O7O2 → C19H32O10	3E-12	
1622. NOPINCO2+ C10H17O7O2 → C19H32O10	3E-12	
1623. NOPINDO2+ C10H17O7O2 → C19H32O10	3E-12	
1624. C918O2+ C10H17O7O2 → C19H32O11	3E-12	
1625. C9DCO2+ C10H17O7O2 → C19H32O11	3E-12	
1626. C915O2+ C10H17O7O2 → C19H32O11	3E-12	
1627. C917O2+ C10H17O7O2 → C19H32O11	3E-12	
1628. C919O2+ C10H17O7O2 → C19H32O12	3E-12	
1629. C914O2+ C10H17O7O2 → C19H32O12	3E-12	

1630. C916O2+ C10H17O7O2 → C19H32O12	3E-12	
1631. C88CO3+ C10H17O7O2 → C19H32O12	3E-12	
1632. C87CO3+ C10H17O7O2 → C19H32O13	3E-12	
1633. C822CO3+ C10H17O7O2 → C19H32O11	3E-12	
1634. NLMKAO2+ C10H17O7O2 → C19H33O10NO3	3E-12	
1635. C85O2 + C10H17O7O2 → C18H30O10	3E-12	
1636. C89O2 + C10H17O7O2 → C18H30O10	3E-12	
1637. C86O2 + C10H17O7O2 → C18H30O11	3E-12	
1638. C811O2 + C10H17O7O2 → C18H30O11	3E-12	
1639. C810O2 + C10H17O7O2 → C18H30O11	3E-12	
1640. C812O2 + C10H17O7O2 → C18H30O12	3E-12	
1641. C813O2 + C10H17O7O2 → C18H30O10	3E-12	
1642. C729CO3 + C10H17O7O2 → C18H30O11	3E-12	
1643. C816O2 + C10H17O7O2 → C18H30O10	3E-12	
1644. C817O2 + C10H17O7O2 → C18H30O11	3E-12	
1645. C826O2 + C10H17O7O2 → C18H30O12	3E-12	
1646. C822O2 + C10H17O7O2 → C18H30O10	3E-12	
1647. C818O2 + C10H17O7O2 → C18H30O12	3E-12	
1648. C823O2 + C10H17O7O2 → C18H30O11	3E-12	
1649. C819O2 + C10H17O7O2 → C18H30O12	3E-12	
1650. C727CO3 + C10H17O7O2 → C18H30O12	3E-12	
1651. C731CO3 + C10H17O7O2 → C18H30O12	3E-12	
1652. C824O2 + C10H17O7O2 → C18H30O11	3E-12	
1653. C820O2 + C10H17O7O2 → C18H30O13	3E-12	
1654. C825O2 + C10H17O7O2 → C18H30O12	3E-12	
1655. C821O2 + C10H17O7O2 → C18H30O12	3E-12	
1656. C732CO3 + C10H17O7O2 → C18H30O13	3E-12	
1657. C8BCO2 + C10H17O7O2 → C18H30O9	3E-12	
1658. C88O2 + C10H17O7O2 → C18H30O11	3E-12	
1659. C718CO3 + C10H17O7O2 → C18H30O12	3E-12	
1660. C87O2 + C10H17O7O2 → C18H30O12	3E-12	
1661. C721CO3 + C10H17O7O2 → C18H30O12	3E-12	
1662. NC826O2 + C10H17O7O2 → C18H31O11NO3	3E-12	

1663. APINAO2 + C10H17O8O2 → C20H34O11	3E-12	
1664. APINBO2 + C10H17O8O2 → C20H34O11	3E-12	
1665. APINCO2 + C10H17O8O2 → C20H34O11	3E-12	
1666. C107O2 + C10H17O8O2 → C20H34O12	3E-12	
1667. C109O2 + C10H17O8O2 → C20H34O12	3E-12	
1668. C106O2 + C10H17O8O2 → C20H34O13	3E-12	
1669. C920CO3 + C10H17O8O2 → C20H34O13	3E-12	
1670. C108O2 + C10H17O8O2 → C20H34O13	3E-12	
1671. PINALO2 + C10H17O8O2 → C20H34O12	3E-12	
1672. C96CO3 + C10H17O8O2 → C20H34O12	3E-12	
1673. C923CO3 + C10H17O8O2 → C20H34O12	3E-12	
1674. LIMAO2 + C10H17O8O2 → C20H34O11	3E-12	
1675. LIMALBO2 + C10H17O8O2 → C20H34O12	3E-12	
1676. LIMCO2 + C10H17O8O2 → C20H34O11	3E-12	
1677. LIMALO2 + C10H17O8O2 → C20H34O12	3E-12	
1678. LIMBO2 + C10H17O8O2 → C20H34O11	3E-12	
1679. LIMALAO2 + C10H17O8O2 → C20H34O12	3E-12	
1680. BPINAO2 + C10H17O8O2 → C20H34O11	3E-12	
1681. BPINBO2 + C10H17O8O2 → C20H34O11	3E-12	
1682. BPINCO2 + C10H17O8O2 → C20H34O11	3E-12	
1683. C918CO3 + C10H17O8O2 → C20H34O12	3E-12	
1684. NAPINAO2 + C10H17O8O2 → C20H35O10NO3	3E-12	HOM dimers containing NO_3
1685. NAPINBO2 + C10H17O8O2 → C20H35O10NO3	3E-12	HOM dimers containing NO_3
1686. NC102O2 + C10H17O8O2 → C20H35O11NO3	3E-12	HOM dimers containing NO_3
1687. NC101O2 + C10H17O8O2 → C20H35O11NO3	3E-12	HOM dimers containing NO_3
1688. NLIMO2 + C10H17O8O2 → C20H35O10NO3	3E-12	HOM dimers containing NO_3
1689. NLIMALO2 + C10H17O8O2 → C20H35O11NO3	3E-12	HOM dimers containing NO_3
1690. NC91CO3 + C10H17O8O2 → C20H35O11NO3	3E-12	HOM dimers containing NO_3
1691. NBPINAO2 + C10H17O8O2 → C20H35O10NO3	3E-12	HOM dimers containing NO_3
1692. NBPINBO2 + C10H17O8O2 → C20H35O10NO3	3E-12	HOM dimers containing NO_3

1693. C96O2 + C10H17O8O2 → C19H32O11	3E-12	
1694. C89CO3 + C10H17O8O2 → C19H32O12	3E-12	
1695. C920O2 + C10H17O8O2 → C19H32O12	3E-12	
1696. C97O2 + C10H17O8O2 → C19H32O12	3E-12	
1697. C85CO3 + C10H17O8O2 → C19H32O12	3E-12	
1698. C811CO3 + C10H17O8O2 → C19H32O13	3E-12	
1699. C921O2 + C10H17O8O2 → C19H32O13	3E-12	
1700. C98O2 + C10H17O8O2 → C19H32O13	3E-12	
1701. C922O2+ C10H17O8O2 → C19H32O14	3E-12	

1702. C923O2 + C10H17O8O2 → C19H32O11	3E-12
1703. C924O2 + C10H17O8O2 → C19H32O12	3E-12
1704. C816CO3 + C10H17O8O2 → C19H32O12	3E-12
1705. NORLIMO2 + C10H17O8O2 → C19H32O13	3E-12
1706. LMKAO2 + C10H17O8O2 → C19H32O12	3E-12
1707. LMKBO2 + C10H17O8O2 → C19H32O12	3E-12
1708. C926O2 + C10H17O8O2 → C19H32O13	3E-12
1709. C817CO3 + C10H17O8O2 → C19H32O13	3E-12
1710. LMLKAO2 + C10H17O8O2 → C19H32O13	3E-12
1711. LMLKBO2 + C10H17O8O2 → C19H32O13	3E-12
1712. C823CO3 + C10H17O8O2 → C19H32O13	3E-12
1713. C925O2 + C10H17O8O2 → C19H32O14	3E-12
1714. NOPINAO2+ C10H17O8O2 → C19H32O11	3E-12
1715. NOPINBO2+ C10H17O8O2 → C19H32O11	3E-12
1716. NOPINCO2+ C10H17O8O2 → C19H32O11	3E-12
1717. NOPINDO2+ C10H17O8O2 → C19H32O11	3E-12
1718. C918O2+ C10H17O8O2 → C19H32O12	3E-12
1719. C9DCO2+ C10H17O8O2 → C19H32O12	3E-12
1720. C915O2+ C10H17O8O2 → C19H32O12	3E-12
1721. C917O2+ C10H17O8O2 → C19H32O12	3E-12
1722. C919O2+ C10H17O8O2 → C19H32O13	3E-12
1723. C914O2+ C10H17O8O2 → C19H32O13	3E-12
1724. C916O2+ C10H17O8O2 → C19H32O13	3E-12
1725. C88CO3+ C10H17O8O2 → C19H32O13	3E-12
1726. C87CO3+ C10H17O8O2 → C19H32O14	3E-12
1727. C822CO3+ C10H17O8O2 → C19H32O12	3E-12
1728. NLMKAO2+ C10H17O8O2 → C19H33O11NO3	3E-12

1729. C85O2 + C10H17O8O2 → C18H30O11	3E-12
1730. C89O2 + C10H17O8O2 → C18H30O11	3E-12
1731. C86O2 + C10H17O8O2 → C18H30O12	3E-12
1732. C811O2 + C10H17O8O2 → C18H30O12	3E-12
1733. C810O2 + C10H17O8O2 → C18H30O12	3E-12
1734. C812O2 + C10H17O8O2 → C18H30O13	3E-12
1735. C813O2 + C10H17O8O2 → C18H30O11	3E-12
1736. C729CO3 + C10H17O8O2 → C18H30O12	3E-12
1737. C816O2 + C10H17O8O2 → C18H30O11	3E-12
1738. C817O2 + C10H17O8O2 → C18H30O12	3E-12
1739. C826O2 + C10H17O8O2 → C18H30O13	3E-12
1740. C822O2 + C10H17O8O2 → C18H30O11	3E-12
1741. C818O2 + C10H17O8O2 → C18H30O13	3E-12
1742. C823O2 + C10H17O8O2 → C18H30O12	3E-12
1743. C819O2 + C10H17O8O2 → C18H30O13	3E-12
1744. C727CO3 + C10H17O8O2 → C18H30O13	3E-12
1745. C731CO3 + C10H17O8O2 → C18H30O13	3E-12
1746. C824O2 + C10H17O8O2 → C18H30O12	3E-12
1747. C820O2 + C10H17O8O2 → C18H30O14	3E-12
1748. C825O2 + C10H17O8O2 → C18H30O13	3E-12
1749. C821O2 + C10H17O8O2 → C18H30O13	3E-12
1750. C732CO3 + C10H17O8O2 → C18H30O14	3E-12
1751. C8BCO2 + C10H17O8O2 → C18H30O10	3E-12
1752. C88O2 + C10H17O8O2 → C18H30O12	3E-12
1753. C718CO3 + C10H17O8O2 → C18H30O13	3E-12
1754. C87O2 + C10H17O8O2 → C18H30O13	3E-12
1755. C721CO3 + C10H17O8O2 → C18H30O13	3E-12
1756. NC826O2 + C10H17O8O2 → C18H31O12NO3	3E-12

Autoxidation peroxy radicals reacting with other peroxy radicals with different branching ratios for a, b and c:

$$\mathbf{R_1O_2 + R_2O_2 \rightarrow R_1C{=}O + R_2OH} \quad \textbf{(a)}$$
$$\mathbf{\rightarrow R_1OH + R_2C{=}O} \quad \textbf{(b)}$$
$$\mathbf{\rightarrow R_1O + R_2O + O_2} \quad \textbf{(c)}$$

1757. C10H17O3O2 → C10H16O4iso2	5E-12*0.2*RO2	RO2 = $\sum RO_2$ in MCM
1758. C10H17O3O2 → C10H17O3O	5E-12*0.6*RO2	RO2 = $\sum RO_2$ in MCM
1759. C10H17O3O2 → C10H18O4	5E-12*0.2*RO2	RO2 = $\sum RO_2$ in MCM
1760. C10H17O4O2 → C10H16O5iso2	5E-12*0.2*RO2	RO2 = $\sum RO_2$ in MCM
1761. C10H17O4O2 → C10H17O4O	5E-12*0.6*RO2	RO2 = $\sum RO_2$ in MCM
1762. C10H17O4O2 → C10H18O5	5E-12*0.2*RO2	RO2 = $\sum RO_2$ in MCM
1763. C10H17O5O2 → C10H16O6iso2	8E-12*0.2*RO2	RO2 = $\sum RO_2$ in MCM
1764. C10H17O5O2 → C10H17O5O	8E-12*0.6*RO2	RO2 = $\sum RO_2$ in MCM
1765. C10H17O5O2 → C10H18O6	8E-12*0.2*RO2	RO2 = $\sum RO_2$ in MCM
1766. C10H17O6O2 → C10H16O7iso2	1E-11*0.3*RO2	RO2 = $\sum RO_2$ in MCM

1767. C10H17O6O2 → C10H17O6O	1E-11*0.4*RO2	RO2 = $\sum RO_2$ in MCM
1768. C10H17O6O2 → C10H18O7	1E-11*0.3*RO2	RO2 = $\sum RO_2$ in MCM
1769. C10H17O7O2 → C10H16O8iso2	1E-11*0.4*RO2	RO2 = $\sum RO_2$ in MCM
1770. C10H17O7O2 → C10H17O7O	1E-11*0.2*RO2	RO2 = $\sum RO_2$ in MCM
1771. C10H17O7O2 → C10H18O8	1E-11*0.4*RO2	RO2 = $\sum RO_2$ in MCM
1772. C10H17O8O2 → C10H16O9iso2	1E-11*0.5*RO2	RO2 = $\sum RO_2$ in MCM
1773. C10H17O8O2 → C10H18O9	1E-11*0.5*RO2	RO2 = $\sum RO_2$ in MCM

Supplementary Table 5. Correlation coefficients (*R*), normalized mean bias (*NMB*) (%) and the fraction of predictions within a factor of two of the observations (*FAC2*) for the control run (CTRL) and a simulation without dry deposition losses of HOM (NoDep).

Species	*R* CTRL	*NMB* CTRL	*FAC2* CTRL	*R* NoDep	*NMB* NoDep	*FAC2* NoDep
Tot. HOM	0.72	8	0.93	0.73	50	0.78
Monomers	0.72	3	0.93	0.70	49	0.78
Dimers	0.73	-16	0.55	0.73	16	0.67
HOM-NO_3	0.70	-11	0.66	0.75	88	0.55
HOM RO_2	0.60	67	0.61	0.60	75	0.55

Supplementary Table 6. Estimated Henry's law coefficients for all peroxy radical autoxidation products in PRAM[1].

**PRAM name**	**$\log_{10}$(H (M/atm)) SIMPOL[5] + AIOMFAC[7]**	**$\log_{10}$(H (M/atm)) COSMO[8-11]**
C10H15O2O2	3.17	
C10H15O3O2	4.73	
C10H15O4O2	5.95	
C10H15O5O2	7.44	
C10H15O6O2	7.77	
C10H15O7O2	9.16	
C10H15O8O2	10.56	
C10H15O9O2	11.94	
C10H15O10O2	13.43	
C10H15O11O2	14.61	
C10H15O12O2	15.49	
C10H15O2O	3.17	
C10H15O3O	4.73	
C10H15O4O	5.95	
C10H15O5O	7.44	
C10H15O6O	7.77	
C10H15O7O	9.16	
C10H15O8O	10.56	
C10H15O9O	11.94	
C10H15O10O	13.43	
C10H15O11O	14.61	
C10H15O12O	15.49	
C10H14O3	5.17	
C10H14O4	6.29	
C10H14O5	7.52	
C10H14O6	8.94	
C10H14O7	9.22	
C10H14O8	10.58	
C10H14O9	11.86	

C10H14O10	13.25	
C10H14O11	14.66	
C10H14O12	15.90	
C10H14O13	16.61	
C10H16O3	6.34	
C10H16O4iso1	9.11	
C10H16O5iso1	8.71	
C10H16O6iso1	10.30	8.15[a], 10.03[b]
C10H16O7iso1	10.45	
C10H16O8iso1	11.87	
C10H16O9iso1	13.09	
C10H16O10	14.54	
C10H16O11	15.82	
C10H16O12	17.23	
C10H16O13	17.76	
C10H16O14	18.33	
C10H15O2NO3	4.64	
C10H15O3NO3	6.13	
C10H15O4NO3	8.30	
C10H15O5NO3	8.89	
C10H15O6NO3	9.53	
C10H15O7NO3	10.83	
C10H15O8NO3	12.29	
C10H15O9NO3	13.59	
C10H15O10NO3	15.17	
C10H15O11NO3	16.36	
C10H15O12NO3	17.45	
C20H30O5	6.85	
C20H30O6	7.14	
C20H30O7	10.19	
C20H30O8	9.55	
C20H30O9	11.93	
C20H30O10	11.58	
C20H30O11	14.64	
C20H30O12	14.42	
C20H30O13	17.27	
C20H30O14	17.13	
C20H30O15	20.65	
C20H30O16	19.16	
C20H30O17	21.91	
C20H31O4NO3	4.81	
C20H31O5NO3	6.56	
C20H31O6NO3	7.85	
C20H31O7NO3	11.37	
C20H31O8NO3	10.11	
C20H31O9NO3	13.12	

C20H31O10NO3	13.07
C20H31O11NO3	15.96
C20H31O12NO3	16.01
C20H31O13NO3	16.86
C20H31O14NO3	18.59
C20H31O15NO3	19.52
C19H28O5	6.82
C19H28O6	7.10
C19H28O7	10.13
C19H28O8	9.50
C19H28O9	11.84
C19H28O10	11.50
C19H28O11	14.54
C19H28O12	14.32
C19H28O13	7.15
C19H28O14	17.01
C19H28O15	20.50
C19H28O16	19.02
C19H28O17	21.76
C19H28O18	20.68
C19H29O5NO3	6.53
C19H29O6NO3	7.81
C19H29O7NO3	11.32
C19H29O8NO3	10.05
C19H29O9NO3	13.05
C19H29O10NO3	13.00
C19H29O11NO3	15.87
C19H29O12NO3	15.93
C19H29O13NO3	16.76
C19H29O14NO3	18.48
C19H29O15NO3	19.42
C18H26O4	4.61
C18H26O5	6.79
C18H26O6	7.05
C18H26O7	10.06
C18H26O8	9.44
C18H26O9	11.74
C18H26O10	11.41
C18H26O11	14.43
C18H26O12	14.21
C18H26O13	17.02
C18H26O14	16.89
C18H26O15	20.33
C18H26O16	18.87
C18H26O17	21.59
C18H26O18	20.52

C18H27O6NO3	7.39	
C18H27O7NO3	10.89	
C18H27O8NO3	9.63	
C18H27O9NO3	12.62	
C18H27O10NO3	12.57	
C18H27O11NO3	15.45	
C18H27O12NO3	15.50	
C18H27O13NO3	16.33	
C18H27O14NO3	18.05	
C18H27O15NO3	18.99	
C18H27O16NO3	20.49	
C10H17O3O2	5.54	
C10H17O4O2	8.49	
C10H17O5O2	5.21	
C10H17O6O2	8.38	
C10H17O7O2	9.06	
C10H17O8O2	11,73	
C10H17O3O	5.54	
C10H17O4O	8.49	
C10H17O5O	5.21	
C10H17O6O	8.38	
C10H17O7O	9.06	
C10H16O4iso2	8.09	
C10H16O5iso2	10.68	
C10H16O6iso2	7.85	7.30[a], 9.82[b]
C10H16O7iso2	10.55	
C10H16O8iso2	11.24	
C10H16O9iso2	13.74	
C10H18O4	10.17	
C10H18O5	11.37	
C10H18O6	8.82	9.24[a], 11.86[b]
C10H18O7	11.29	
C10H18O8	11.03	
C10H18O9	11.04	
C10H18O10	13.90	
C10H17O3NO3	7.26	
C10H17O4NO3	10.46	
C10H17O5NO3	7.17	
C10H17O6NO3	10.27	
C10H17O7NO3	10.04	
C10H17O8NO3	13.01	
C20H34O6	7.43	
C20H34O7	9.08	
C20H34O8	7.14	
C20H34O9	11.50	
C20H34O10	11.26	

C20H34O11	14.22
C20H34O12	17.17
C20H34O13	16.97
C20H35O5NO3	5.58
C20H35O6NO3	9.17
C20H35O7NO3	10.27
C20H35O8NO3	8.99
C20H35O9NO3	12.37
C20H35O10NO3	11,92
C20H35O11NO3	14.10
C19H32O6	7.41
C19H32O7	9.05
C19H32O8	7.11
C19H32O9	11.42
C19H32O10	11.18
C19H32O11	14.12
C19H32O12	17.05
C19H32O13	16.86
C19H32O14	19.05
C19H33O6NO3	9.14
C19H33O7NO3	10.77
C19H33O8NO3	8.96
C19H33O9NO3	12.32
C19H33O10NO3	11.87
C19H33O11NO3	14.05
C18H30O5	6.45
C18H30O6	7.38
C18H30O7	9.01
C18H30O8	7.08
C18H30O9	11.34
C18H30O10	11.09
C18H30O11	14.02
C18H30O12	17.24
C18H30O13	16.75
C18H30O14	18.94
C18H31O7NO3	10.73
C18H31O8NO3	8.92
C18H31O9NO3	12.27
C18H31O10NO3	11.82
C18H31O11NO3	14.00
C18H31O12NO3	14.25

a) Using all conformers
b) Using only conformers with the minimum number of H-bonds (0 full and 0 partial H-bonds for $C_{10}H_{16}O_6$iso1 and $C_{10}H_{16}O_6$iso2; 0 full and 1 partial H-bond for $C_{10}H_{18}O_6$).

Supplementary Table 7. List of all reactions and their reaction rates in an alternative reduced PRAM[1] version without explicit representation of individual HOM dimer species formation.

Initial Criegiee intermediate unimolecular reactions to peroxy radicals via the vinylhydroperoxide pathway[5] which continue to react according to the original MCMv3.3.1 mechanism (R1-R2, R4-R5) or initialize the peroxy radical autoxidation in PRAM (R3 and R6)

1. APINOOA → C107O2 + OH	KDEC*0.495	To MCM chem.
2. APINOOA → C109O2 + OH	KDEC*0.405	To MCM chem.
3. APINOOA → C10H15O2O2 + OH	KDEC*0.10	To PRAM
4. LIMOOA → LIMALAO2 + OH	KDEC*0.35	To MCM chem.
5. LIMOOA → LIMALBO2 + OH	KDEC*0.35	To MCM chem.
6. LIMOOA→ C10H15O2O2 + OH	KDEC*0.30	To PRAM

Lumped reactions describing the initial reactions between a monoterpene (MT) and OH forming a RO_2 with 3 oxygen atoms ($C_{10}H_{17}OO_2$) that rapidly undergo a first intramolecular hydrogen shift and O_2 addition and form a new RO_2 with 5 oxygen atoms ($C_{10}H_{17}O_3O_2$).
MT + OH → $C_{10}H_{17}OO_2$
$C_{10}H_{17}OO_2$ + O_2 → $C_{10}H_{17}O_3O_2$ (Fast)

7. APINENE + OH → C10H17O3O2	0.025*1.2E-11*EXP(440/T)	To PRAM
8. BPINENE + OH → C10H17O3O2	0.01*2.38E-11*EXP(357/T)	To PRAM
9. LIMONENE + OH → C10H17O3O2	0.01*4.28E-11*EXP(401/T)	To PRAM
10. CARENE + OH → C10H17O3O2	0.025*1.6E-11*EXP(500/T)	To PRAM

Peroxy radical autoxidation and HOM formation after O_3 oxidation of monoterpenes (R11-R1154)

Autoxidation reaction rates (H-shifts + O_2)

11. C10H15O2O2 → C10H15O4O2	1.2E17*EXP(-12077/T)	Unit s^{-1}
12. C10H15O3O2 → C10H15O5O2	1.2E18*EXP(-12077/T)	Unit s^{-1}
13. C10H15O4O2 → C10H15O6O2	1.2E18*EXP(-12077/T)	Unit s^{-1}
14. C10H15O5O2 → C10H15O7O2	6E17*EXP(-12077/T)	Unit s^{-1}
15. C10H15O6O2 → C10H15O8O2	2E17*EXP(-12077/T)	Unit s^{-1}
16. C10H15O7O2 → C10H15O9O2	1.2E17*EXP(-12077/T)	Unit s^{-1}
17. C10H15O8O2 → C10H15O10O2	1.2E17*EXP(-12077/T)	Unit s^{-1}
18. C10H15O9O2 → C10H15O11O2	1.2E16*EXP(-12077/T)	Unit s^{-1}
19. C10H15O10O2 → C10H15O12O2	0.0	Unit s^{-1}

Isomerization of alkoxy radicals (RO) followed by reaction with O_2 to make new peroxy radicals:
RO· → ·ROH
·ROH + O_2 → RO_2·
Net: RO· + O_2 → RO_2·

20. C10H15O2O → C10H15O3O2	KDEC	Unit s^{-1}
21. C10H15O3O → C10H15O4O2	KDEC	Unit s^{-1}
22. C10H15O4O → C10H15O5O2	KDEC	Unit s^{-1}
23. C10H15O5O → C10H15O6O2	KDEC	Unit s^{-1}
24. C10H15O6O → C10H15O7O2	KDEC	Unit s^{-1}
25. C10H15O7O → C10H15O8O2	KDEC	Unit s^{-1}
26. C10H15O8O → C10H15O9O2	KDEC	Unit s^{-1}
27. C10H15O9O → C10H15O10O2	KDEC	Unit s^{-1}
28. C10H15O10O → C10H15O11O2	KDEC	Unit s^{-1}
29. C10H15O11O → C10H15O12O2	KDEC	Unit s^{-1}

Peroxy radicals reacting with NO and forming an alkoxy radical and NO_2 (RO_2 + NO → RO + NO_2)
*The rate for the reactions between autoxidation RO_2 and NO was set to the same values used in MCMv3.3.1 (*KRO2NO)

30. C10H15O2O2 + NO → C10H15O2O + NO_2	KRO2NO*1.0
31. C10H15O3O2 + NO → C10H15O3O + NO_2	KRO2NO*1.0
32. C10H15O4O2 + NO → C10H15O4O + NO_2	KRO2NO*1.0
33. C10H15O5O2 + NO → C10H15O5O + NO_2	KRO2NO*0.9
34. C10H15O6O2 + NO → C10H15O6O + NO_2	KRO2NO*0.85
35. C10H15O7O2 + NO → C10H15O7O + NO_2	KRO2NO*0.8
36. C10H15O8O2 + NO → C10H15O8O + NO_2	KRO2NO*0.6
37. C10H15O9O2 + NO → C10H15O9O + NO_2	KRO2NO*0.5
38. C10H15O10O2 + NO → C10H15O10O + NO_2	KRO2NO*0.3
39. C10H15O11O2 + NO → C10H15O11O + NO_2	0.0
40. C10H15O12O2 + NO → C10H15O12O + NO_2	0.0

Lumped reactions describing the fragmentation of autoxidation products when they react with NO (RO_2 + NO → RO + NO_2) followed by fast fragmentation of the formed alkoxy radicals).
The fragmentation products are assumed to be the MCM compounds C717O2 (a peroxy radical) and CH3COCH3 (acetone) irrespectively of which autoxidation peroxy radical that react with NO. This is a simplification that violates the mass balance for O atoms but not for the N and C atoms.

41. C10H15O2O2 + NO → C717O2 + CH3COCH3 + NO2	0.0
42. C10H15O3O2 + NO → C717O2 + CH3COCH3+NO2	0.0
43. C10H15O4O2 + NO → C717O2 + CH3COCH3 + NO2	0.0
44. C10H15O5O2 + NO → C717O2 + CH3COCH3+NO2	KRO2NO*0.04
45. C10H15O6O2 + NO → C717O2 + CH3COCH3 + NO2	KRO2NO*0.06

46. C10H15O7O2 + NO → C717O2 + CH3COCH3+NO2	KRO2NO*0.08
47. C10H15O8O2 + NO → C717O2 + CH3COCH3 + NO2	KRO2NO*0.16
48. C10H15O9O2 + NO → C717O2 + CH3COCH3+NO2	KRO2NO*0.20
49. C10H15O10O2 + NO → C717O2 + CH3COCH3 + NO2	KRO2NO*0.28
50. C10H15O11O2 + NO → C717O2 + CH3COCH3+NO2	KRO2NO*0.40
51. C10H15O12O2 + NO → C717O2 + CH3COCH3+NO2	KRO2NO*0.40

Peroxy radicals reacting with NO forming monomers with an additional carbonyl group:
RO_2 + NO → RO + NO_2
RO + O_2 → RCHO + HO_2 (Fast)
Net: RO_2 + NO + O_2→ RCHO + NO_2 + HO_2

52. C10H15O2O2 + NO → C10H14O3 + NO_2 + HO_2	0.0
53. C10H15O3O2 + NO → C10H14O4 + NO_2 + HO_2	0.0
54. C10H15O4O2 + NO → C10H14O5 + NO_2 + HO_2	0.0
55. C10H15O5O2 + NO → C10H14O6 + NO_2 + HO_2	KRO2NO*0.042
56. C10H15O6O2 + NO → C10H14O7 + NO_2 + HO_2	KRO2NO*0.063
57. C10H15O7O2 + NO → C10H14O8 + NO_2 + HO_2	KRO2NO*0.084
58. C10H15O8O2 + NO → C10H14O9 + NO_2 + HO_2	KRO2NO*0.168
59. C10H15O9O2 + NO → C10H14O10 + NO_2 + HO_2	KRO2NO*0.210
60. C10H15O10O2 + NO → C10H14O11 + NO_2 + HO_2	KRO2NO*0.294
61. C10H15O11O2 + NO → C10H14O12 + NO_2 + HO_2	KRO2NO*0.420
62. C10H15O12O2 + NO → C10H14O13 + NO_2 + HO_2	KRO2NO*0.420

Peroxy radicals reacting with NO forming compounds with a nitrate functional group (RO_2 + NO → RNO_3)

63. C10H15O2O2 + NO → C10H15O2NO3	0.0
64. C10H15O3O2 + NO → C10H15O3NO3	0.0
65. C10H15O4O2 + NO → C10H15O4NO3	0.0
66. C10H15O5O2 + NO → C10H15O5NO3	KRO2NO*0.018
67. C10H15O6O2 + NO → C10H15O6NO3	KRO2NO*0.027
68. C10H15O7O2 + NO → C10H15O7NO3	KRO2NO*0.036
69. C10H15O8O2 + NO → C10H15O8NO3	KRO2NO*0.072
70. C10H15O9O2 + NO → C10H15O9NO3	KRO2NO*0.090
71. C10H15O10O2 + NO → C10H15O10NO3	KRO2NO*0.126
72. C10H15O11O2 + NO → C10H15O11NO3	KRO2NO*0.180
73. C10H15O12O2 + NO → C10H15O12NO3	KRO2NO*0.180

Peroxy radicals reacting with HO_2 forming compounds with a hydrogen peroxide functional group (RO_2 + HO_2 → ROOH + O_2)

74. C10H15O2O2 + HO2 → C10H16O4iso1	KRO2HO2
75. C10H15O3O2 + HO2 → C10H16O5iso1	KRO2HO2
76. C10H15O4O2 + HO2 → C10H16O6iso1	KRO2HO2
77. C10H15O5O2 + HO2 → C10H16O7iso1	KRO2HO2
78. C10H15O6O2 + HO2 → C10H16O8iso1	KRO2HO2
79. C10H15O7O2 + HO2 → C10H16O9iso1	KRO2HO2
80. C10H15O8O2 + HO2 → C10H16O10	KRO2HO2
81. C10H15O9O2 + HO2 → C10H16O11	KRO2HO2
82. C10H15O10O2 + HO2 → C10H16O12	KRO2HO2
83. C10H15O11O2 + HO2 → C10H16O13	KRO2HO2
84. C10H15O12O2 + HO2 → C10H16O14	KRO2HO2

Peroxy radicals reacting with other peroxy radicals forming dimers (RO_2 + RO_2 → ROOR + O_2)

85. C10H15O2O2 → C20H30O13	1E-13*RO2	RO2 = ∑ RO_2 in MCM
86. C10H15O3O2 → C20H30O13	1E-13*RO2	RO2 = ∑ RO_2 in MCM
87. C10H15O4O2 → C20H30O13	1E-13*RO2	RO2 = ∑ RO_2 in MCM
88. C10H15O5O2 → C20H30O13	5E-13*RO2	RO2 = ∑ RO_2 in MCM
89. C10H15O6O2 → C20H30O13	2E-12*RO2	RO2 = ∑ RO_2 in MCM
90. C10H15O7O2 → C20H30O13	3E-12*RO2	RO2 = ∑ RO_2 in MCM
91. C10H15O8O2 → C20H30O13	3E-12*RO2	RO2 = ∑ RO_2 in MCM
92. C10H15O9O2 → C20H30O13	5E-12*RO2	RO2 = ∑ RO_2 in MCM
93. C10H15O10O2 → C20H30O13	5E-12*RO2	RO2 = ∑ RO_2 in MCM
94. C10H15O11O2 → C20H30O13	5E-12*RO2	RO2 = ∑ RO_2 in MCM
95. C10H15O12O2 → C20H30O13	5E-12*RO2	RO2 = ∑ RO_2 in MCM

Autoxidation peroxy radicals reacting with other peroxy radicals with different branching ratios for a, b and c:

R_1O_2 + R_2O_2 → R_1C=O + R_2OH (a)
→ R_1OH + R_2C=O (b)
→ R_1O + R_2O + O_2 (c)

96. C10H15O2O2 → C10H14O3	1E-12*0.2*RO2	RO2 = ∑ RO_2 in MCM
97. C10H15O2O2 → C10H15O2O	1E-12*0.6*RO2	RO2 = ∑ RO_2 in MCM
98. C10H15O2O2 → C10H16O3	1E-12*0.2*RO2	RO2 = ∑ RO_2 in MCM
99. C10H15O3O2 → C10H14O4	5E-12*0.2*RO2	RO2 = ∑ RO_2 in MCM
100. C10H15O3O2 → C10H15O3O	5E-12*0.6*RO2	RO2 = ∑ RO_2 in MCM
101. C10H15O3O2 → C10H16O4iso1	5E-12*0.2*RO2	RO2 = ∑ RO_2 in MCM

102. C10H15O4O2 → C10H14O5	5E-12*0.2*RO2	RO2 = ∑ RO_2 in MCM
103. C10H15O4O2 → C10H15O4O	5E-12*0.6*RO2	RO2 = ∑ RO_2 in MCM
104. C10H15O4O2 → C10H16O5iso1	5E-12*0.2*RO2	RO2 = ∑ RO_2 in MCM
105. C10H15O5O2 → C10H14O6	5E-12*0.2*RO2	RO2 = ∑ RO_2 in MCM
106. C10H15O5O2 → C10H15O5O	5E-12*0.6*RO2	RO2 = ∑ RO_2 in MCM
107. C10H15O5O2 → C10H16O6iso1	5E-12*0.2*RO2	RO2 = ∑ RO_2 in MCM
108. C10H15O6O2 → C10H14O7	7E-12*0.4*RO2	RO2 = ∑ RO_2 in MCM
109. C10H15O6O2 → C10H15O6O	7E-12*0.4*RO2	RO2 = ∑ RO_2 in MCM
110. C10H15O6O2 → C10H16O7iso1	7E-12*0.2*RO2	RO2 = ∑ RO_2 in MCM
111. C10H15O7O2 → C10H14O8	8E-12*0.2*RO2	RO2 = ∑ RO_2 in MCM
112. C10H15O7O2 → C10H15O7O	8E-12*0.4*RO2	RO2 = ∑ RO_2 in MCM
113. C10H15O7O2 → C10H16O8iso1	8E-12*0.4*RO2	RO2 = ∑ RO_2 in MCM
114. C10H15O8O2 → C10H14O9	9E-12*0.4*RO2	RO2 = ∑ RO_2 in MCM
115. C10H15O8O2 → C10H15O8O	9E-12*0.2*RO2	RO2 = ∑ RO_2 in MCM
116. C10H15O8O2 → C10H16O9iso1	9E-12*0.4*RO2	RO2 = ∑ RO_2 in MCM
117. C10H15O9O2 → C10H14O10	1E-11*0.3*RO2	RO2 = ∑ RO_2 in MCM
118. C10H15O9O2 → C10H15O9O	1E-11*0.2*RO2	RO2 = ∑ RO_2 in MCM
119. C10H15O9O2 → C10H16O10	1E-11*0.5*RO2	RO2 = ∑ RO_2 in MCM
120. C10H15O10O2 → C10H14O11	1E-11*0.5*RO2	RO2 = ∑ RO_2 in MCM
121. C10H15O10O2 → C10H15O10O	1E-11*0.2*RO2	RO2 = ∑ RO_2 in MCM
122. C10H15O10O2 → C10H16O11	1E-11*0.3*RO2	RO2 = ∑ RO_2 in MCM
123. C10H15O11O2 → C10H14O12	1E-11*0.4*RO2	RO2 = ∑ RO_2 in MCM
124. C10H15O11O2 → C10H15O11O	1E-11*0.2*RO2	RO2 = ∑ RO_2 in MCM
125. C10H15O11O2 → C10H16O12	1E-11*0.4*RO2	RO2 = ∑ RO_2 in MCM
126. C10H15O12O2 → C10H14O13	1E-11*0.5*RO2	RO2 = ∑ RO_2 in MCM
127. C10H15O12O2 → C10H15O12O	1E-11*0.0*RO2	RO2 = ∑ RO_2 in MCM
128. C10H15O12O2 → C10H16O13	1E-11*0.5*RO2	RO2 = ∑ RO_2 in MCM
129. C107O2 → C10H15O2O	9.4E-14*RO2*0.1	To PRAM, RO2 = ∑ RO_2 in MCM
130. C107O2 → C107O	9.4E-14*RO2*0.63	To MCM, RO2 = ∑ RO_2 in MCM
131. C107O2 → C107OH	9.4E-14*RO2*0.27	To MCM, RO2 = ∑ RO_2 in MCM

Peroxy radical autoxidation and HOM formation after OH oxidation of monoterpenes (R132-R192)

Autoxidation reaction rates (H-shifts + O_2)

132. C10H17O3O2 → C10H17O5O2	2E17*EXP(-12077/T)	Unit s^{-1}
133. C10H17O4O2 → C10H17O6O2	6E16*EXP(-12077/T)	Unit s^{-1}
134. C10H17O5O2 → C10H17O7O2	6E16*EXP(-12077/T)	Unit s^{-1}
135. C10H17O6O2 → C10H17O8O2	3E16*EXP(-12077/T)	Unit s^{-1}

Isomerization of alkoxy radicals (RO) followed by reaction with O_2 to make new peroxy radicals (RO· →· ROH, · ROH + O_2 → RO_2)

136. C10H17O3O → C10H17O4O2	KDEC	Unit s^{-1}
137. C10H17O4O → C10H17O5O2	KDEC	Unit s^{-1}
138. C10H17O5O → C10H17O6O2	KDEC	Unit s^{-1}
139. C10H17O6O → C10H17O7O2	KDEC	Unit s^{-1}
140. C10H17O7O → C10H17O8O2	KDEC	Unit s^{-1}

Peroxy radicals reacting with NO and forming an alkoxy radical and NO_2 (RO_2 + NO → RO + NO_2)

*The rate for the reactions between autoxidation RO_2 and NO was set to the same values used in MCMv3.3.1 (*KRO2NO)

141. C10H17O3O2 + NO → C10H17O3O + NO2	KRO2NO*1.0
142. C10H17O4O2 + NO → C10H17O4O + NO2	KRO2NO*0.9
143. C10H17O5O2 + NO → C10H17O5O + NO2	KRO2NO*0.8
144. C10H17O6O2 + NO → C10H17O6O + NO2	KRO2NO*0.7
145. C10H17O7O2 + NO → C10H17O7O + NO2	KRO2NO*0.5

Lumped reactions describing the fragmentation of autoxidation products when they react with NO (RO_2 + NO → RO + NO_2) followed by fast fragmentation of the formed alkoxy radicals).

The fragmentation products are assumed to be the MCM compounds C717O2 (a peroxy radical) and CH3COCH3 (acetone) irrespectively of which autoxidation peroxy radical that react with NO. This is a simplification that violates the mass balance for O atoms but not for the N and C atoms.

146. C10H17O3O2 + NO → C717O2 + CH3COCH3 + NO2	0.0
147. C10H17O4O2 + NO → C717O2 + CH3COCH3 + NO2	KRO2NO*0.04
148. C10H17O5O2 + NO → C717O2 + CH3COCH3 + NO2	KRO2NO*0.08
149. C10H17O6O2 + NO → C717O2 + CH3COCH3 + NO2	KRO2NO*0.12
150. C10H17O7O2 + NO → C717O2 + CH3COCH3 + NO2	KRO2NO*0.20
151. C10H17O8O2 + NO → C717O2 + CH3COCH3 + NO2	KRO2NO*0.40

Peroxy radicals reacting with NO forming monomers with an additional carbonyl group:
$RO_2 + NO \rightarrow RO + NO_2$
$RO + O_2 \rightarrow RCHO + HO_2$ (Fast)
Net: $RO_2 + NO + O_2 \rightarrow RCHO + NO_2 + HO_2$

152. C10H17O3O2 + NO → C10H16O4iso2 + NO2 + HO2	0.0
153. C10H17O4O2 + NO → C10H16O5iso2 + NO2 + HO2	KRO2NO*0.042
154. C10H17O5O2 + NO → C10H16O6iso2 + NO2 + HO2	KRO2NO*0.084
155. C10H17O6O2 + NO → C10H16O7iso2 + NO2 + HO2	KRO2NO*0.126
156. C10H17O7O2 + NO → C10H16O8iso2 + NO2 + HO2	KRO2NO*0.21
157. C10H17O8O2 + NO → C10H16O9iso2 + NO2 + HO2	KRO2NO*0.42

Peroxy radicals reacting with NO forming compounds with a nitrate functional group ($RO_2 + NO \rightarrow RNO_3$)

158. C10H17O3O2 + NO → C10H17O3NO3	0.0
159. C10H17O4O2 + NO → C10H17O4NO3	KRO2NO*0.018
160. C10H17O5O2 + NO → C10H17O5NO3	KRO2NO*0.036
161. C10H17O6O2 + NO → C10H17O6NO3	KRO2NO*0.054
162. C10H17O7O2 + NO → C10H17O7NO3	KRO2NO*0.090
163. C10H17O8O2 + NO → C10H17O8NO3	KRO2NO*0.180

Peroxy radicals reacting with HO_2 forming compounds with an hydrogen peroxide functional group ($RO_2 + HO_2 \rightarrow ROOH + O_2$)

164. C10H17O3O2 + HO2 → C10H18O5	KRO2HO2
165. C10H17O4O2 + HO2 → C10H18O6	KRO2HO2
166. C10H17O5O2 + HO2 → C10H18O7	KRO2HO2
167. C10H17O6O2 + HO2 → C10H18O8	KRO2HO2
168. C10H17O7O2 + HO2 → C10H18O9	KRO2HO2
169. C10H17O8O2 + HO2 → C10H18O10	KRO2HO2

Peroxy radicals reacting with other peroxy radicals forming dimers ($RO_2 + RO_2 \rightarrow ROOR + O_2$)

170. C10H17O3O2 → C20H34O10	1E-13*RO2	RO2 = ∑ RO_2 in MCM
171. C10H17O4O2 → C20H34O10	1E-13*RO2	RO2 = ∑ RO_2 in MCM
172. C10H17O5O2 → C20H34O10	5E-13*RO2	RO2 = ∑ RO_2 in MCM
173. C10H17O6O2 → C20H34O10	5E-13*RO2	RO2 = ∑ RO_2 in MCM
174. C10H17O7O2 → C20H34O10	5E-13*RO2	RO2 = ∑ RO_2 in MCM
175. C10H17O8O2 → C20H34O10	5E-13*RO2	RO2 = ∑ RO_2 in MCM

Autoxidation peroxy radicals reacting with other peroxy radicals with different branching ratios for a, b and c:

$R_1O_2 + R_2O_2 \rightarrow R_1C{=}O + R_2OH$ (a)
$\rightarrow R_1OH + R_2C{=}O$ (b)
$\rightarrow R_1O + R_2O + O_2$ (c)

176. C10H17O3O2 → C10H16O4iso2	5E-12*0.2*RO2	RO2 = ∑ RO_2 in MCM
177. C10H17O3O2 → C10H17O3O	5E-12*0.6*RO2	RO2 = ∑ RO_2 in MCM
178. C10H17O3O2 → C10H18O4	5E-12*0.2*RO2	RO2 = ∑ RO_2 in MCM
179. C10H17O4O2 → C10H16O5iso2	5E-12*0.2*RO2	RO2 = ∑ RO_2 in MCM
180. C10H17O4O2 → C10H17O4O	5E-12*0.6*RO2	RO2 = ∑ RO_2 in MCM
181. C10H17O4O2 → C10H18O5	5E-12*0.2*RO2	RO2 = ∑ RO_2 in MCM
182. C10H17O5O2 → C10H16O6iso2	8E-12*0.2*RO2	RO2 = ∑ RO_2 in MCM
183. C10H17O5O2 → C10H17O5O	8E-12*0.6*RO2	RO2 = ∑ RO_2 in MCM
184. C10H17O5O2 → C10H18O6	8E-12*0.2*RO2	RO2 = ∑ RO_2 in MCM
185. C10H17O6O2 → C10H16O7iso2	1E-11*0.3*RO2	RO2 = ∑ RO_2 in MCM
186. C10H17O6O2 → C10H17O6O	1E-11*0.4*RO2	RO2 = ∑ RO_2 in MCM
187. C10H17O6O2 → C10H18O7	1E-11*0.3*RO2	RO2 = ∑ RO_2 in MCM
188. C10H17O7O2 → C10H16O8iso2	1E-11*0.4*RO2	RO2 = ∑ RO_2 in MCM
189. C10H17O7O2 → C10H17O7O	1E-11*0.2*RO2	RO2 = ∑ RO_2 in MCM
190. C10H17O7O2 → C10H18O8	1E-11*0.4*RO2	RO2 = ∑ RO_2 in MCM
191. C10H17O8O2 → C10H16O9iso2	1E-11*0.5*RO2	RO2 = ∑ RO_2 in MCM
192. C10H17O8O2 → C10H18O9	1E-11*0.5*RO2	RO2 = ∑ RO_2 in MCM

Supplementary Table 8. Observed average HOM concentrations ($\bar{O}$) (ppt$_v$) and modelled average HOM concentrations ($\bar{M}$) (ppt$_v$), correlation coefficients (R), normalized mean bias (*NMB*) and the fraction of predictions within a factor of two of the observations (*FAC2*) at SMEAR II May 15-24, 2013. The model results from the PRAM sensitivity tests with the default PRAM version (Supplementary Table 4), PRAM with k(RO_2 +RO_2)x2, PRAM with k(RO_2 +RO_2)x0.5, PRAM with $E_{H\text{-}shift}$ = 50 kJ, PRAM with $E_{H\text{-}shift}$ = 150 kJ and the reduced PRAM version (Supplementary Table 7) without explicit representation of individual HOM dimer species are all evaluated against the observations.

Statistics	Tot. HOM	Monomers	Dimers	HOM-NO_3	HOM RO_2
$\bar{O}$	0.54	0.33	0.09	0.04	0.08
$\bar{M}$ (Default)	0.58	0.34	0.07	0.04	0.13
$\bar{M}$ k(RO_2 + RO_2)x2	0.61	0.40	0.09	0.03	0.09
$\bar{M}$ k(RO_2 + RO_2)x0.5	0.55	0.30	0.06	0.04	0.16
$\bar{M}$ $E_{H\text{-}shift}$ = 50 kJ	0.58	0.34	0.07	0.04	0.13
$\bar{M}$ $E_{H\text{-}shift}$ = 150 kJ	0.58	0.35	0.07	0.04	0.12
$\bar{M}$ reduced PRAM	0.58	0.34	0.08	0.04	0.12
R (Default)	0.72	0.72	0.73	0.70	0.60
R k(RO_2 + RO_2)x2	0.71	0.71	0.72	0.70	0.55
R k(RO_2 + RO_2)x0.5	0.73	0.70	0.72	0.69	0.64
R $E_{H\text{-}shift}$ = 50 kJ	0.72	0.72	0.72	0.68	0.61
R $E_{H\text{-}shift}$ = 150 kJ	0.72	0.71	0.72	0.72	0.60
R reduced PRAM	0.72	0.71	0.73	0.70	0.61
NMB (%) (Default)	8	3	-16	-11	67
NMB k(RO_2 + RO_2)x2	14	19	0.2	-18	26
NMB k(RO_2 + RO_2)x0.5	4	-9	-34	-5	110
NMB $E_{H\text{-}shift}$ = 50 kJ	8	2	-14	-11	67
NMB $E_{H\text{-}shift}$ = 150 kJ	8	4	-20	-10	65
NMB reduced PRAM	7	2	-11	-12	62
FAC2 (Default)	0.93	0.93	0.55	0.66	0.61
FAC2 k(RO_2 + RO_2)x2	0.92	0.91	0.67	0.64	0.76
FAC2 k(RO_2 + RO_2)x0.5	0.94	0.90	0.41	0.68	0.41
FAC2 $E_{H\text{-}shift}$ = 50 kJ	0.93	0.93	0.52	0.65	0.61
FAC2 $E_{H\text{-}shift}$ = 150 kJ	0.94	0.94	0.56	0.67	0.61
FAC2 reduced PRAM	0.93	0.93	0.50	0.66	0.62

Supplementary Table 9. Gas-phase precursors used for the ADCHEM model simulations

**Gas-phase precursor**	
α-pinene	*β*-pinene
Limonene	Carene
Isoprene	Ethane
Butane	Etene
Propene	*o*-xylene
Formaldehyde	Acetaldehyde
Methyl Ethyl Ketone	Glyoxal
Methylglyoxal	1-petene
2-methylpropene	Dodecane
Benzene	Decane
Ethylbenzene	Nonane
p-xylene	Toluene
Undecane	*m*-xylene
1-butene	1,2,4-trimethylbenzene
1,3,5-trimethylbenzene	1,2,3-trimethylbenzene
β- caryophyllene	

Supplementary References

1. Roldin, P. Peroxy Radical Autoxidation Mechanism (PRAM). PANGAEA, https://doi.org/10.1594/PANGAEA.905102 (2019).
2. Ehn, M., et al. A large source of low-volatility secondary organic aerosol. *Nature* **506**, 476–479 (2014).
3. Jokinen, T., et al. Production of extremely low volatile organic compounds from biogenic emissions: Measured yields and atmospheric implications. *Proc. Natl. Acad. Sci. USA* **112**, 7123–7128 (2015).
4. Berndt, T., et al. Hydroxyl radical-induced formation of highly oxidized organic compounds. *Nature Communications* **7** (2016).
5. Pankow, J. F., & Asher, W. E. Simpol.1: a simple group contribution method for predicting vapor pressures and enthalpies of vaporization of multifunctional organic compounds. *Atmospheric Chemistry and Physics* **8**, 2773–2796 (2008).
6. Jenkin, M. E., Saunders, S. M., & Pilling, M. J. The tropospheric degradation of volatile organic compounds: a protocol for mechanism development. *Atmospheric Environment* **31**, 81–104 (1997).
7. Zuend, A., et al. New and extended parameterization of the thermodynamic model AIOMFAC: calculation of activity coefficients for organic-inorganic mixtures containing carboxyl, hydroxyl, carbonyl, ether, ester, alkenyl, alkyl, and aromatic functional groups, *Atmos. Chem. Phys.*, **11**, 9155–9206 (2011).
8. Eckert, F., Klamt, A. COSMOTherm, Version C3.0, Release 17.05; *COSMOlogic GmbH & Co. KG*: Leverkusen, Germany (2014).
9. Klamt, A. The COSMO and COSMO-RS solvation models. *Wiley Interdisciplinary Reviews: Computational Molecular Science*, **1**, 699-709 (2011).
10. Tomasi, J., Mennucci, B., & Cammi, R. Quantum Mechanical Continuum Solvation Models. *Chemical Reviews*, **105**, 2999-3094 (2005).
11. Wavefunction, Inc: Spartan ’14 version 1.1.9, Irvine, CA.
